# Multi‐omic profiling reveals an RNA processing rheostat that predisposes to prostate cancer

**DOI:** 10.15252/emmm.202317463

**Published:** 2023-04-24

**Authors:** Maike Stentenbach, Judith A Ermer, Danielle L Rudler, Kara L Perks, Samuel A Raven, Richard G Lee, Tim McCubbin, Esteban Marcellin, Stefan J Siira, Oliver Rackham, Aleksandra Filipovska

**Affiliations:** ^1^ Harry Perkins Institute of Medical Research QEII Medical Centre Nedlands WA Australia; ^2^ ARC Centre of Excellence in Synthetic Biology QEII Medical Centre Nedlands WA Australia; ^3^ Centre for Medical Research The University of Western Australia, QEII Medical Centre Nedlands WA Australia; ^4^ Australian Institute for Bioengineering and Nanotechnology The University of Queensland Brisbane QLD Australia; ^5^ Curtin Medical School Curtin University Bentley WA Australia; ^6^ Curtin Health Innovation Research Institute Curtin University Bentley WA Australia; ^7^ Telethon Kids Institute Northern Entrance, Perth Children's Hospital Nedlands WA Australia

**Keywords:** gene expression, mitochondria, prostate cancer susceptibility, RNA processing, Cancer, Chromatin, Transcription & Genomics, Proteomics

## Abstract

Prostate cancer is the most commonly diagnosed malignancy and the third leading cause of cancer deaths. GWAS have identified variants associated with prostate cancer susceptibility; however, mechanistic and functional validation of these mutations is lacking. We used CRISPR‐Cas9 genome editing to introduce a missense variant identified in the *ELAC2* gene, which encodes a dually localised nuclear and mitochondrial RNA processing enzyme, into the mouse *Elac2* gene as well as to generate a prostate‐specific knockout of *Elac2*. These mutations caused enlargement and inflammation of the prostate and nodule formation. The *Elac2* variant or knockout mice on the background of the transgenic adenocarcinoma of the mouse prostate (TRAMP) model show that *Elac2* mutation with a secondary genetic insult exacerbated the onset and progression of prostate cancer. Multiomic profiling revealed defects in energy metabolism that activated proinflammatory and tumorigenic pathways as a consequence of impaired noncoding RNA processing and reduced protein synthesis. Our physiologically relevant models show that the *ELAC2* variant is a predisposing factor for prostate cancer and identify changes that underlie the pathogenesis of this cancer.

## Graphical abstract

Polymorphisms in ELAC2 have been correlated with prostate cancer, but a definitive link has been lacking. Introducing a common prostate‐susceptibility variant into mice increases inflammation, perturbs the production of non‐coding RNAs, and rewires cellular signaling to exacerbate tumorigenesis.

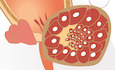

**The paper explained**

**Problem**

The mechanisms that contribute to the occurrence, aetiology and progression of prostate cancer are poorly understood. Despite evidence for a high genetic contribution to prostate cancer, there has not been significant progress in understanding genes involved in this disease. The *ELAC2* gene (hereditary prostate cancer 2 locus, *HPC2*) was the first identified candidate for predisposition to prostate cancer and over 60 studies have linked mutations in *ELAC2* to prostate cancer; however, mechanistic and functional validation of these mutations is lacking.

**Results**

We show that an *ELAC2* variant or loss of *Elac2* is a predisposing factor for prostate cancer and provide detailed molecular changes by which reduced ELAC2 function causes tumorigenesis. Multiomic profiling of our prostate cancer models revealed defects in RNA and energy metabolism that activate proinflammatory and tumorigenic pathways as a consequence of impaired processing of mitochondrial and nuclear‐encoded noncoding RNAs, altered levels of specific pro‐tumorigenic miRNA and reduced protein synthesis. Immunoprecipitation followed by proteomic analyses enabled us to identify a new association between ELAC2 with the splicing factor proline‐ and glutamine‐rich (SFPQ) and non‐POU domain containing octamer binding (NONO) proteins that have previously been shown to sequester mRNAs encoding mitochondrial proteins within the nuclear paraspeckles they form. The human *ELAC2* variant reduces this interaction and contributes to transcriptional activation of tumorigenic pathways that predispose to prostate cancer.

**Impact**

Our findings validate the use of ELAC2 as a clinically relevant biomarker for prostate cancer that in conjunction with current tests may provide more reliable early detection of prostate cancer. Furthermore, we provide new and physiologically relevant preclinical models of prostate inflammation that show defects in immunometabolism in addition to two new models of prostate cancer, which show consistent temporal and developmental prostate tumorigenesis and can be used to screen for new drugs to treat prostate cancer.

## Introduction

Prostate cancer is the most commonly diagnosed malignancy, the second most frequent cancer worldwide and the third leading cause of cancer death (Bray *et al*, 2018). However, the mechanisms that contribute to the occurrence, aetiology and progression of prostate cancer are poorly understood. Multiple genetic and environmental factors have been suggested to have an important role in the onset and progression of prostate cancer (Schaid, 2004), including advanced age, ethnicity, specific gene mutations and family history as well as diet, obesity, pre‐existing illnesses, chemicals or ionising radiation (Bostwick *et al*, 2004; Kolonel *et al*, 2004). Genetic analyses suggest that approximately 20% of prostate cancer cases are familial forms (with at least two first‐degree relatives affected), while only 5% of the disease incidence is caused by hereditary transmission (Carter *et al*, 1992; Cancel‐Tassin & Cussenot, 2005). Numerous genetic and genome‐wide association studies (GWAS) in the last decade have implicated specific genes in the development of sporadic and hereditary prostate cancer (Carpten *et al*, 2002; Rökman *et al*, 2002; Agalliu *et al*, 2010). The hereditary prostate cancer 1 gene (*HPC1*)/Ribonuclease L (*RNASEL*; Carpten *et al*, 2002; Rökman *et al*, 2002; Agalliu *et al*, 2010; 1q24–25) and HPC2 or ElaC Homolog Protein 2 (*ELAC2*; 17p12; Rebbeck *et al*, 2000; Tavtigian *et al*, 2001; Adler *et al*, 2003) are the most relevant candidate genes for prostate cancer predisposition (Alvarez‐Cubero *et al*, 2015).

*ELAC2* was the first identified prostate cancer susceptibility gene (Tavtigian *et al*, 2001) and encodes an RNase Z enzyme, which belongs to the metallo‐β‐lactamase superfamily of proteins found to process the 3′ ends of tRNAs (Takaku *et al*, 2003; Brzezniak *et al*, 2011; Lopez Sanchez *et al*, 2011). *ELAC2* encodes both nuclear and mitochondrial targeted RNase Z enzymes produced by alternative translational initiation (Rossmanith, 2011), and its loss causes embryonic lethality (Siira *et al*, 2018). The role of ELAC2 in processing nuclear and mitochondrial tRNAs at their 3′ ends is nonredundant, as its heart and skeletal muscle‐specific loss in mice causes profound cardiomyopathy and premature death at 3 weeks of age (Siira *et al*, 2018). In the nucleus, ELAC2 cleaves the 3′ trailers of tRNAs (Siira *et al*, 2018), following transcription by RNA polymerase III (Dieci *et al*, 2007) and the nuclear RNase P complex cleaves the 5′ leader sequences. In mitochondria, ELAC2 cleaves the 3′ tRNA ends to release mRNAs and rRNAs from the genome‐length polycistronic transcripts generated by the mitochondrial RNA polymerase (POLRMT) and hierarchically follows 5′ tRNA cleavage by the mitochondrial RNase P complex (Rackham *et al*, 2016; Siira *et al*, 2018; Bhatta *et al*, 2021). Nuclear tRNA processing is essential for the maturation of tRNAs and cytoplasmic protein synthesis, while mitochondrial tRNA processing is crucial for the release of all mitochondrial RNAs from polycistronic precursors and maturation of tRNAs required for translation of mitochondrial DNA‐encoded proteins, the biogenesis of the oxidative phosphorylating (OXPHOS) system and the rate of energy conversion (Siira *et al*, 2018).

Impaired nuclear or mitochondrial 3′ tRNA processing leads to loss of tRNAs or specific regulatory noncoding RNAs that causes an imbalance in RNA metabolism and protein synthesis, consequently resulting in severe pathologies, including cancer (Petros *et al*, 2005; Taft *et al*, 2010; Suzuki *et al*, 2011; Alvarez‐Cubero *et al*, 2015; Rackham *et al*, 2016). Mutations in ELAC2 cause severe cardiomyopathies and premature death in infancy as a consequence of disrupted mitochondrial tRNA cleavage that leads to reduced OXPHOS function (Haack *et al*, 2013). Many mutations have been identified in ELAC2 related to prostate cancer, some suspected to cause loss of function, but the main mutations are missense changes (Severi *et al*, 2003; Takaku *et al*, 2003). However, *in vivo* functional validation of these variants in prostate cancer development has been lacking. Furthermore, whether mitochondrial, nuclear or combined defects can have a role in predisposition to prostate cancer is not clear. Different studies have shown conflicting results about the association of *ELAC2* variants and prostate cancer risk, suggested to be due to differences in ethnicity of individuals as well as environmental and lifestyle factors (Camp & Tavtigian, 2002; Studeny *et al*, 2002; Severi *et al*, 2003; Xu *et al*, 2010; Djomkam *et al*, 2020).

To functionally investigate the contribution of *ELAC2* mutations to the risk of prostate cancer, we developed four *in vivo* mouse models and investigated their molecular and physiological characteristics. We used CRISPR‐Cas9 genome editing to introduce the Ala541Thr variant in the conserved mouse *Elac2* gene, used homologous recombination to generate a prostate‐specific deletion of *Elac2* and bred these lines onto the transgenic adenocarcinoma of mouse prostate (TRAMP) model that carries the simian virus 40 (SV40) T antigens under control of the probasin (*Pb*) promoter (Greenberg *et al*, 1994; Gingrich *et al*, 1999). We analysed the nuclear and mitochondrial transcriptomes and proteomes, miRNAs and small RNAs to show that reduced ELAC2 levels and activity predispose to prostate cancer by impairing the function of noncoding RNAs such as tRNAs, lncRNAs and miRNAs, which in turn alter immunometabolism. Our work provides functional evidence for the role of a variant in the human population that predisposes to prostate cancer and charts the molecular defects that lead to prostate tumorigenesis.

## Results

### *Elac2* mutations lead to inflammation and predispose to prostate hyperplasia

**Figure 1:**
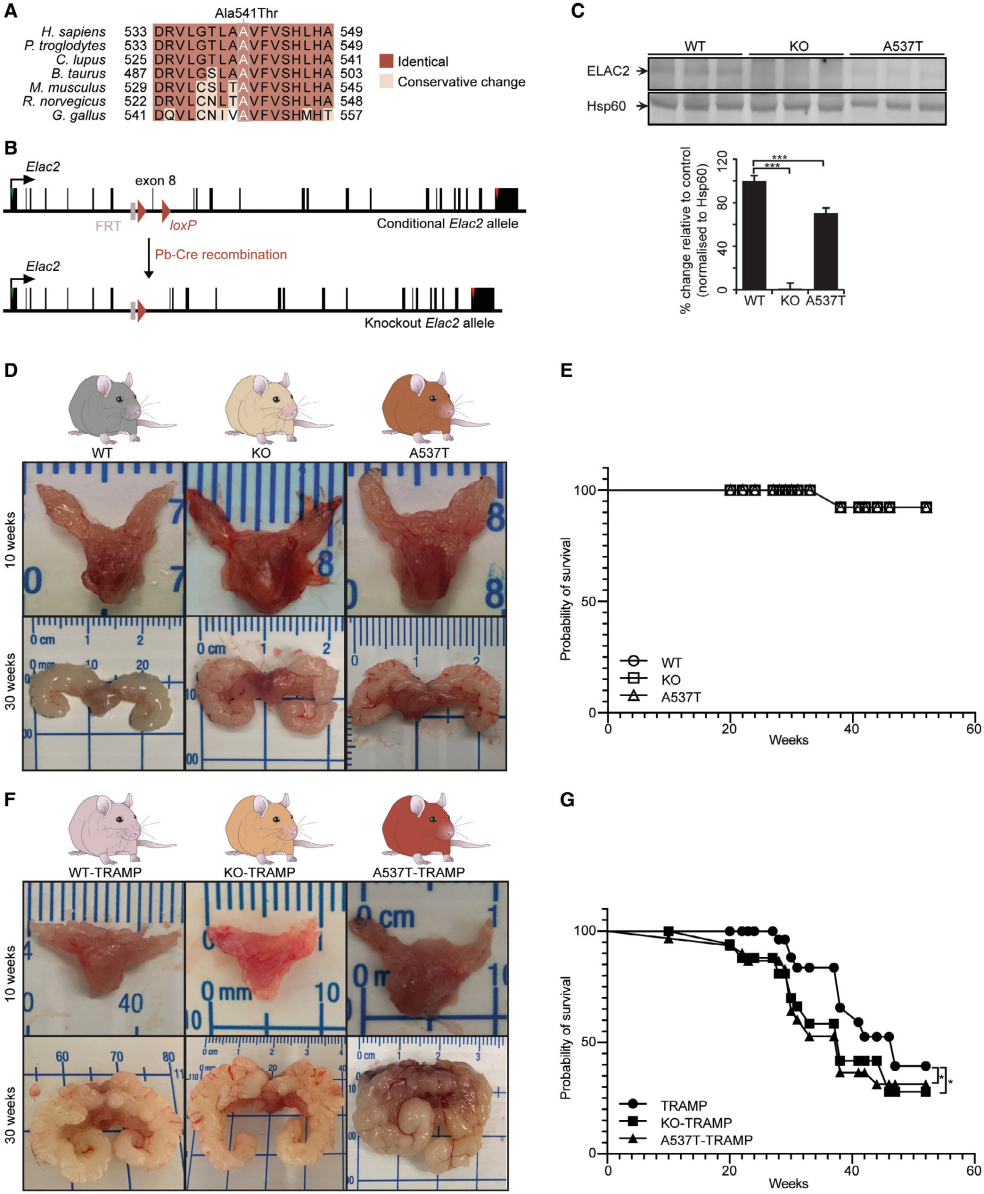
*ELAC2* mutations cause prostate enlargement and inflammation and that leads to prostate cancer on the TRAMP background Conservation of the ELAC2 protein sequence surrounding the A537T variant (white) is highlighted with residues identical to those in human sequence boxed in red.Schematic showing the homologous recombination at the *ELAC2* locus to generate prostate‐specific conditional knockout mice. LoxP sites were introduced to allow the deletion of exon 8 by *Pb*‐Cre recombinase.Immunoblot of WT, KO and A537T prostates probed with specific antibodies to detect ELAC2 and normalise its levels to Hsp60 that was used as a loading control (*n* = 3). ***P = 1.09909E‐07 for KO and ***P = 3.55091E‐05 for A537T, Student's two‐way *t*‐test, values are means ± SD of biological replicates.
*ELAC2* WT, KO and A537T prostates from 10‐ and 30‐week‐old mice. Bladder and seminal vesicles were removed in the 10‐week‐old mice. Images show the typical differences between WT, KO and A537T prostates of at least 10 different mice per genotype.Survival data for *ELAC2* WT (*n* = 28), KO (*n* = 28) and A537T (*n* = 28) mice.ELAC2 WT‐TRAMP, KO‐TRAMP and A537T‐TRAMP prostates from 10‐ to 30‐week‐old mice. Bladder and seminal vesicles were removed in 10‐week‐old mice. Images show the typical differences between WT, KO and A537T prostates of at least 10 different mice per genotype.Survival data for *ELAC2* WT‐TRAMP (*n* = 37), KO‐TRAMP (*n* = 37) and A537T‐TRAMP (*n* = 37) mice (biological replicates). Gehan–Breslow–Wilcoxon test showed significant difference (*P = 0.045) between the WT‐TRAMP and KO‐TRAMP or A537T‐TRAMP survival curves. Source data are available online for this figure.

The most common missense mutations in *ELAC2* associated with prostate cancer predisposition are Ser217Leu and Ala541Thr substitutions. The Ser217Leu variant is located in the hydrophilic segment of the protein, resulting in a substitution of the hydrophobic leucine residue and possibly leads to an alteration of the protein structure (Tavtigian *et al*, 2001). In contrast, the *ELAC2* Ala541Thr variant is in a region that is highly conserved across eukaryotic species (Fig 1A) and may impair protein function by interacting with adjacent histidine motifs (Tavtigian *et al*, 2001; Korver *et al*, 2003). Approximately 6% of the Caucasian population possess the Ala541Thr variant (Vesprini *et al*, 2001), and genetic and genome‐wide association studies (GWAS) have predicted that this variant can increase the risk of prostate cancer (Tavtigian *et al*, 2001); however, functional and physiological validation for the effects of *ELAC2* variants on prostate cancer and enzyme function is lacking.

To examine the role of *Elac2* mutations in prostate function and tumorigenesis, we knocked out the *ELAC2* gene in the prostate only using a prostate‐specific Cre recombination to excise exon 8. In addition, we used CRISPR‐Cas9 technology to introduce the threonine variant into the *ELAC2* gene at amino acid position 537 (equivalent to the human Ala541Thr variant) in the mouse genome (Fig 1B). We bred these mice to produce either homozygous prostate‐specific *Elac2* knockout mice (referred to as KO) or full‐body homozygous Ala537Thr variant mice (referred to as A537T), along with their respective homozygous control mice (referred to as WT), and their genotypes were confirmed by Sanger sequencing (Appendix Fig S1A and B). Prostate‐specific deletion of *Elac2* resulted in loss of ELAC2 in the KO mouse prostates, and the Ala537Thr variant reduced the stability of ELAC2 in the A537T mouse prostates (Fig 1C). We examined the prostates of these mice at 10 and 30 weeks of age and identified consistent inflammation and enlargement of the prostates with hyperplasia of the dorsal and anterior lobes in the 30‐week‐old A537T mice (Fig 1D); however, the life spans of the KO and A537T mice were comparable to WT mice (Fig 1E). Analyses of blood profiles revealed increases in white blood cells, neutrophils and lymphocytes in the KO mice compared with controls at 10 weeks of age (Appendix Fig S1C), likely as a consequence of inflammation.

To investigate whether *Elac2* mutations can predispose to prostate tumorigenesis and model a “two‐hit hypothesis” tumorigenesis scenario, we used TRAMP mice where the *Pb* promoter expresses SV40 large‐T and small‐t antigens in the prostatic epithelium (Greenberg *et al*, 1994) to initiate progressive prostatic neoplasia with hyperplasia by 10 weeks of age and adenocarcinoma from 18 to 30 weeks of age (Gingrich *et al*, 1999). We bred the A537T and KO mice with TRAMP mice to generate KO‐TRAMP and A537T‐TRAMP mice and used the WT‐TRAMP in addition to the WT mice as controls. The KO‐TRAMP and A537T‐TRAMP mice had enlarged prostates and developed consistent prostatic intraepithelial neoplasia (PIN) by 10 weeks of age compared with the WT‐TRAMP mice that had sporadic appearance of PIN (Fig 1F). PIN was found in the three strains on the TRAMP background by 30 weeks of age; however, the A537T‐TRAMP mice had the largest prostates and engorged seminal vesicles with substantial tumour formation, followed by a similar phenotype in the KO‐TRAMP compared with the WT‐TRAMP control mice (Fig 1F). However, there were no differences in the blood profiles of the different strains at different ages (Appendix Fig S1D), suggesting that blood composition is not a reliable indicator of prostate tumorigenesis. Survival data revealed that higher numbers of A537T‐TRAMP and KO‐TRAMP mice could not survive by 30 weeks of age and beyond 30 weeks a greater number of these mice die as a consequence of their tumours compared with the WT‐TRAMP mice (Fig 1G), indicating that the *Elac2* A537T variant can predispose and exacerbate prostate cancer.

**Figure 2:**
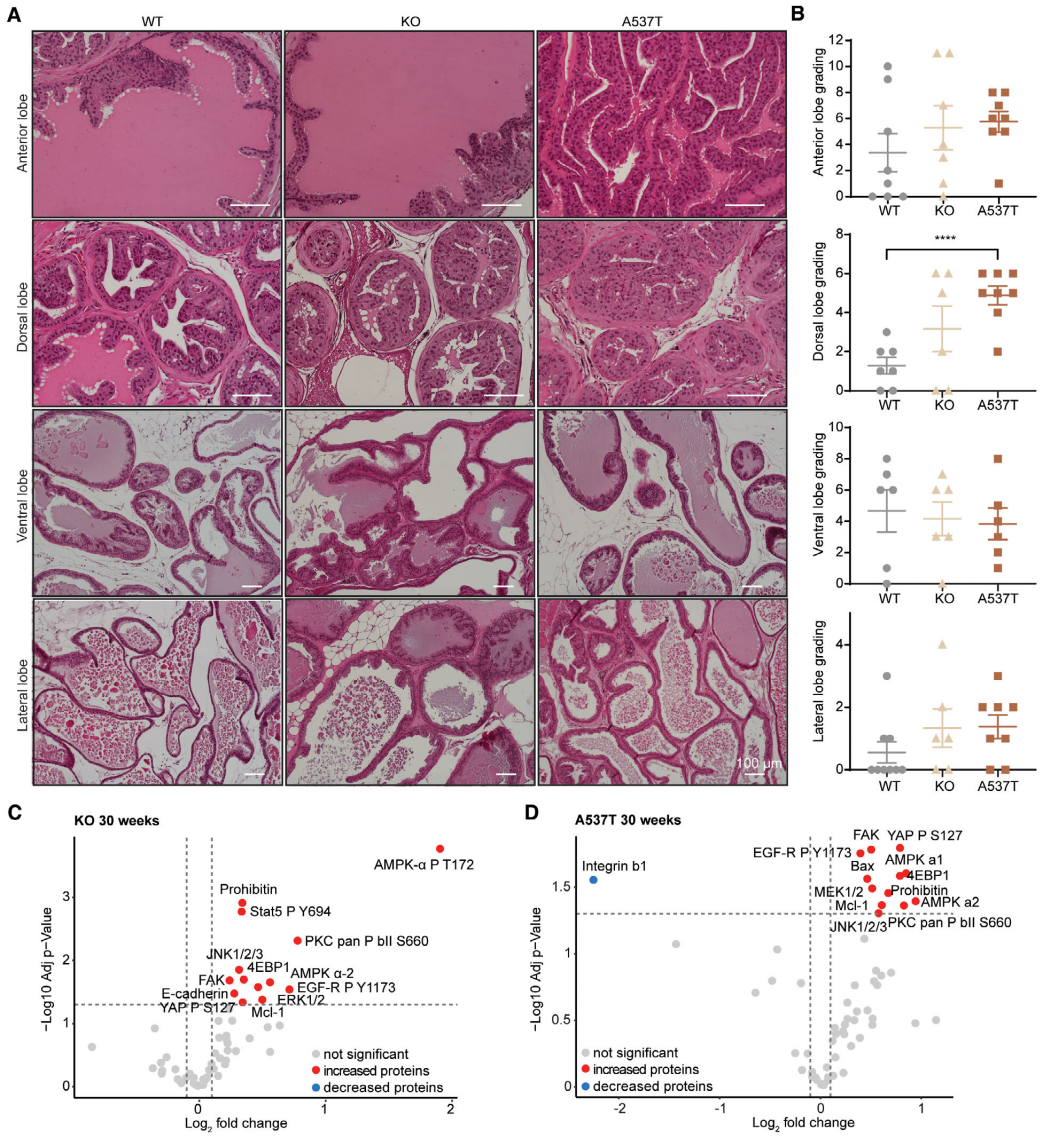
*ELAC2* A537T variant leads to inflammation, cell proliferation and differentiation in the prostate Representative images of anterior, dorsal, ventral and lateral lobe sections from 30‐week‐old *ELAC2* WT (*n* = 7), KO (*n* = 7) and A537T (*n* = 8) mice. 5 μm sections of each lobe were cut and stained with haematoxylin and eosin.Histological changes were scored according to Suttie *et al* with additional categories added to the scoring system (described in the methods; *n* = 6–8 biological replicates). Values are means ± SD of biological replicates, ****P = 0.001 compared with WT by two‐tailed Student's *t*‐test.Reverse‐phase protein array (RPPA) showing the protein changes in 30‐week‐old KO mice compared with WT mice. Volcano plots comparing Log_2_ fold changes against the −log^10^ *P*‐value of five mice for each genotype. Significantly (*P* < 0.05 and FC above 1) increased proteins are shown in red, and significantly decreased proteins are shown in blue.RPPA results for 30‐week‐old A537T mice compared with WT mice. Volcano plots comparing Log_2_ fold changes against the −log^10^ *P*‐value of five mice for each genotype. Significantly (*P* < 0.05 and FC above 1) increased proteins are shown in red, and significantly decreased proteins are shown in blue. Source data are available online for this figure.

We used haematoxylin and eosin (H&E) staining to profile and score the four prostatic lobes based on the Suttie *et al g*rading system (Suttie *et al*, 2003; Fig 2A and B, and Appendix Fig S2A and B). The ventral and lateral prostate lobes in 10‐week‐old KO and A537T mice were similar to the WT (Appendix Fig S2A), however, there was early onset hyperplasia in the anterior lobe of the 10‐week‐old KO and A537T mice, which displayed excessive branching into the lumen in the prostates of the KO mice and increased cell growth and cell piling in the prostates of the A537T mice (Appendix Fig S2A). The dorsal lobe in the 10‐week‐old KO mice was comparable to the WT dorsal lobe; however, this lobe was not formed properly in the A537T mice and showed stromal thickening (Appendix Fig S2A), consistent with the significantly higher grading of the dorsal lobe in the A537T mice (Appendix Fig S2B). By 30 weeks, the phenotype of the anterior and dorsal lobe worsened in both A537T and KO mice. The anterior lobe showed disarrayed nuclei, mild hyperplasia and branching into the lumen in both mutant strains compared with controls (Fig 2A). In addition, the anterior lobe in the A537T mice showed grade 2 lesions, while the KO mice displayed increased stroma thickening (Fig 2A). Similar to the 10‐week‐old prostates, we observed significant changes in the dorsal lobe of 30‐week‐old A537T mice (Fig 2B), with papillary structures protruding into the lumen, in addition to grade 2 focal lesions, cell crowding and mild hyperplasia (Fig 2A). The lateral and ventral lobes were comparable to the respective lobes in the WT mice without major changes; however, mild focal to multifocal flat lesions and inflammatory infiltration were visible (Fig 2A).

The identified histological changes in cell proliferation, hyperplasia and inflammation of the prostates were examined at a molecular level using reverse‐phase protein arrays (RPPA) for protein markers of cell proliferation, tumorigenesis and inflammation. Proteins implicated in prostate tumorigenesis such as protein kinase C (PKC) pan P βII (Kikkawa *et al*, 1983; Griner & Kazanietz, 2007), E‐cadherin (Navarro *et al*, 1991; Perl *et al*, 1998; Padmanaban *et al*, 2019; Na *et al*, 2020) and eukaryotic translation initiation factor 4E‐binding protein 1 (4EBP1; Kremer *et al*, 2006; Hsieh *et al*, 2015; Musa *et al*, 2016) were increased in 10‐week‐old KO and A537T mice (Appendix Fig S2C and D). While in the prostates of the A537T mice, the extracellular signal‐regulated kinase 1/2 (ERK1/2) was also increased (Appendix Fig S2D), which has been implicated in prostate tumorigenesis (Gioeli *et al*, 1999; Kinkade *et al*, 2008; Yeh *et al*, 2019; Schmidt *et al*, 2021). These proteins remained elevated in the prostates of the 30‐week‐old KO and A537T mice along with additional proteins involved in cell proliferation, differentiation and survival, such as the focal adhesion kinase (FAK), myeloid‐cell leukaemia 1 (MCL‐1), epidermal growth factor receptor (EGFR) and mitogen‐activated protein kinase (MEK1/2) and biomarkers of inflammation such as AMP‐activated protein kinase (AMPK) α‐1/2 and c‐Jun N‐terminal kinase 1/2/3 (JNK1/2/3; Fig 2C and D), consistent with the increased proliferation, enlargement and inflammation of the prostates identified by histology (Figs 1C, and 2A and B, and Appendix Fig S2A and B).

### *Elac2* mutations cause prostate cancer on the TRAMP background

**Figure 3:**
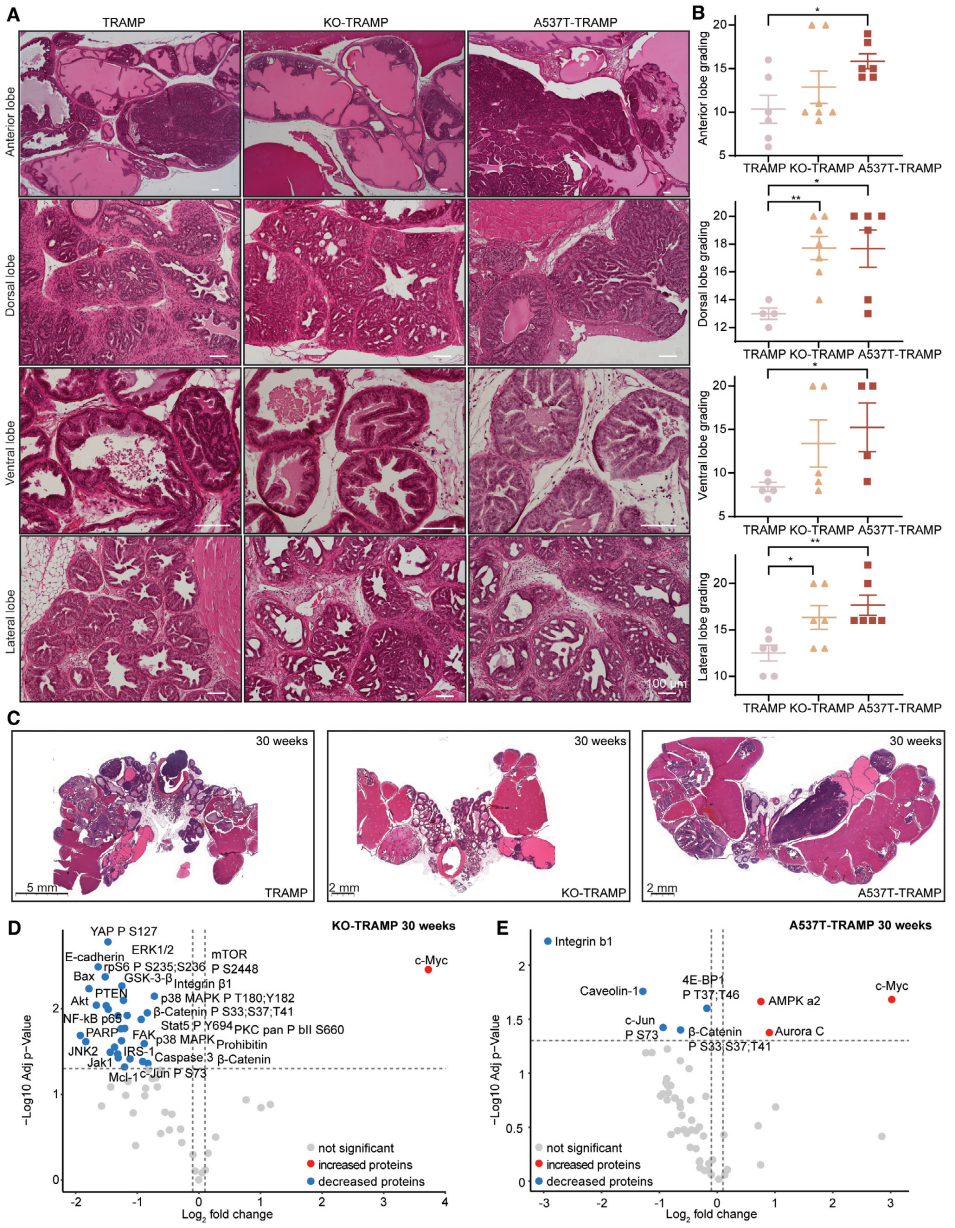
*ELAC2* A537T variant on the TRAMP background leads to the development of adenocarcinomas in the prostate Representative images of anterior, dorsal, ventral and lateral lobe sections from 30‐week‐old WT‐TRAMP (*n* = 5), KO‐TRAMP (*n* = 5) and A537T‐TRAMP mice (*n* = 5). 5 μm sections of each lobe were cut and stained with haematoxylin and eosin.Histological changes scored according to Suttie *et al* with additional categories (*n* = 6–7 biological replicates); values are means ± SD of biological replicates. Anterior lobe *P = 0.013, Dorsal lobe **P = 0.003, *P = 0.025, Ventral lobe *P = 0.031 and Lateral lobe **P = 0.0038, *P = 0.032, compared with WT‐TRAMP by two‐tailed Student's *t*‐testing.Representative histological images of complete prostate sections from 30‐week‐old WT‐TRAMP, KO‐TRAMP and A537T‐TRAMP showing the prominent tumours (black arrows) in the mutant strains compared with the WT‐TRAMP mice. Images show the typical differences between WT‐TRAMP, KO‐TRAMP and A537T‐TRAMP prostates of at least 10 different mice per genotype.Reverse‐phase protein array (RPPA) results for 30‐week‐old KO‐TRAMP mice (*n* = 5) compared with WT‐TRAMP mice (*n* = 5). Volcano plots comparing Log_2_ fold changes against the −log^10^ *P*‐value of five mice for each genotype. Significantly (*P* < 0.05 and FC above 1) increased proteins are shown in red, and significantly decreased proteins are shown in blue.RPPA results for 30‐week‐old A537T‐TRAMP mice (*n* = 4) compared with WT‐TRAMP mice (*n* = 3). Volcano plots comparing Log_2_ fold changes against the −log^10^ *P*‐value of five mice for each genotype. Significantly (*P* < 0.05 and FC above 1) increased proteins are shown in red, and significantly decreased proteins are shown in blue. Source data are available online for this figure.

We investigated and graded the prostate lobes of the KO‐TRAMP and A537T‐TRAMP compared with the WT‐TRAMP mice to determine whether mutation of *Elac2* can potentiate or exacerbate prostate cancer (Fig 3A and B, and Appendix Fig S3A and B). We observed at 10 weeks of age hyperplasia and low‐grade adenoma in the dorsal lobe of all three mutant lines (Appendix Fig S3A and B). The TRAMP mutation promotes tumorigenesis predominantly in the dorsal lobe, as shown in Suttie *et al* (2003), where the highest grading was 5 in the dorsal lobe, and we found the A537T mutation and KO‐TRAMP exacerbated tumorigenesis. The lateral prostate lobes from all mutant mice at 10 weeks were highly proliferative with grade 3 or grade 4 lesions displaying characteristic multifocal papillary cell crowding protruding into the lumen (Appendix Fig S3A and B). In contrast, we observed in the ventral lobe of the A537T‐TRAMP mice a range of diverse lesions (from grade 1 to grade 3) ranging from mild hyperplasia to multifocal papillary projections into the lumen, while in the lateral ventral lobe of the KO‐TRAMP mice we identified mostly advanced grade 3 and 4 lesions, consistent with papillary projections protruding into the lumen and proliferating cells (Appendix Fig S3A and B). The anterior lobe showed mild multifocal hyperplasia and cribriform lesions without tumour formation and low‐grade lesions in all three mutant lines. There were comparable prostate lesions in the KO‐TRAMP and A537T‐TRAMP mice to the WT‐TRAMP mice, albeit in some lobes this was more severe when *Elac2* was mutated, suggesting that *Elac2* contributes to prostate tumorigenesis (Appendix Fig S3C).

By 30 weeks, all lobes in the A537T‐TRAMP mice showed significantly greater lesions and defects compared with WT‐TRAMP mice (Fig 3A and B). The anterior lobe of the A537T‐TRAMP prostates showed grade 5 and grade 6 lesions that were highly proliferative and the lumen was drastically expanded and filled with tumorous mass. Furthermore, adenomas were visible, and, in some samples, the lobe was not discernible due to tumour overgrowth. The A537T variant exacerbated the effects on the dorsal lobe on the TRAMP background strain, resulting in the development of adenocarcinomas across the entire lobe (Fig 3A). The lateral and ventral lobes of the A537T‐TRAMP prostates had tumour invasions with grade 4 lesions, and the majority of these lobes were indiscernible due to neoplastic glands. The anterior lobes of the 30‐week‐old KO‐TRAMP mice had grade 3 and grade 4 lesions and hyperplasia that were similar to the WT‐TRAMP lobes, but the ventral lobes in KO‐TRAMP had grade 3 lesions similar to lobes in the A537T‐TRAMP mice (Fig 3A and B). However, the dorsal and lateral prostate lobes of the 30‐week‐old KO‐TRAMP mice had significantly increased grade 3 and grade 4 lesions, proliferative epithelium and tumour development that were comparable to the A537T‐TRAMP prostates (Fig 3B). Increased tumour mass in the KO‐TRAMP and A537T‐TRAMP compared with the WT‐TRAMP prostates is further apparent in Fig 3C, showing that the *Elac2* mutations exacerbate prostate tumorigenesis. RPPA analyses supported the histological findings revealing increases in protein markers of tumorigenesis onset at 10 weeks of age (including FAK, EGFR and JNK2) and most remarkably cancer progression at 30 weeks of age with the greatest and consistent increases in MYC and concomitant reduction in PTEN levels in the KO‐TRAMP and A537T‐TRAMP mice, compared with WT‐TRAMP mice (Fig 3D and E, and Appendix Fig S3D and E; Miller *et al*, 2012; Yi *et al*, 2019; Rebello *et al*, 2021). These findings reveal that the KO‐TRAMP and A537T‐TRAMP mice can be used as effective and physiologically consistent models of prostate cancer.

### ELAC2 is required for mitochondrial and nuclear RNA processing in the prostate

**Figure 4:**
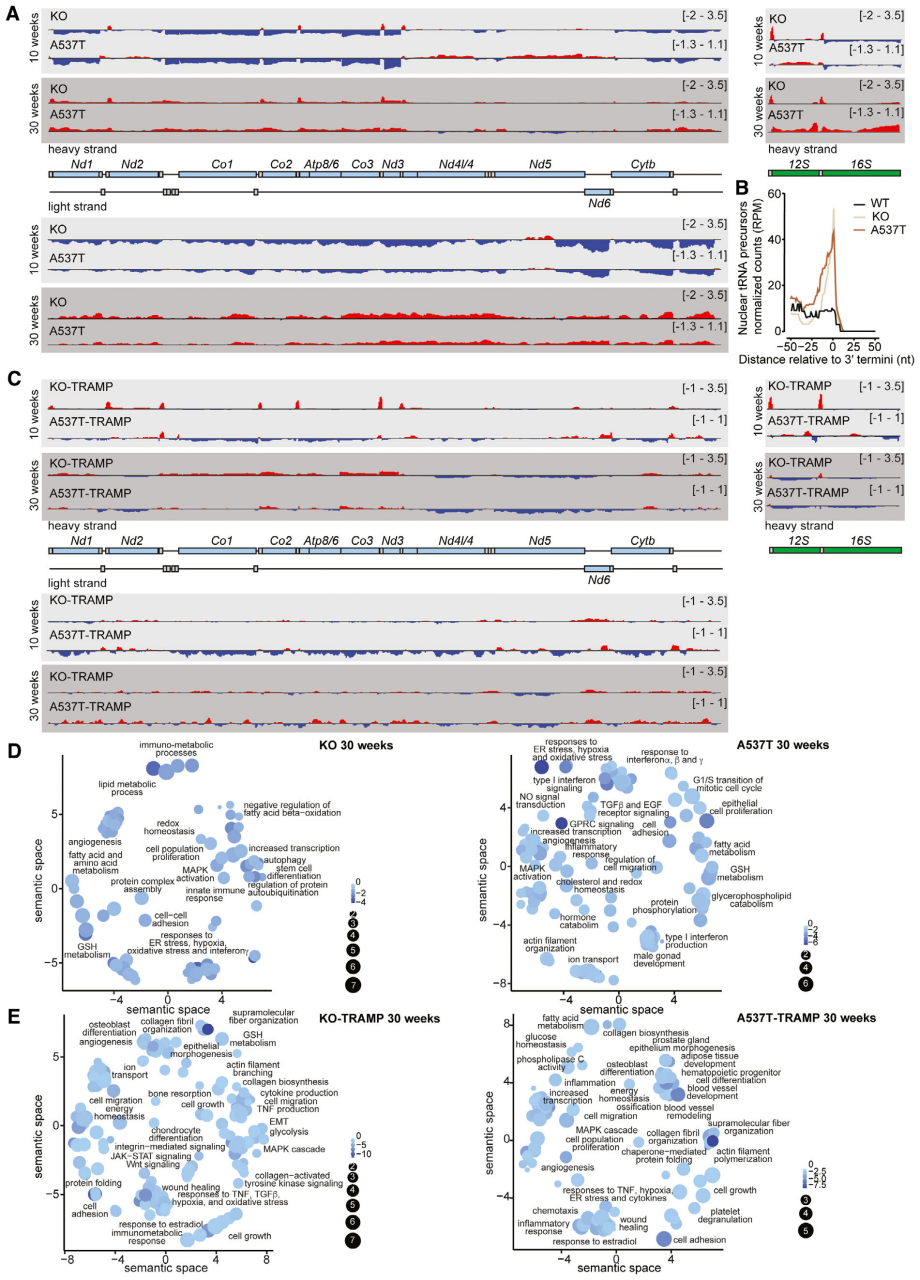
*Elac2* A537T variant and *Elac2* deletion impair tRNA processing leading to transcriptome‐wide changes that promote prostate tumorigenesis Transcriptome analysis of mitochondrial transcripts by RNA‐Seq in prostates from 10‐ and 30‐week‐old mice.
AComplete maps of the changes in mitochondrial transcript abundance determined by mean RNA‐Seq coverage from KO and A537T mice (*n* = 3 of each line; relative to WT controls) on heavy (first four tracks) and light (bottom four tracks) strands of the mtDNA. Increases are shown in red, and decreases are shown in blue (log_2_ [RPMKO/RPMWT] or log_2_ [RPMA537T/RPMWT]; scale ranges, −2 to 3.5). The mitochondrial genome is displayed in the centre; mRNAs are in blue, tRNAs are in grey, and rRNAs are in green.BFrequency distribution of prostate nuclear precursor tRNA reads isolated from control, KO and A537T mice (*n* = 3 of each genotype). Windows are centred on reads that align 50 nt away from either side of all annotated 3′ ends of nuclear tRNAs marked as 0.CGenome browser view of the mean RNA‐Seq coverage of the KO‐TRAMP (log_2_[RPMKO‐TRAMP/RPMWT‐TRAMP]) or A537T‐TRAMP (log_2_[RPMA537T‐TRAMP/RPMWT‐TRAMP]) mice; scale ranges, −1 to 3.5.D, EGene ontology changes in gene expression show the significantly changing biological processes in 30‐week‐old (D) KO and A537T or (E) KO‐TRAMP and A537T‐TRAMP mice compared with their respective controls. The colour scale represents fold change (FC) for each pathway, and set size shows the number of genes within each pathway.

To investigate the effects of the *Elac2* mutations on nuclear and mitochondrial tRNA processing in the prostate and their contribution to its pathology, we carried out transcriptomic analyses of prostate RNA isolated from all four mutant lines and their respective control mice (Fig 4 and Dataset EV1). ELAC2 plays an essential and nonredundant role in the cleavage of mitochondrial tRNA 3′ ends to release all mitochondrial RNA transcripts, and consequently, loss of ELAC2 leads to the accumulation of precursor tRNA‐containing transcripts (Siira *et al*, 2018). We show that loss of ELAC2 in the prostates of the KO mice causes mitochondrial transcriptome‐wide accumulation of tRNA‐containing precursors (Fig 4A). Reduced ELAC2 activity and impaired tRNA processing was also found in the A537T mice (Fig 4A), consistent with the reduced ELAC2 levels caused by the variant. Furthermore, loss of ELAC2 in the prostates of the KO mice and reduced levels of ELAC2 in the prostates of the A537 mutant mice caused increased accumulation of 3′ unprocessed nuclear tRNAs (Fig 4B and Appendix Fig S4).

Although the loss or reduction of ELAC2 cleavage as a consequence of its deletion or mutation results in loss of mitochondrial mRNAs at 10 weeks, transcript levels were increased in both the KO and A537T prostates by 30 weeks (Fig 4A), likely as a consequence of the identified hyperplasia and cell proliferation in the mutant lines compared with controls (Fig 2A and B) and increased mitochondrial transcription that is a compensatory response to persistent loss of mature mitochondrial RNAs (Perks *et al*, 2018). Similar mitochondrial tRNA processing defects and trends were present in the KO‐TRAMP and A537T‐TRAMP mice compared with WT‐TRAMP mice, where the loss of ELAC2 increased the level of tRNA precursors compared with the A537T mice (Fig 4C). These findings show that the ELAC2 A537T variant impairs mitochondrial and nuclear tRNA processing in the prostate that may be a contributing factor to tumorigenesis.

### ELAC2 mutations impair tRNA processing causing transcriptional activation of innate stress response pathways that initiate prostate tumorigenesis

Transcriptome‐wide differential gene expression analyses were used to determine the effects of ELAC2 loss or reduction on nuclear transcripts (Fig 4D, and Datasets EV1 and EV2). The most notable changes in the prostate transcriptomes of 10‐week‐old KO and A537T mice compared with controls were in mitochondrial structure and function, including OXPHOS and protein folding, followed by responses to ER and oxidative stress and changes in epithelial cell adhesion and proliferation (Appendix Fig S5A and B), consistent with impaired RNA processing that has been shown to alter mitochondrial energy metabolism (Haack *et al*, 2013; Metodiev *et al*, 2016; Rackham *et al*, 2016; Siira *et al*, 2018). In addition, reduced 3′ processing in the A537T mouse prostates contributed to the activation of genes involved in prostate epithelium morphogenesis (Appendix Fig S5A). The reduced ELAC2 activity in the KO and A537T mice by 30 weeks of age, in addition to altered energy metabolism and consistent stress responses, led to transcriptional activation of epithelial cell proliferation, angiogenesis and immunometabolic pathways, including interferon signalling (Fig 4D), consistent with the inflammation and increased proliferation of the prostates identified by histology compared with control mice. The transcriptomic changes in the prostates from the KO‐TRAMP and A537T‐TRAMP mice compared with the WT‐TRAMP mice at 10 weeks were in stress response pathways to hypoxia, and networks that supported increased cell proliferation, migration and extracellular matrix organisation, in addition to angiogenesis and protein folding changes that cumulatively contributed to PIN formation and the onset of prostate tumorigenesis (Appendix Fig S5B). A significantly greater number of pathways that potentiate prostate tumorigenesis and cancer were altered in the 30‐week‐old KO‐TRAMP and A537T‐TRAMP compared with the WT‐TRAMP mice (Fig 4D and E), which were reflective of the morphological changes observed in their prostates. These included increases in cell proliferation, migration, immunometabolic and inflammatory pathways, such as MAPK activation, JAK–STAT and WNT signalling, and responses to ER and hypoxia stress (Fig 4D and E, and Dataset EV2) stimulated by impaired protein homeostasis and OXPHOS defects. The transcriptional changes in the four different mutant strains compared with their respective controls were consistent between each other indicating that the changes were driven by reduced ELAC2 function and were a direct consequence of impaired tRNA processing.

### Defects in aerobic metabolism drive prostate tumorigenesis via the PERK‐mediated unfolded protein response

**Figure 5:**
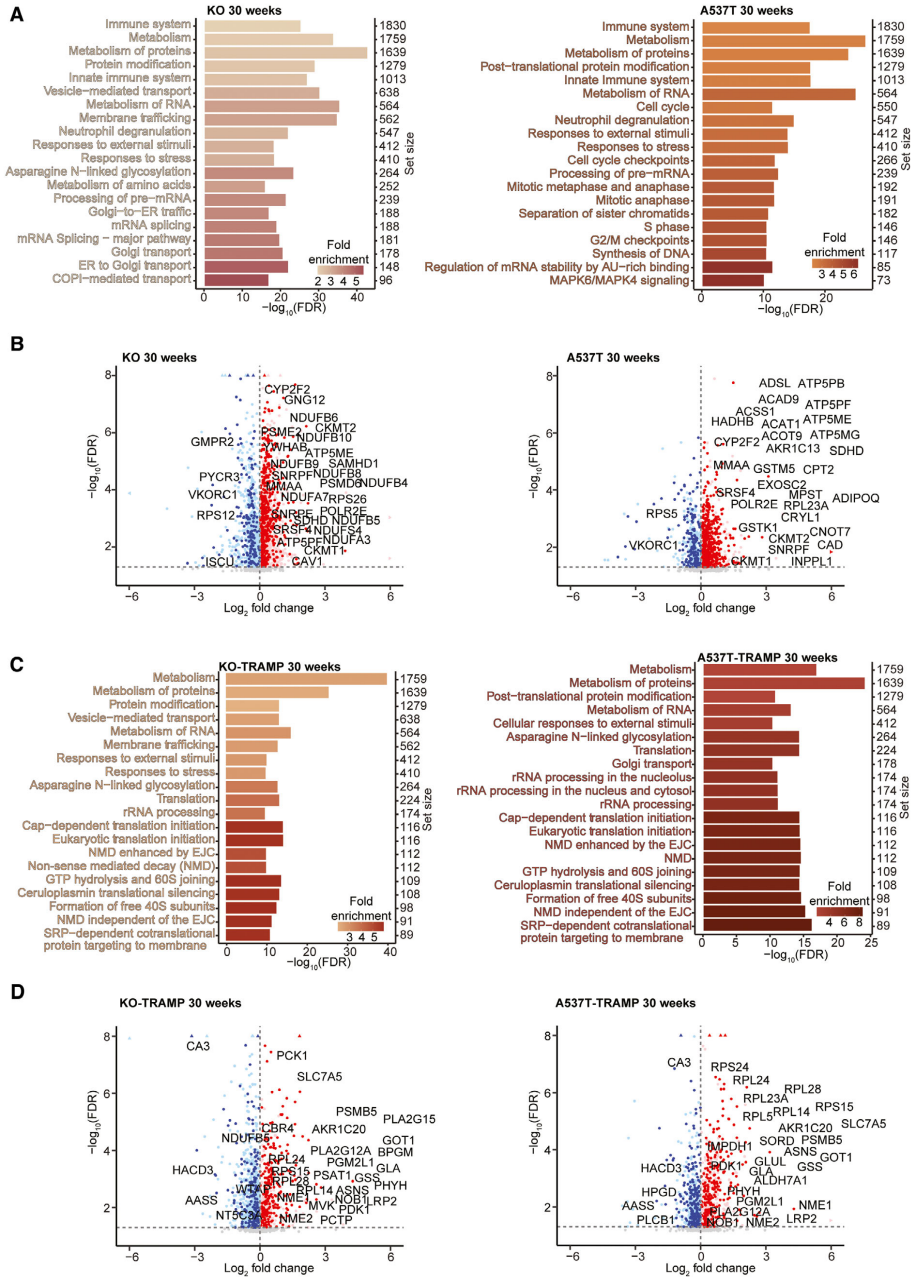
Proteome changes identify common processes in the mutant lines that reflect their tumorigenic profiles A–DProteomic changes in prostates that are in common in the KO (*n* = 5 biological replicates) and A537T (*n* = 5 biological replicates) mice compared with WT control mice (*n* = 5 biological replicates) at 30 weeks based on the Reactome pathway determined using PANTHER. (A) Specific protein changes that were in common between the KO and A537T lines at 30 weeks are shown in volcano plots; (B) increased proteins are shown in red and reduced proteins are shown in blue. The top 20 most significant pathways that are based on proteome changes in common between KO‐TRAMP (*n* = 5 biological replicates) and A537T‐TRAMP (*n* = 5 biological replicates) mice relative to WT‐TRAMP (*n* = 5) mice at 30 weeks (C) and specific protein changes (D). Data information: In panels (A and C), the intersect results show the top 20 pathways with the highest −log10 (FDR) for each line, relative to the respective control, that are also identified as significant in the corresponding mouse line. The colour scale represents fold change (FC) for each pathway, and set size is the number of genes within each pathway.

Next, we performed proteomic analyses on prostates from the four mutant and two control mouse lines to identify global changes that were a consequence of impaired tRNA processing and led to prostate tumorigenesis and cancer (Fig 5, Appendix Figs S6–S8, Datasets EV3 and EV4). The greatest number of protein changes in the prostates of all four mutant lines at both 10 weeks (Appendix Figs S6–S8 and Dataset EV3) and 30 weeks (Fig 5A and B, Appendix Fig S7B and Dataset EV3) were in metabolic pathways, particularly those involving mitochondrial proteins associated with energy conversion, lipid membranes, transport and cell death, highlighting the requirement of ELAC2 for mitochondrial function. In addition, reduced 3′ processing contributed to significant defects in protein networks involving nuclear RNA splicing, translation initiation, protein synthesis and RNA decay in the prostates of the mutant mice (Fig 5A and B), consistent with the role of ELAC2 in nuclear tRNA cleavage (Siira *et al*, 2018). The changes in the 10‐week‐old mutant mice reflected defects in mitochondrial and nuclear tRNA processing (Appendix Figs S6A, S7A, and S8A and B), and in the 30‐week‐old mutant mice, these changes were accompanied by alterations in cell cycle, immune and stress responses and increased MAPK signalling (Fig 5A and B, and Appendix Figs S7B and S9A–D) that supported the observed transcriptome changes (Fig 4D) and prostate dysfunction (Fig 2A and B). The differences between the prostate proteomes of the KO and A537T mice revealed that the A537T variant caused greater representation of pathways and processes involved in tumorigenesis and inflammation (Appendix Fig S7A and B), which was even more apparent by 30 weeks in the A537T mice (Appendix Fig S7B), including ERK and Forkhead box class O (FOXO) activation, known to drive prostate cancer epithelial‐mesenchymal transition (Liu *et al*, 2015b; Shin *et al*, 2019; Yan & Huang, 2019), supporting our findings that *Elac2* mutations can predispose to the activation of tumorigenesis pathways.

In the 10‐week‐old KO‐TRAMP and A537T‐TRAMP mice compared with the WT‐TRAMP mice, proapoptotic proteins such as AIFM3 were reduced, while antiapoptotic proteins, such as TRIAP1, and metabolic and immune response proteins (AKR1B7, ENO3 and TCIRG1) were increased (Appendix Fig S8D–F), which was consistent with cell growth, tumorigenesis and altered immunometabolism in the prostates (Fig 5C and D). In 30‐week‐old KO‐TRAMP and A537T‐TRAMP mice compared with the WT‐TRAMP mice, there were greater increases in proteins involved in mitochondrial metabolism and OXPHOS function (Appendix Fig S9E–H). This is a common compensatory response to prolonged impairment in mitochondrial gene expression (Perks *et al*, 2018), and consequent OXPHOS defects that result in increased anaerobic metabolism and stimulate tumorigenesis and angiogenesis (Herkenne *et al*, 2020; Lee *et al*, 2020). Interestingly, we found a consistent increase in the SRA stem‐loop interacting RNA‐binding protein (SLIRP), in the four mutant lines compared with their respective controls at 10 and 30 weeks of age (Appendix Figs S8 and S9), suggesting that components of the mitochondrial gene expression machinery are increased in an effort to mitigate the reduction in protein synthesis due to impaired RNA metabolism. There was a greater perturbation in the proteomes of the mutant strains from 10 to 30 weeks of age that was in line with tumorigenesis (Appendix Fig S9C and D) and prostate cancer (Appendix Fig S9G and H) in the KO‐TRAMP and A537T‐TRAMP mice (Fig 3A and B). This was further reflected in the global changes in translation, RNA metabolism (splicing, stability and processing), stress responses and transport (Fig 5C) that were in common between the 10‐week‐old KO‐TRAMP and A537T‐TRAMP mice and further exacerbation of immunometabolism that accompanied the development of prostate cancer by 30 weeks of age in these mice compared with the WT‐TRAMP control mice (Fig 5D). The main differences between the 30‐week‐old KO and A537T mice independent of the TRAMP background were predominately the activation of transport and RNA processing pathways in KO/KO‐TRAMP mice and an upregulation in cell cycle and cell growth processes via WNT signalling and IGF transport and uptake in A537T/A537‐TRAMP mice, reflective of prostate tumorigenesis and cancer progression compared with prostate enlargement in the KO and A537T mice (Appendix Fig S7B and D).

**Figure 6:**
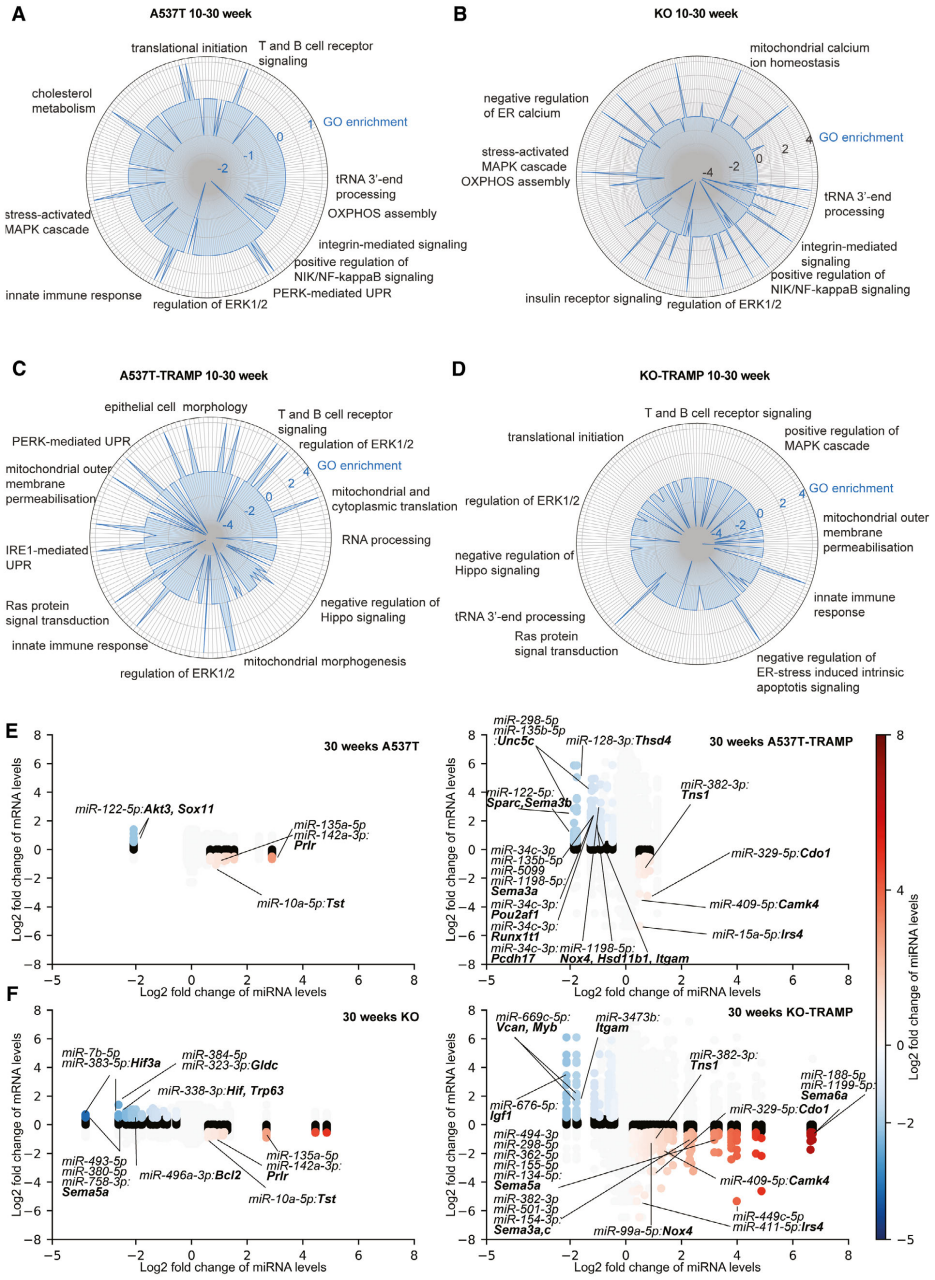
miRNA imbalance potentiates tumorigenesis in the prostates of the A537T‐TRAMP mice A–DRadar plots showing prostate‐specific gene ontologies related to prostate cancer development (Fan *et al*, 2018; Lu & Ding, 2019; Liu *et al*, 2020) in the proteomes over time in (A) A537T (*n* = 5) or (B) KO (*n* = 5) mice compared with WT control mice (*n* = 5) from 10 to 30 weeks and in (C) A537T‐TRAMP (*n* = 5) and (D) KO‐TRAMP (*n* = 5) mice relative to WT‐TRAMP (*n* = 5) mice from 10 to 30 weeks, all were biological replicates. Negative numbers represent loss of gene ontology compared with their respective controls, positive numbers represent gain of gene ontology, and 0 represents no change.E, FDot plots showing expression change of mRNA/miRNA pairs in the 30‐week mutant lines compared with their respective controls. mRNA log_2_ fold change compared with controls is on the *y*‐axis, while the colour and *x*‐axis denote the log_2_ fold change observed in the miRNA compared with controls.

Next, we analysed changes in proteins associated with cancer development over time by comparing the changes in 10‐ and 30‐week‐old mice of each line relative to their respective controls. In the KO and A537T mice, an increase in processes involved in tRNA processing, impaired mitochondrial function (OXPHOS assembly, ion homeostasis), stress and innate immune responses (such as the activation of PERK‐mediated unfolded protein response (UPR) and the MAPK cascade) and inflammation (NIK/NF‐kappaB signalling) were the most prominent changes (Fig 6A and B). This indicates that defects in RNA metabolism can stimulate the innate immune responses via the PERK‐mediated UPR pathway that contributes to inflammation and increased cell growth in the prostates of the mutant mice. The same mutations on the TRAMP background, as a secondary oncogenic insult, exacerbated the stress responses through PERK‐ and IRE1‐mediated UPR and pathways, promoting cell growth and differentiation (including ERK1/2 and RAS signalling, Fig 6C and D) that initiated and promoted prostate cancer in the mutant strains compared with the WT‐TRAMP mice. These underlying molecular drivers of prostate tumorigenesis in the *Elac2* mutant mice provide functional evidence that *ELAC2* mutations can predispose to prostate cancer.

### ELAC2 is a rheostat that regulates miRNA homeostasis

Loss of ELAC2 has been shown to impair nuclear tRNA processing, leading to an imbalance in regulatory noncoding RNAs that contributes to disease (Siira *et al*, 2018). MiRNA sequencing of mutant and control mouse prostates from 10‐ and 30‐week‐old mice identified specific changes in the miRNA pool (Appendix Fig S10A–D and Dataset EV5) that targeted common oncogenic and tumour suppressor mRNAs in the mutant strains relative to their respective controls (Fig 6E and F, and Appendix Fig S10A). The imbalance in the miRNA levels reflects previous findings where loss of ELAC2 in the heart resulted in substantial restructuring of the miRNA pool, including both reduction and increase of specific miRNAs (Siira *et al*, 2018), due to alterations in the processing of precursor RNAs from tRNA genes or expression of mRNA genes with intragenic miRNAs. A pairwise correlation analysis between perturbed miRNAs and their mRNA targets suggested altered pathways in the gene regulatory circuitry upon mutation of *Elac2* in the prostate. Elevated miRNAs as a direct or indirect consequence of impaired tRNA processing correlated with reduced mRNAs involved in mitochondrial function and potentiation of inflammation and cell growth in the KO and A537T mutant mice compared with controls (Appendix Fig S10A), such as miR‐10a‐5p that correlated with reduced the expression of the mitochondrial thiosulfate sulphur transferase (*Tst*; Fig 6E and F). Reduced miRNAs such as miR‐122‐5p and miR‐383‐5p/miR‐338‐3p correlated with an increase of mRNAs involved in cell transformation (*Gldc*; Liu *et al*, 2021), inhibition of apoptosis (*Bcl2*), prostate tumorigenesis (*Trp63*; Pignon *et al*, 2013) and hypoxia (*Hif3a*; Peng *et al*, 2022) in the KO and A537T mutant mice compared with controls (Fig 6E and F).

In the KO‐TRAMP and A537T‐TRAMP mice, elevated miRNAs resulted in reduction of prognostic markers of prostate cancer and metastasis, such as cysteine dioxygenase 1 (*Cdo1*; Meller *et al*, 2016) and tensin 1 (*Tns1*; Z. Zhu *et al*, 2020). The synergistic action of reduced miRNAs and increase in their target mRNAs, such as *Unc5c* (Latil *et al*, 2003), *Thsd4* (Wu *et al*, 2020), *Sparc* (de Oliveira‐Barros *et al*, 2021), *Runx1t1* (Kan *et al*, 2010) and *Pcdh17* (Costa *et al*, 2011), known to promote prostate cancer, may contribute further to the prostate lesions and tumours in the A537T‐TRAMP mice (Fig 6E). In the KO‐TRAMP mice, *Vcan* (with a role in cell adhesion and tumour progression; Ricciardelli *et al*, 1998) and *Myb* proto‐oncogene (Li *et al*, 2014) levels, that are markers of prostate metastasis (Edwards, 2012), were increased, which correlated with reduced miR‐669c‐5p, and reduction in miR‐676‐5p correlated with increased expression of insulin‐like growth factor‐1 (*Igf1*) that promotes cell growth and malignancy (Perry *et al*, 2017; Fig 6F). A notable difference between the two mutant TRAMP lines compared with the WT‐TRAMP mice was an increase in several different miRNAs that target semaphorin mRNAs (*Sema3a*, *c*, *5a* and *6a*) and *Nox4* and the reduced levels of their targets in the KO‐TRAMP mice (Fig 6F), which contrasts with reduced miRNAs that showed an increase in semaphorins and *Nox4* mRNA levels in the A537T‐TRAMP mice. This difference may account for the increased tumour formation in the prostates of the A537T‐TRAMP, where semaphorins and *Nox4* were increased, compared with the KO‐TRAMP mice, as these proteins can contribute to more invasive prostate cancer (Blanc *et al*, 2011; Roy *et al*, 2015; L*iu et al*, 2015a; Williamson *et al*, 2016; Peacock *et al*, 2018; Yin *et al*, 2021).

To identify the common mechanisms that drive dysplasia and tumorigenesis in the prostates of the mutant mice, we identified common miRNAs that were changed in both the KO and A537T or KO‐TRAMP and A537T‐TRAMP mice, and their mRNA targets. GO analyses identified the processes most affected by the miRNAs whose abundance was altered in common between the loss and mutation of ELAC2 (Appendix Fig S11). In the KO and A537T mice compared with controls, impaired mitochondrial function and translation led to prostate morphogenesis, development and inflammation (Appendix Fig S11A), consistent with the morphological and molecular defects identified in these mice. In the KO‐TRAMP and A537T‐TRAMP mice, the miRNA imbalance may lead to prostate cancer by upregulation of cell growth and immunometabolic pathways that promote tumour formation (Appendix Fig S11B). Together these findings indicate that ELAC2 can act as a rheostat maintaining a balance in pro‐ and anti‐tumorigenic miRNAs and when that balance is shifted by reduction or loss of ELAC2, the change in miRNA levels and consequent effects on their mRNA targets can drive tumorigenesis in the prostate.

### The *Elac2* variant contributes to prostate tumorigenesis by reducing the interaction of SFPQ and NONO with ELAC2

**Figure 7:**
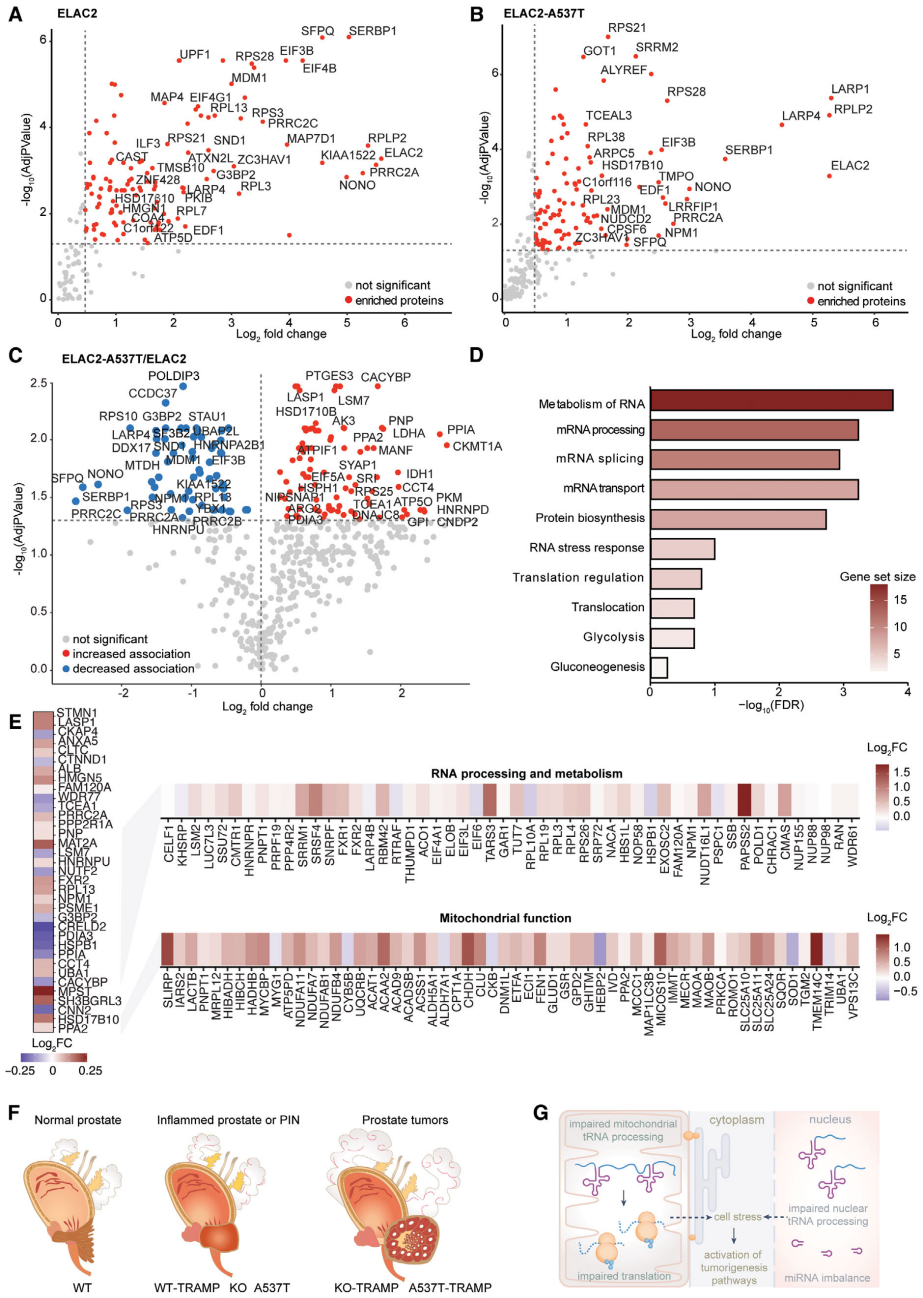
ELAC2 associates with NONO and SFPQ, and the A537T variant reduces their interaction A–CWT ELAC2‐EGFP or A537T ELAC2‐EGFP were expressed in LNCaP cells, and the associated proteins that immunoprecipitated with the EGFP antibody were detected by mass spectrometry. EGFP‐only protein was used as a control. Volcano plots are showing the enriched proteins with WT ELAC2 (A) and A537T ELAC2 (B) proteins and (C) shows the proteins that have reduced or increased association with A537T ELAC2. Volcano plots comparing Log_2_ fold change against the −log^10^ *P*‐value and significant (*P* < 0.05) enriched proteins are shown in red, and proteins with reduced association are shown in blue for three biologically independent experiments.DGene ontology processes associated with the proteins identified in (C). Bar graphs show log10 (FDR), and colour scale represents fold change for each process.ELevels of the proteins identified by immunoprecipitation detected by mass spectrometry in the prostates from A537T mice and proteins associated with MAPK signalling, RNA metabolism and mitochondrial function.FThe KO and A537T mice are physiological models of prostate inflammation and PIN formation, whereas the KO‐TRAMP and A537T‐TRAMP mice model the onset and progression of prostate cancer.GELAC2 regulates the levels of tRNAs and ncRNAs to maintain prostate health, reduction in ELAC2 impairs nuclear and mitochondrial tRNA processing and dysregulates ncRNAs levels activating their mRNA targets that potentiate inflammation, cell growth and aberrant morphogenesis.

To understand whether changes in ELAC2 protein interactions can contribute to prostate tumorigenesis, we investigated the effects of the A537T mutation on the association of ELAC2 with other proteins compared with the wild‐type ELAC2 protein. Wild‐type ELAC2 or mutant A537T ELAC2 proteins were expressed with EGFP at their C‐termini, immunoprecipitated from prostate cancer LNCap cells and proteomic analyses were carried out to identify changes in associated proteins (Fig 7A and B, and Dataset EV6). The wild‐type or A537T mutant ELAC2 protein was most enriched, validating that they were effectively immunoprecipitated, along with nuclear and mitochondrial proteins involved in gene expression and metabolism, translation and cell organisation, including HSD17β10, a constituent of the 5′ tRNA processing machinery in mitochondria (Holzmann *et al*, 2008; Rackham *et al*, 2016; Fig 7A–C). Unexpectedly, we identified that ELAC2 associates with the splicing factor proline‐ and glutamine‐rich (SFPQ) and non‐POU domain containing octamer binding (NONO) proteins (Fig 7A) that can sequester mRNAs encoding mitochondrial proteins within the nuclear paraspeckles they form (Wang *et al*, 2018). The association of the mutant A537T ELAC2 with SFPQ and NONO was lowered by 2.3 and 2.5‐fold, respectively, compared with the wild‐type ELAC2 (Fig 7C), indicating that the mutation reduces the stability of their interaction. In addition, the A537T mutation altered the association of ELAC2 with the mitochondrial HSD17β10 enzyme. We identified the functional roles of proteins whose association was altered with the mutant ELAC2 protein (Fig 7C) and found that they regulate RNA and energy metabolism (Fig 7D), consistent with the processes that were most impaired in the mutant mice compared with controls. These findings suggest that reduced association of ELAC2 with nuclear coregulators, such as NONO and SFPQ, may induce transcriptional activation of tumorigenic pathways and predispose to prostate cancer. This is corroborated in the prostates of A537T mice, where proteins that were found to associate with the mutant ELAC2 protein (Fig 7A–C), had significantly altered levels, as did a range of proteins also involved in mitochondrial and RNA metabolism (Fig 7E). We conclude that defects in mitochondrial and nuclear tRNA processing can lead to inflammation in the prostate and with a second insult can predispose to prostate cancer (Fig 7F).

## Discussion

The *ELAC2* gene was identified as a prostate cancer susceptibility gene more than two decades ago, but its mechanistic and physiological roles in prostate cancer development had not been explored until this work. Interestingly, different GWAS have reported contrasting findings about the predisposing impact of the *ELAC2* A541T variant to prostate cancer (Rebbeck *et al*, 2000; Suarez *et al*, 2001; Tavtigian *et al*, 2001; Vesprini *et al*, 2001; Wang *et al*, 2001; Xu *et al*, 2001), although none of the studies considered the presence of secondary somatic mutations that may be necessary for prostate tumorigenesis (Knudson, 1996). Therefore, we investigated the effects of the A537T variant *in vivo* and show that it can promote hyperplasia and inflammation of the prostate with age; however, it does not lead to prostate cancer in mice on its own (Fig 7F). Prostate cancer progression and metastasis in TRAMP mice resembles human prostate cancer progression and have been widely used to study prevention, treatment and metastasis (Jeet *et al*, 2010). The TRAMP oncogenic effects were greatly exacerbated by the A537T variant (Fig 7F), indicating that it increases the risk of prostate cancer and promotes malignancy in the prostate. It would be of significant interest to revisit GWAS cohorts to identify secondary oncogenic mutations in patients with *ELAC2* variants to identify potential epistatic regulators of prostate cancer.

Mutations in *ELAC2* or deletion of the mouse *Elac2* in the heart lead to cardiomyopathies due impaired 3′ tRNA processing that causes loss of mature tRNAs required for translation, consequently resulting in protein synthesis defects and energy dysfunction (Haack *et al*, 2013; Siira *et al*, 2018). Our transcriptomic and proteomic analyses revealed that prostate‐specific deletion or mutation of *Elac2* also impaired nuclear and mitochondrial tRNA processing in the prostate. Impaired RNA metabolism resulted in potentiation of tumorigenic cell growth and differentiation via mitochondrial dysfunction and activation of immunometabolic pathways in the prostates with reduced ELAC2 activity. In addition to mitochondrial RNA processing, *ELAC2* has been shown to be essential for the processing of nuclear pre‐tRNAs and also the balanced production of miRNAs, which are important for the regulation of gene expression (Siira *et al*, 2018). miRNAs have a range of mRNAs targets (Iorio & Croce, 2017), and consequently, specific miRNAs can predispose to or protect against cancer. In the A537T mice, we identified specific sets of miRNAs that target mRNAs which in turn drive immune and innate stress responses stimulating cell growth with tumorigenic potential, and when coupled with the oncogenic driver on the TRAMP background contribute to prostate cancer development (Fig 7G). Although we identified similar gene expression networks in the KO mice, we also identified unique miRNAs that were altered in the absence of ELAC2 and targeted mRNAs that countered the tumorigenicity, possibly explaining the less invasive tumour formation in the KO‐TRAMP mice compared with the A537T‐TRAMP mice.

We identified that the ELAC2 protein associates with SFPQ and NONO and that the A537T variant reduces the stability of this interaction. NONO and SFPQ are RNA‐binding proteins that have been shown to play a role in telomere stability (Petti *et al*, 2019), intron splicing (Stagsted *et al*, 2021) and as coregulators of androgen receptor‐mediated transcription (Kuwahara *et al*, 2006). Reduction of NONO and SFPQ has been shown to cause telomere instability and increased replication defects (Petti *et al*, 2019), suggesting that the reduced levels of these proteins in the mutant TRAMP mice may be one of the consequences of impaired nuclear RNA processing that can potentiate or contribute to tumorigenesis in the prostate. It is also possible that reduction in NONO and SFPQ can lead to intron retention, which has been implicated in aggressive prostate cancer (Stagsted *et al*, 2021). The fact that NONO/SFPQ, which can regulate both nuclear and, indirectly, mitochondrial gene expression, are co‐regulated via a physical association with ELAC2, that directly controls gene expression in both compartments, is intriguing and highlights an emerging network that exists to balance the functions of these two organelles.

We have established new and physiologically relevant models of prostate tumorigenesis and cancer that can be used for drug screens and therapeutic development in future or combined with other models to investigate modulators of prostate cancer. In addition, we mechanistically validate the predisposing risk of the A541T variant in *ELAC2* for prostate cancer, opening the way to screen for this variant in the human population along with other oncogenic mutations that can cause or predispose to prostate cancer.

## Materials and Methods

### Animals and housing

*ELAC2 Pb*‐Cre (KO) and *ELAC2* Ala537Thr (A537T) mice on a C56NL/6N background were generated by the Monash Genome Modification Platform (MGMP, part of the Australian Phenomics Network (APN), Monash University, Melbourne, Australia) and breed with homozygous TRAMP mice to generate homozygous KO/heterozygous TRAMP and homozygous A537T/heterozygous TRAMP mice. Male age‐ and littermate‐matched (10‐ and 30‐week‐old) WT, WT‐TRAMP, knockout (KO/KO‐TRAMP) and point mutation (A537T/A537T‐TRAMP) mice were housed in standard cages (45 × 29 × 12 cm) under a 12‐h light/dark schedule (lights on 7 a.m. to 7 p.m.) in controlled environmental conditions of 22 ± 2°C and 50 ± 10% relative humidity. Mice were fed a normal chow diet (Rat and Mouse Chow, Specialty Feeds, Perth, Australia). Food and water were available ad libitum. This study was approved by the Animal Ethics Committee of the UWA and performed in accordance with Principles of Laboratory Care from the NHMRC *Australian code for the care and use of animals for scientific purposes*, 8^th^ Edition 2013 (RA/3/300/129).

### Prostate dissection

Individual prostate lobes were dissected as described previously by Zingiryan *et al* (2017). Briefly, an OPMI 6‐SFC Universal Microscope (Zeiss) was used to remove each prostate lobe to isolate high quality and pure tissue. Upon removal of the urogenital system from the sacrificed animal, all tissue was completely submerged in PBS. The seminal vesicles, bladder, urethra and surrounding connective or adipose tissue were removed, and then, the individual ventral, lateral, anterior and dorsal lobes were dissected and frozen in liquid nitrogen.

### Mitochondrial isolation

Mitochondria were isolated from homogenised whole prostates (seminal vesicles, bladder and urethra removed) and purified by differential centrifugation as described previously (Perks *et al*, 2017) with some modifications. Prostate homogenates were centrifuged at 1,000 *g* for 5 min at 4°C, the supernatant was retained, and pellet was resuspended and centrifuged at 1,000 *g* for 5 min at 4°C before both supernatants were pooled and centrifuged at 10,000 *g* for 10 min at 4°C. The mitochondrial pellet was washed twice in 250 mM sucrose, 5 mM Tris, 1 mM EGTA, pH 7.4, and mitochondrial protein concentration was quantified using the bicinchoninic acid (BCA) assay.

### Immunoblotting

Specific proteins were detected using rabbit monoclonal antibodies against ELAC2 (10071‐AP and PA5‐96587) and HSP60 (ab137706). All primary antibodies were diluted 1:1,000 using the Odyssey blocking buffer (LI‐COR). IR Dye 800CW Goat Anti‐Rabbit IgG or IR Dye 680LT Goat Anti‐Mouse IgG (LI‐COR) secondary antibodies (diluted 1:10,000) were used, and the immunoblots were visualised and quantified using the Odyssey Infrared Imaging System (LI‐COR).

### Reverse‐phase protein arrays

Prostate homogenates were prepared from the anterior and dorsal lobes of 10‐ and 30‐week‐old WT, WT‐TRAMP, KO, KO‐TRAMP, A537T and A537T‐TRAMP mice using a bead beater in 150 μl of CLB1 lysis buffer (7 M urea, 2 M thiourea, 4% (w/v) CHAPS, 1% (w/v) dithiothreitol, 4 mM spermidine, 2% (w/v) pharmalyte, pH 3–10) containing EDTA‐free Complete protease inhibitor and PhosSTOP (Roche). The tissue homogenate protein concentration was quantified using the Bradford assay, and 2 mg/ml of sample was sent to the Victorian Centre for Functional Genomics (VCFG (Melbourne, Australia)) for reverse‐phase protein array (RPPA) analysis as described by Dawson & Carragher, 2014 and Yamada, 2014.

### RNA sequencing and analyses

RNA was isolated from the ventral and lateral prostate lobes as recommended by Zingiryan *et al* (2017), using the miRNeasy Mini kit (Qiagen) incorporating an on‐column RNase‐free DNase digestion to remove all DNA. RNA sequencing was performed on total prostate RNA from three of each control (WT or TRAMP) and the four mutant mouse lines by the Australian Genomic Research Facility (AGRF, Melbourne, Australia). Sequencing was performed using the Illumina NovaSeq platform, according to the Illumina TruSeq RNA protocol and as we have done previously (Rudler *et al*, 2019). Sequenced reads were trimmed with Trim Galore (Krueger Felix, 2012) (0.4.1) using cutadapt (Martin, 2011) (1.18) (with parameters: ‐‐paired ‐‐fastqc). Trimmed reads were aligned to the mouse genome downloaded from GENCODE (GRCm38.p6, primary assembly) masked for nuclear mitochondrial sequences with the vM24 GENCODE mouse gene annotation using STAR (Dobin *et al*, 2013) (v2.7.3a) with custom mitochondria annotations. The transcriptome alignments produced by STAR were quantified with Salmon (Patro *et al*, 2017) (‐l ISR ‐‐seqBias ‐‐gcBias) against a transcriptome FASTA file produced from the GENCODE gene annotation and genome sequence with gffread (Pertea & Pertea, 2020). The output files were imported into R (3.6.3) (https://www.R‐project.org) with tximport (1.14; Soneson *et al*, 2015) and were analysed for differential expression against wild types with DESeq2 (1.26) (Love *et al*, 2014) using apeglm (1.8.0) (A. Zhu *et al*, 2019) for effect size shrinkage estimation.

### miRNA sequencing and analyses

Sequencing was performed using the Illumina NovaSeq platform, using the Illumina miRNA‐Seq RNA‐Seq protocol according to the manufacturer's instructions. Paired‐end sequenced reads were merged, removing adapters, with BBMerge (Bushnell *et al*, 2017; adapter = default mininsert = 18 mininsert0 = 18 k = 31 ecct). Merged reads were mapped (‐e ‐h ‐I ‐j ‐m ‐q) and quantified (‐W) with mirDeep2 v0.1.3 (Friedländer *et al*, 2012) against the GRCm38 mouse genome and mirBase (Kozomara *et al*, 2019) mouse release 22.1 mature and precursor miRNA sequences. Duplicate entries in the miRNA count table were summed and miRNAs with unique names but identical sequences were also summed under a merged miRNA name, to ensure that each unique miRNA sequence was represented only once in the count table. Differential expression changes were tested with DESeq2 (Love *et al*, 2014), using the apeglm (Zhu *et al*, 2019) shrinkage estimator.

### miRNA‐mRNA correlation

A complete list of all known *Mus musculus* mRNA transcripts was obtained from Ensembl database GRCm39 (accession date: 19 April 2021) and matched to their corresponding targeting miRNAs as obtained from miRDB 6.0 (Chen & Wang, 2020) using MirTarget (Liu & Wang, 2019) to predict mRNA targets (accession date: 19 April 2021) retrieving miRNA names linked to Genbank accession numbers. Each Genbank accession number was then related to an Ensembl ID corresponding to an mRNA target via its mouse genome identifiers (MGI) obtained from the Mouse Genome Database (Bult *et al*, 2019). The resultant matches were recorded such that each mRNA transcript was associated with *n* miRNA transcripts that are known to target that mRNA transcript according to the source data. Each mRNA/miRNA pair was then searched for in the experimental datasets using Python 3.8 scripts with corresponding log2 fold change and abundance values being retrieved for analysis and investigation.

### Small RNA sequencing

Sequencing was performed on the Illumina NovaSeq platform, using the Perkin Elmer small RNA‐Seq protocol according to the manufacturer's instructions. Sequenced reads were trimmed with Trim Galore (v0.6.7; Krueger Felix, 2012) using cutadapt (v4.1; Martin, 2011; with parameters: ‐‐paired ‐‐fastqc). Mapping of tRNA reads was performed following Hoffmann *et al*, 2018. Briefly, an artificial mouse genome was generated from the mouse genome downloaded from GENCODE (ftp://ftp.ebi.ac.uk/pub/databases/gencode/Gencode_mouse/release_M31/gencode.vM31.primary_assembly.annotation.gtf.gz on 16 December 2022). The 3′ and 5′ genomic flanking regions of 50 nt in length for each tRNA gene were extracted and added to the mature tRNA sequences (GRCm39, downloaded from gtRNAdb on 16 December 2022) to simulate premature tRNA. These sequences were added as additional chromosomes to the artificial genome and were masked within the genomic regions using BEDTools (v2.30.0; Hoffmann *et al*, 2014). A mature tRNA genome was obtained from gtRNAdb and appended to contain 3′ CCA tails. Trimmed reads were aligned against the artificial mouse genome using Segemehl (v0.3.4; Hoffmann *et al*, 2014) with parameters allowing a minimal accuracy of 80% to accommodate for mismatches induced by the modification heavy tRNAs (parameters: ‐E 500 ‐D 3 ‐A 80 ‐M 1000 ‐H 1). Aligned reads were then filtered to extract reads that aligned to premature tRNA 3′ or 5′ flanking extensions with samtools (v1.16.1; Danecek *et al*, 2021). Unprocessed tRNA reads were then realigned against an unprocessed tRNA library containing tRNA genes with 50 nt extensions as individual chromosomes with Segemehl, requiring a higher mapping accuracy this round (parameters: ‐E 500 ‐D 3 ‐A 85 ‐M 1000 ‐H 1). Paired‐end read fragment coverage was calculated with deepTools (v3.5.1)^113^ and visualised in R (v4.2.1; RCoreTeam, 2016).

### Gene ontology

Differentially expressed transcripts identified from transcriptomic analyses were summarised by biological process gene ontologies using DAVID's (Huang *et al*, 2007) statistical overrepresentation test. Resulting GO lists were condensed and bubble plots generated using REVIGO (Supek *et al*, 2011) with allowed similarity set to small. Significant (FDR < 0.05) proteomics results were summarised by Reactome pathways with DAVID, and resulting pathways were considered enriched at FDR < 0.05. Gene list redundancies that occur as part of gene ontology analyses were reduced using a custom script in R (RCoreTeam, 2016), removing duplicates and keeping ontologies based on those with the highest FDR. Comparisons between Reactome pathways of KO and WT, A537T and WT, KO‐TRAMP and WT‐TRAMP and A537T‐TRAMP and WT‐TRAMP at 10 and 30 weeks were performed and sorted by FDR. From these changes, we visualised the top 20 significant pathways that were in common or different between the KO and A537T lines or KO‐TRAMP and WT‐TRAMP lines.

### Tissue homogenate preparation

Anterior and dorsal lobes of mouse prostates were incubated in 200 μl of CLB1 buffer containing EDTA‐free Complete protease inhibitor (Roche) for 30 min at room temperature. The samples were homogenised using a bead beater, and the homogenates were clarified at maximum speed in a benchtop centrifuge for 5 min at room temperature. The previous steps were repeated until a clear tissue homogenate was produced. The tissue homogenate protein concentration was quantified using the Bradford assay.

### Proteomics

Tissue homogenates were mixed 1:1 with lysis buffer (10% SDS with 5,100 mM Tris) to achieve samples containing 200 μg of protein and digested using the S‐trap micro columns as per the manufacturer's instructions with some modifications. Briefly, dithiothreitol was added to a final concentration of 20 mM and incubated at 70°C for 60 min. Proteins were alkylated by adding iodoacetamide to a final concentration of 40 mM and incubating at room temperature in the dark for 30 min. Proteins were acidified with 2.5 μl of 12% phosphoric acid and diluted with 165 μl of binding buffer (90% methanol, 100 mM final Tris). Samples were added to the S‐Trap Micro Spin columns (Protifi) by centrifugation at 4,000 *g* for 30 s and then subsequently washed three times by successively loading 150 μl of binding buffer and centrifuging at 4,000 *g* for 30 s. Digestion was achieved by adding 1 μg sequencing‐grade trypsin (Promega) and 25 μl of 50 mM ammonium bicarbonate and incubating overnight at 37°C. Peptides were eluted by successively adding 40 μl of 5% acetonitrile in 0.1% formic acid, 40 μl of 50% acetonitrile in 0.1% aqueous formic acid and 40 μl of 75% acetonitrile in 0.1% formic acid with a 30 s centrifugation step at 4,000 *g* between the addition of each elution buffer. The eluants were pooled, dried in a vacuum centrifuge and resuspended in 20 μl of buffer A (0.1% formic acid).

Samples were analysed using a Thermo Fisher Scientific Ultimate 3000 RSLC UHPLC and a Q‐Exactive HF mass spectrometer. Samples were injected on a reverse‐phase PepMap 100 C18 trap column (5 μm, 100 Å, 300 μm i.d. × 5 mm) at a flow rate of 15 μl/min. After 3.0 min, the trap column was switched in line with a Waters nanoEase M/Z Peptide CSH C18 resolving column (1.7 μm, 130 Å, 300 μm i.d. × 100 mm) and the peptides were eluted at a flow rate of 3 μl/min using buffer A (0.1% formic acid) and buffer B (80% acetonitrile in 0.1% formic acid) as the mobile phases. The gradient consisted of 8–2,410% B for 0 to 6 min, 10–24% B from 6 to 43 min, 24–40% B from 43–51 min, 40–95% B from 51–57 min, followed by a wash, a return of 8% buffer B and equilibration prior to the next injection. The mass spectra were obtained in DIA mode with an MS1 resolution of 120,000, automatic gain control target at 3 × 10^6^, maximum injection time at 200 ms and scan range from 400–1,100 m/z. DIA spectra were recorded at resolution 30,000 and an automatic gain control target of 2 × 10^5^. The 70 isolation windows were 10 m/z each from mass 405–1,095.

Data analysis was performed with Spectronaut version 15 (15.2.210819.50606) using direct DIA analysis and default settings. Briefly, spectra were searched against the *Mus musculus* proteome database from UniProt (Proteome ID UP000000589, downloaded 14/04/2020) with carbamidomethylation set as a fixed modification and methionine oxidation and N‐terminal acetylation as variable with 1% false discovery rate cut‐offs at the peptide spectral match, peptide and protein group levels. Quantitation was performed at the MS2 level with Q‐value data filtering and cross‐run normalisation with Q‐complete row selection.

### Histology

Fresh sections of the prostate tissue were frozen in optimal cutting temperature (OCT) medium or fixed in 10% neutral buffered formalin and then embedded in paraffin wax, sectioned in 5 μm to 10 μm sections and stained with haematoxylin and eosin. Images were acquired using a Nikon Ti Eclipse inverted microscope using Nikon 4×/10×/20× objectives (Richman *et al*, 2015; Perks *et al*, 2018; Rudler *et al*, 2019). Samples were scored based on the grading scheme developed by Suttie *et al* (2003) with additional categories added: tumour development, metastasis, lobe recognition, stroma changes, nuclei disarray and inflammatory infiltration, to adapt grading to the low‐grade phenotype of Elac2 KO and A537T mutation and the exacerbate phenotype of TRAMP‐KO and TRAMP‐A537T.

### Blood analysis

Differential cell counts were performed on blood from cardiac punctures using a Hemavet HV950FS blood analyser (Drew Scientific, Waterbury, CT), calibrated using the Multi‐Trol Control Mouse standard (ELITech Group).

### Immunoprecipitation

LNCap cells (ATCC, CRL‐1740) were cultured at 37°C with 5% CO_2_ air in Roswell Park Memorial Institute (RPMI) 1640 medium supplemented with 10% fetal bovine serum (FBS). Authenticity was confirmed by STR profiling. Cells were tested and confirmed negative for mycoplasma contamination. Cells were transfected with *ELAC2* wild‐type or *ELAC2* A541T with EGFP fused to the C‐termini using FuGene (Promega) and incubated for 72 h prior to lysis in 260 mM sucrose, 100 mM KCl, 20 mM MgCl_2_, 10 mM Tris–HCl (pH 7.5), 1% digitonin and complete EDTA‐free protease inhibitor cocktail for 30 min at 4°C. Lysates were incubated with GFP‐Trap Dynabeads (Chromotek) for 2 h at 4°C. Beads were washed in digitonin‐wash buffer (same components as lysis buffer except with 0.1% digitonin), washed again in wash buffer without digitonin and then partially digested in 50 mM Tris–HCl pH 7.5, 1 mM TCEP and 5 mM CAA supplemented with 75 ng Sequencing Grade Modified Trypsin (Promega) for 30 min at room temperature. The beads were removed using DynaMag‐2 magnet (Invitrogen), and eluates were incubated at 37°C overnight for complete digestion followed by preparation for label‐free mass spectrometry.

### Statistical analyses

Values are means ± SD of biological replicates. Two‐way Student's *t*‐test was used for most analyses assuming normal distribution unless otherwise stated, there was no blinding of data, biological replicates were used for all experiments, and the sample size is included in the Figure legends. Randomisation was not carried out, and no animals were excluded from the analyses.

## Author contributions

**Maike Stentenbach:** Data curation; formal analysis; validation; investigation; writing – original draft; writing – review and editing. **Judith A Ermer:** Formal analysis; validation; investigation; writing – review and editing. **Danielle L Rudler:** Data curation; formal analysis; validation; investigation; visualization; writing – review and editing. **Kara L Perks:** Formal analysis; validation; visualization. **Samuel A Raven:** Data curation; formal analysis; validation; visualization; methodology; writing – review and editing. **Richard G Lee:** Data curation; validation; visualization; writing – review and editing. **Tim McCubbin:** Data curation; formal analysis; validation; investigation; methodology; writing – review and editing. **Esteban Marcellin:** Resources; supervision; writing – review and editing. **Stefan J Siira:** Data curation; formal analysis; validation; investigation; visualization; methodology; writing – review and editing. **Oliver Rackham:** Conceptualization; resources; formal analysis; supervision; funding acquisition; validation; investigation; visualization; methodology; writing – review and editing. **Aleksandra Filipovska:** Conceptualization; resources; data curation; formal analysis; supervision; funding acquisition; validation; investigation; visualization; methodology; writing – original draft; writing – review and editing.

## Disclosure and competing interests statement

The authors declare that they have no conflict of interest.

## Supporting information



Appendix

Dataset EV1

Dataset EV2

Dataset EV3

Dataset EV4

Dataset EV5

Dataset EV6

Source Data for Figure 1

Source Data for Figure 2

Source Data for Figure 3

## Data Availability

Genomic data are deposited to the Gene Expression Omnibus (GEO) as GSE201184 are publicly available and the mass spectrometry proteomics data have been deposited to the ProteomeXchange (http://proteomecentral.proteomexchange.org) PXD033891 via the PRIDE partner repository.
