# Supplementary material for: Multi‐omic profiling reveals an RNA processing rheostat that predisposes to prostate cancer
Source: EMBO Mol Med. 2023 Apr 24;15(6):e17463. doi: 10.15252/emmm.202317463 (PMC10245041; doi:10.15252/emmm.202317463)
Supplement: Supplementary file 1 — Appendix [file EMMM-15-e17463-s010.pdf]

Appendix

Multi-omic profiling reveals an RNA processing rheostat that predisposes to prostate cancer

Maike Stentenbach[1,2,3], Judith A. Ermer[1,2,3], Danielle L. Rudler[1,2,3], Kara L. Perks[1,2,3], Sam Raven[1,2,3], Richard G. Lee[1,2,3], Timothy McCubbin[4], Esteban Marcellin[4], Stefan J. Siira[1,2,3], Oliver Rackham[1,2,5,6,7] and Aleksandra Filipovska[1,2,3,7†]

[1]Harry Perkins Institute of Medical Research and [2]ARC Centre of Excellence in Synthetic Biology, QEII Medical Centre, Nedlands, Western Australia 6009, Australia
[3]Centre for Medical Research, The University of Western Australia, QEII Medical Centre, Nedlands, Western Australia 6009, Australia
[4]Australian Institute for Bioengineering and Nanotechnology, The University of Queensland, 4072 Queensland, Australia
[5]School of Pharmacy and Biomedical Sciences, Curtin University, Bentley, Western Australia 6102, Australia
[6]Curtin Health Innovation Research Institute, Curtin University, Bentley, Western Australia 6102, Australia
[7]Telethon Kids Institute, Northern Entrance, Perth Children's Hospital, 15 Hospital Avenue, Nedlands, Western Australia, Australia
[*]Contact for correspondence: aleksandra.filipovska@uwa.edu.au

TABLE OF CONTENTS

Pages 3,4. Appendix Figure S1 related to Figure 1. Sequencing confirms the presence of the *ELAC2* mutations.

Pages 5,6. Appendix Figure S2 related to Figure 2. The effects of the *ELAC2* A537T variant in the prostates from 10-week-old mice.

Pages 7,8. Appendix Figure S3 related to Figure 3. The *ELAC2* A537T variant on the TRAMP background leads to increased proliferation and tumorigenesis in the prostate.

Pages 9,10. Appendix Figure S4 related to Figure 4. Loss or reduction of ELAC2 leads to nuclear precursor tRNA accumulation.

Page 10. Appendix Figure S5 related to Figure 4. Differential gene expression changes in 10-week-old mice.

Page 11. Appendix Figure S6 related to Figure 5. Proteome changes identify the same processes in the mutant lines at 10 weeks.

Pages 12,13. Appendix Figure SFigure 7 related to Figure 5. Proteome changes identify the different processes in the mutant lines.

Pages 14,15. Appendix Figure S8 related to Figure 5. Changes in the prostate mitoproteomes of 10-week-old mice.

Pages 16,17. Appendix Figure S9 related to Figure 5. Changes in the prostate mitoproteomes of 30-week-old mice.

Pages 18,19. Appendix Figure S10 related to Figure 6. MiRNA changes in the mutant relative to control mice.

Pages 19,20. Appendix Figure S11 related to Figure 6. GO processes of the dysregulated miRNA targets.

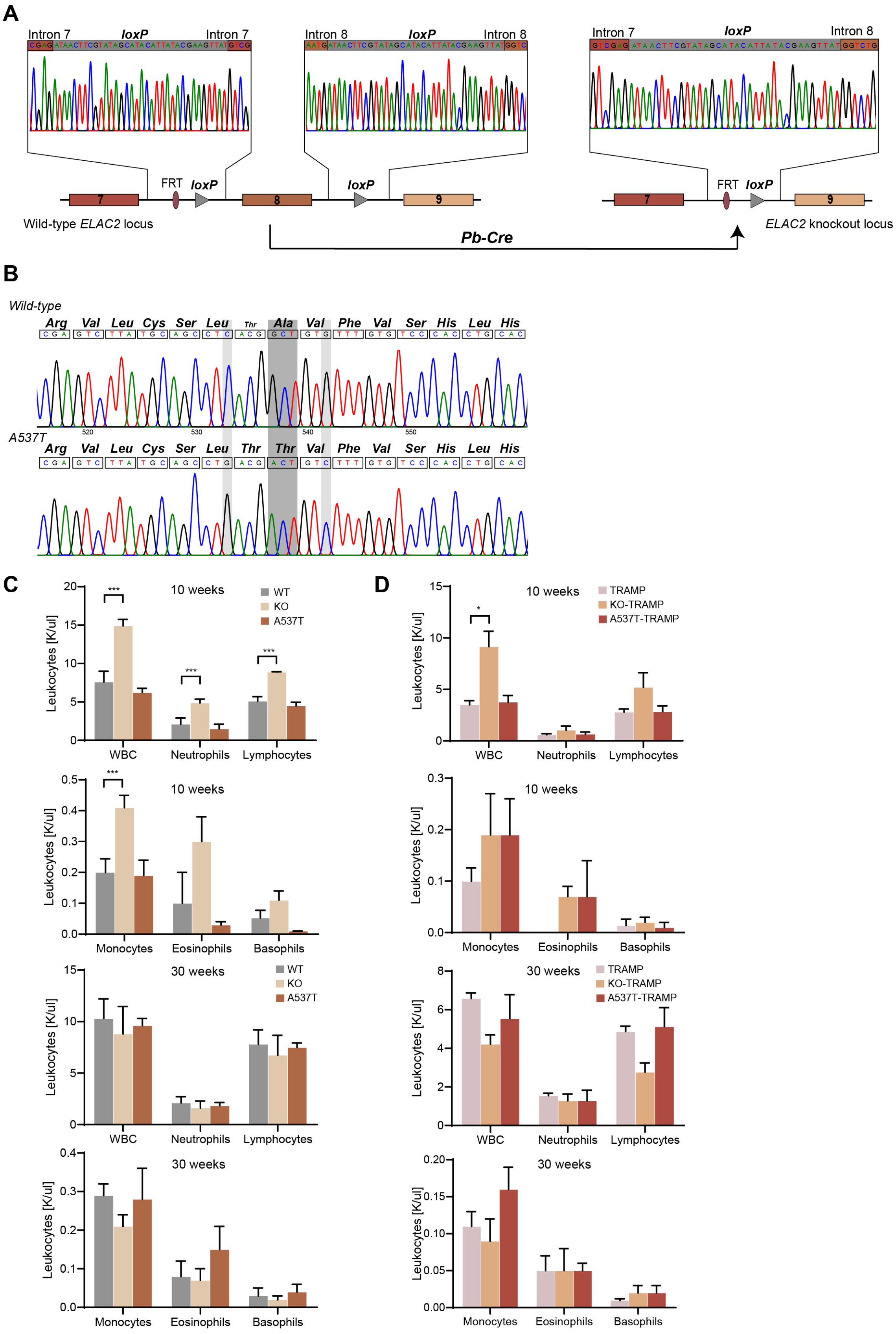

Appendix Figure S1. Sequencing confirms the presence of the *ELAC2* mutations.

(**A**) Sanger sequencing of genomic DNA from a homozygous *ELAC2* A537T confirmed the mutation in the prostate.

(**B**) Sanger sequencing of genomic DNA confirmed the homozygous *ELAC2* deletion in the prostate.

(**C**) Hemavet results for 10- and 30-week-old WT, KO, A537T and TRAMP, TRAMP-KO and TRAMP-A537T mice (n=5). WBC *** $p = 0.0007$, neutrophils *** $p = 0.001$, lymphocytes *** $p = 0.006$, monocytes *** $p = 0.003$, compared with WT for KO mice and WBC * $p = 0.03$ by two-tailed Student's *t*-test.

(**D**) Hemavet results for 10- and 30-week-old WT-TRAMP, KO-TRAMP and A537T-TRAMP mice (n=5 of each line and time point).

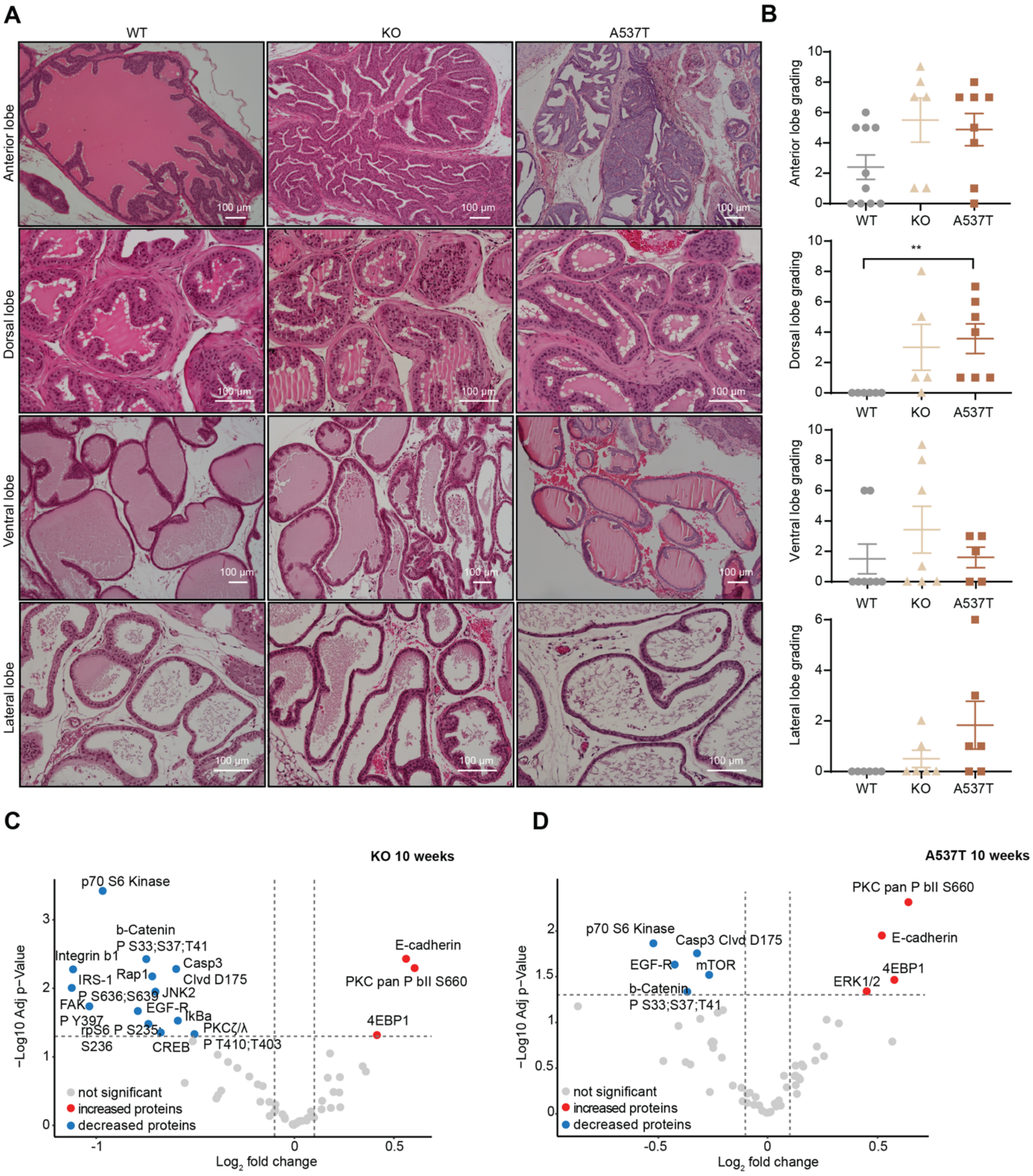

Appendix Figure S2. The effects of the *ELAC2* A537T variant in the prostates from 10-week-old mice.

(**A**) Representative images of anterior, dorsal, ventral and lateral lobe sections from 10-week-old old *ELAC2* WT (n=7), KO (n=7) and A537T (n=5) mice. 5 μm sections of each lobe were cut and stained with haematoxylin and eosin.

(**B**) Histological changes were scored according to Suttie *et al.* with additional categories added to the scoring system (described in the methods). ** $p = 0.0062$ compared with WT by two-tailed Student's t-test.

(**C**) Reverse protein phase array (RPPA) showing the protein changes in 10-week-old KO mice compared to WT mice.

(**D**) RPPA results for 10-week-old A537T mice compared with WT mice. Volcano plots in (**C**) and (**D**) comparing Log_2 fold changes against the $–log^{10}$ p-value of five mice for each genotype. Significantly ($p < 0.05$ and FC above 1) increased proteins are shown in red and significantly decreased proteins are shown in blue.

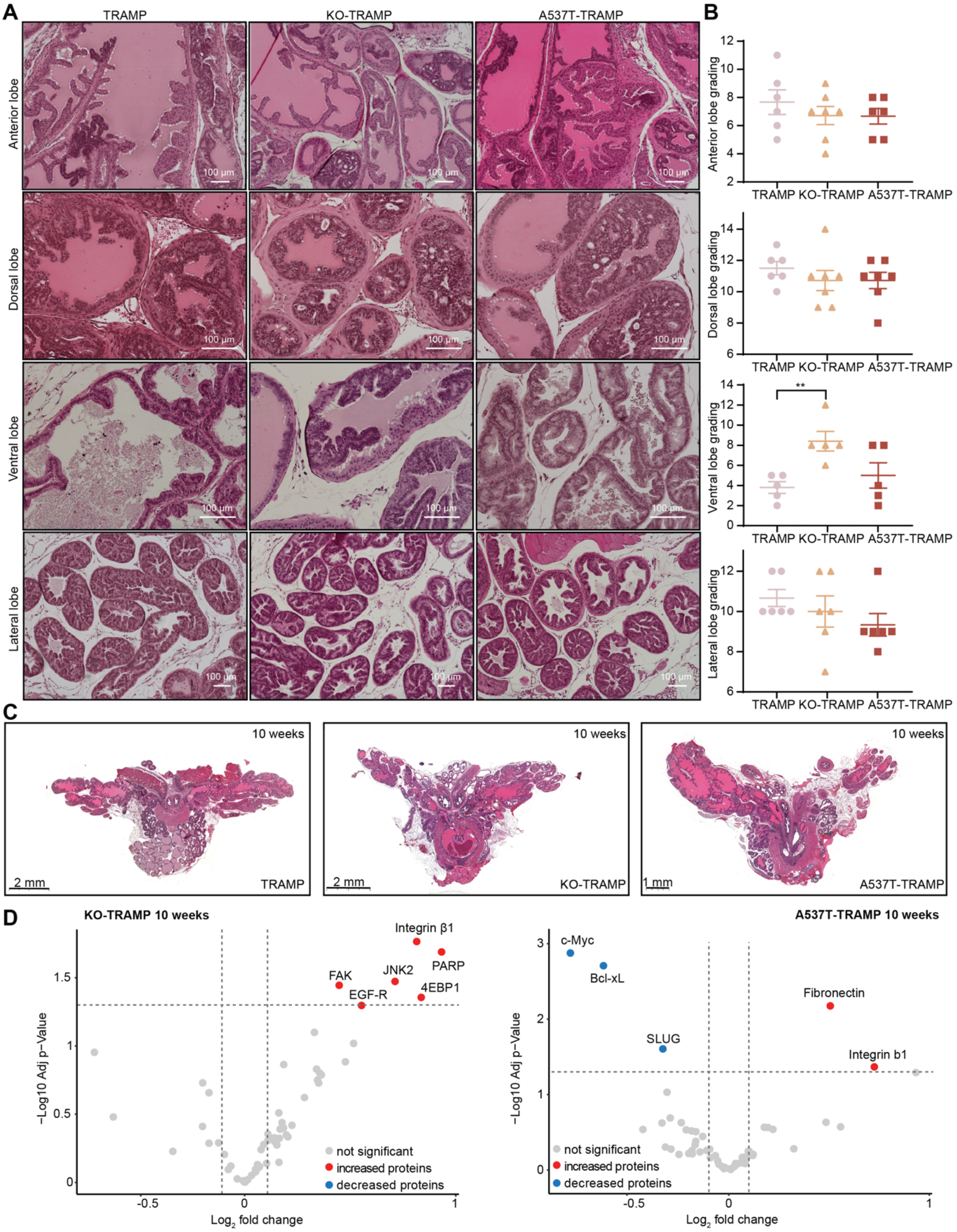

Appendix Figure S3. The *ELAC2* A537T variant on the TRAMP background leads to increased proliferation and tumorigenesis in the prostate.

(**A**) Representative images of anterior, dorsal, ventral and lateral lobe sections from 10-week-old WT-TRAMP, KO-TRAMP and A537T-TRAMP mice (n=5). 5 µm sections of each lobe were cut and stained with haematoxylin and eosin.

(**B**) Histological changes scored according to Suttie et al. with additional categories. ** p = 0.0038, compared with WT-TRAMP by two-tailed Student's t-test.

(**C**) Representative histological images of complete prostate sections from 10-week-old WT-TRAMP, KO-TRAMP and A537T-TRAMP showing the prominent tumours (black arrows) in the mutant strains compared to the WT-TRAMP mice.

(**D**) Reverse protein phase array (RPPA) results for 10-week-old KO-TRAMP mice (n=5) compared with WT-TRAMP mice (n=5).

(**E**) RPPA results for 10-week-old A537T-TRAMP mice (n=4) compared with WT-TRAMP mice (n=3). Volcano plots comparing Log_2 fold changes against the $–log^{10}$ p-value of five mice for each genotype. Significantly (p < 0.05 and FC above 1) increased proteins are shown in red and significantly decreased proteins are shown in blue.

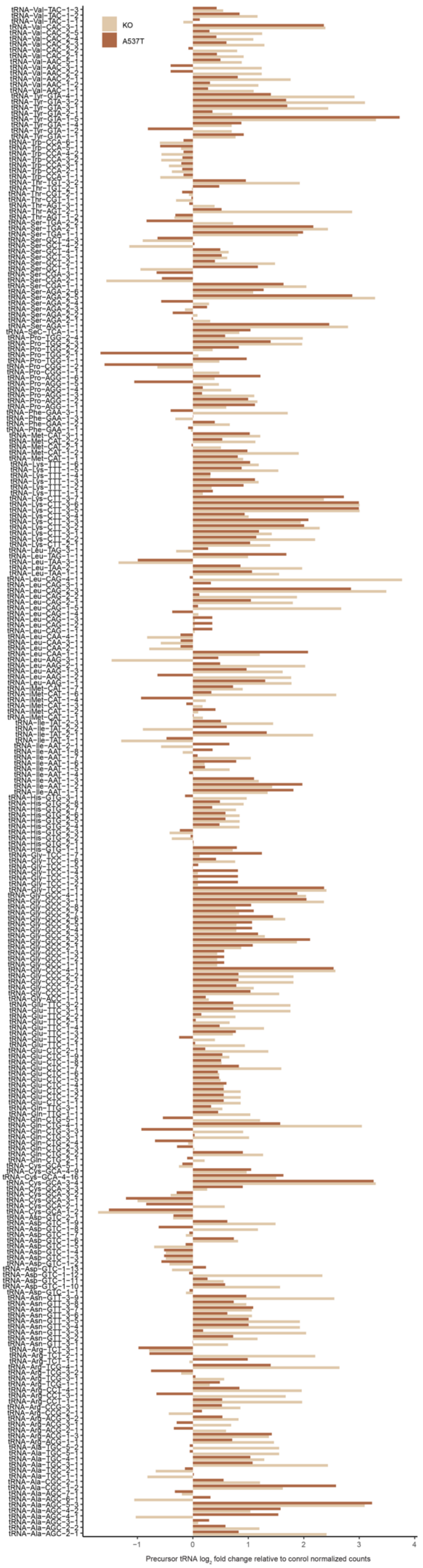
KO
A537T
Precursor tRNA log$_2$ fold change relative to conrol normalized counts

Appendix Figure S4 related to Figure 4. Loss or reduction of ELAC2 leads to nuclear precursor tRNA accumulation. The median relative abundance (normalized counts) of precursor tRNA mapping reads was calculated as $\log_2$ fold difference between control and KO or A437T mutant mice (n = 3 of each genotype).

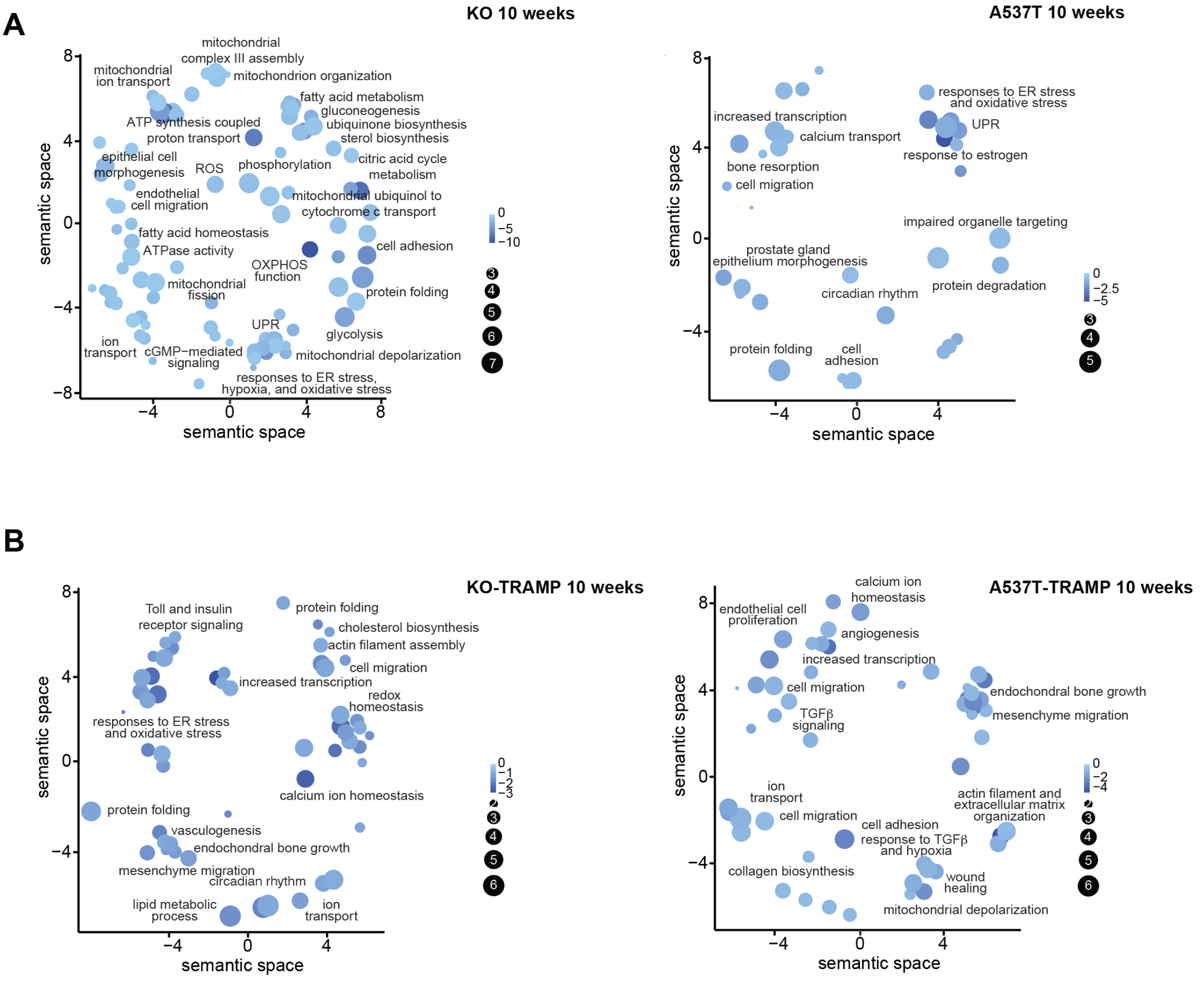

Appendix Figure S5 related to Figure 4. Differential gene expression changes in 10-week-old mice. Gene ontology changes in gene expression show the significantly changing biological processes in 10-week-old (**A**) KO and A537T or (**B**) KO-TRAMP and A537T-TRAMP mice compared to their respective controls (n=3 of each line). The colour scale represents fold change (FC) for each pathway and set size shows the number of genes within each pathway.

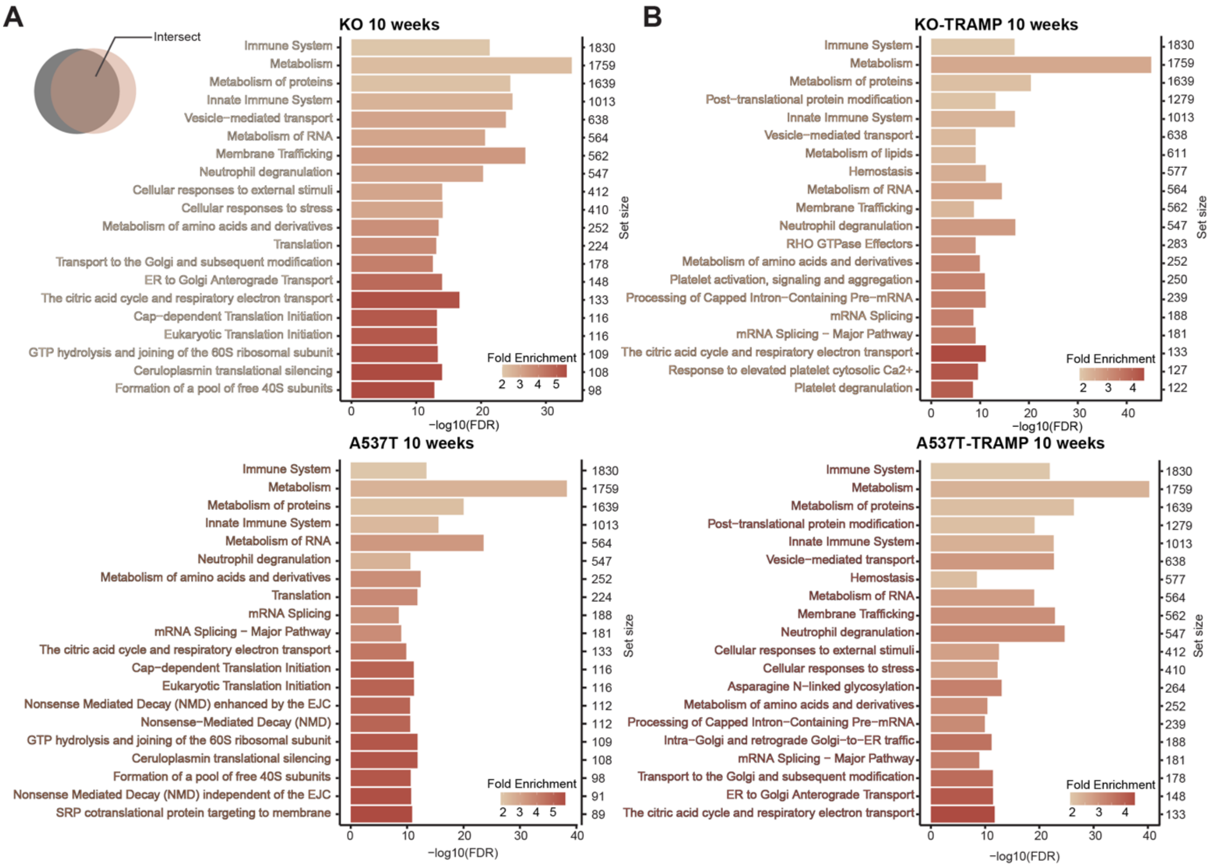

Appendix Figure S6 related to Figure 5. Proteome changes identify the same processes in the mutant lines at 10 weeks. Proteome changes in prostates isolated from the KO and A537T mice compared to WT control mice at 10 weeks (n=5) (**A**) and changes between KO-TRAMP and A537T-TRAMP mice relative to WT-TRAMP mice at 10 weeks (n=5) (**B**), based on the reactome pathway determined using PANTHER. The results show the top 20 pathways with the highest –$\log_{10}$(FDR) for each line, relative to the respective control, that are also identified as significant in the corresponding mouse line. The colour scale represents fold change (FC) for each pathway and set size is the number of genes within each pathway.

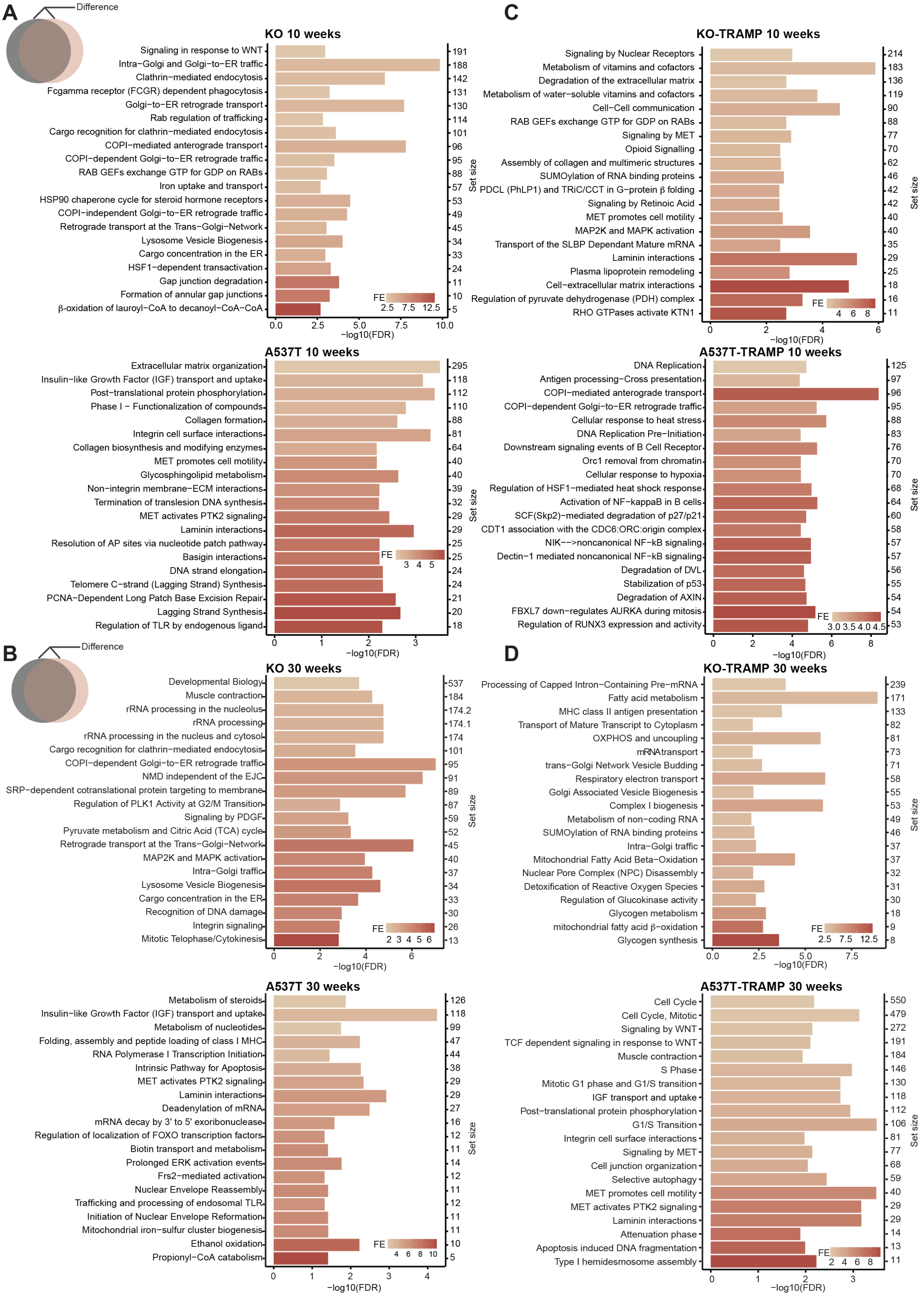

Appendix Figure S7. Proteome changes identify the different processes in the mutant lines. Proteome changes in prostates isolated from the KO and A537T mice compared to WT

control mice at 10 (**A**) and 30 weeks (**B**) and changes between KO-TRAMP and A537T-TRAMP mice relative to WT-TRAMP mice at 10 (**C**) and 30 weeks (**D**), based on the reactome pathway determined using PANTHER (n=5 mice of each line). The results show the top 20 pathways with the highest $-\log_{10}$(FDR) for each line, relative to the respective control, that are also identified as significant in the corresponding mouse line. The colour scale represents fold change (FC) for each pathway and set size is the number of genes within each pathway.

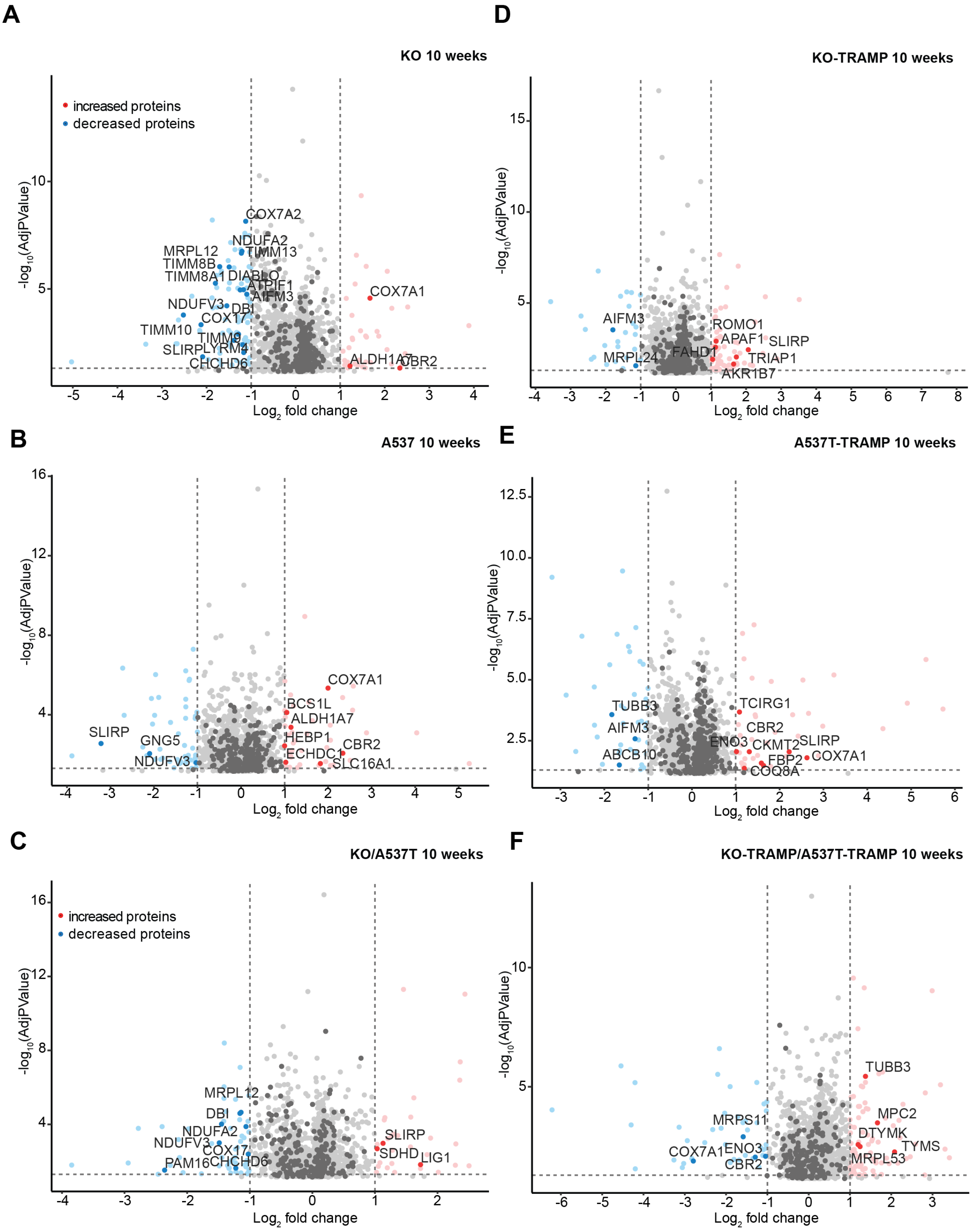

Appendix Figure S8 related to Figure 5. Changes in the prostate mitoproteomes of 10-week-old mice. Changes in mitochondrial proteins in prostates from 10-week-old (**A**) KO, and (**B**) A537T compared to WT mice, (**C**) KO mice compared to A537T mice, (**D**) KO-TRAMP and (**E**) A537T-TRAMP mice compared to TRAMP mice, and (**F**) KO-TRAMP

mice compared to A537T-TRAMP mice. Volcano plots comparing Log_2FC against the –log10 p-value of five mice for each genotype. Significantly ($p < 0.05$ and FC above 1) increased proteins are shown in red and significantly decreased proteins are shown in blue (n=5).

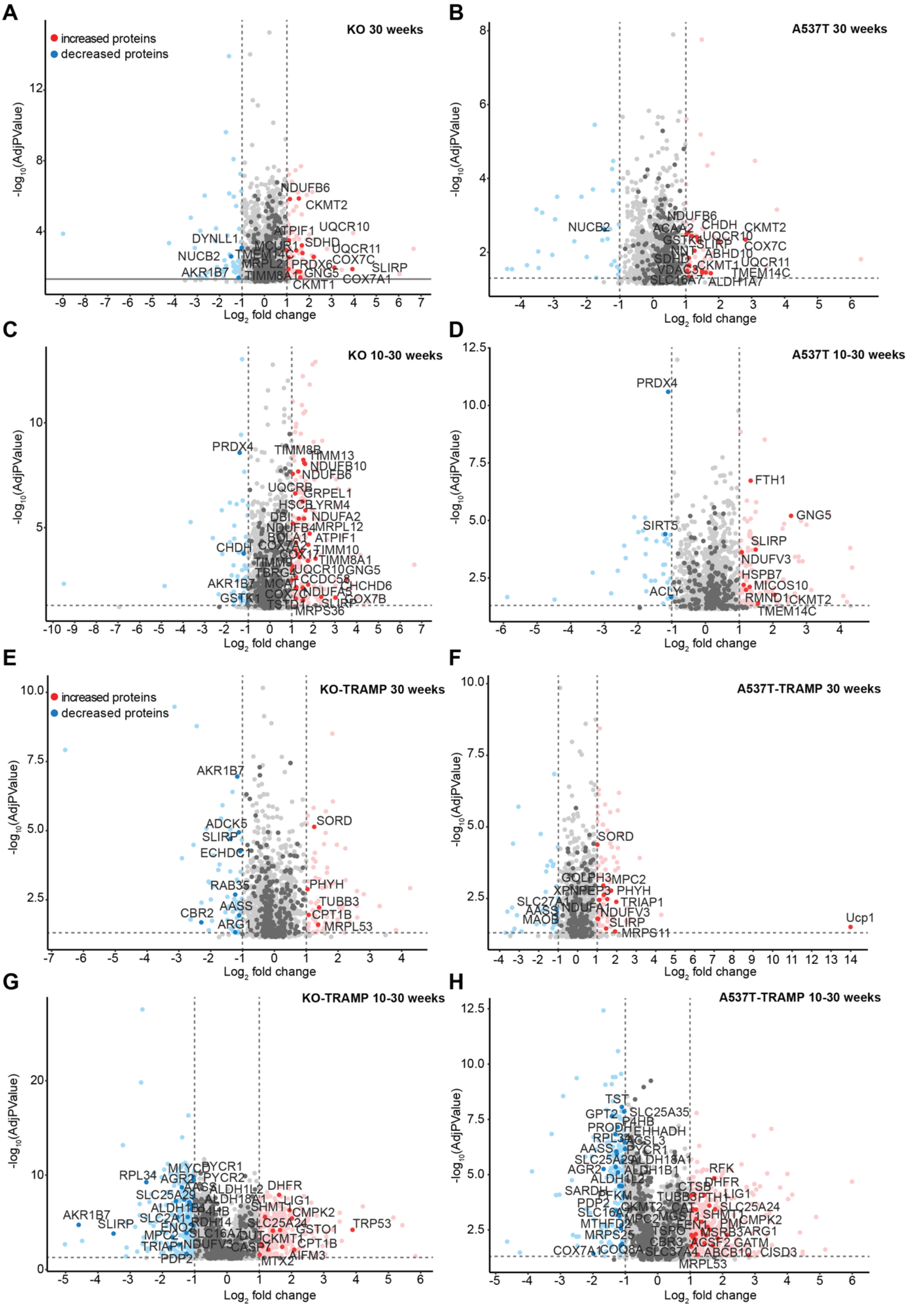
A
KO 30 weeks
increased proteins
decreased proteins
-log10(AdjPValue)
Log2 fold change
NDUFB6
CKMT2
ATPIF1
UQCR10
DYNLL1
SDHD
UQCR11
NUCB2
COX7C
PRDX6
SLIRP
AKR1B7
GNG5
COX7A1
CKMT1
B
A537T 30 weeks
NUCB2
NDUFB6
CHDH
CKMT2
UQCR10
COX7C
ABHD10
UQCR11
TMEM14C
ALDH1A7
SLC16A7
C
KO 10-30 weeks
PRDX4
TIMM8B
TIMM13
NDUFB10
NDUFB6
UQCRB
GRPEL1
LYRM4
NDUFA2
MRPL12
ATPIF1
CHDH
TIMM10
TIMM8A1
GNG5
AKR1B7
CHCHD6
GSTK1
COX7B
MRPS36
D
A537T 10-30 weeks
PRDX4
FTH1
GNG5
SIRT5
SLIRP
NDUFV3
HSPB7
MICOS10
ACLY
RMND1
CKMT2
TMEM14C
E
KO-TRAMP 30 weeks
increased proteins
decreased proteins
AKR1B7
SORD
ADCK5
SLIRP
ECHDC1
RAB35
PHYH
TUBB3
AASS
CPT1B
CBR2
ARG1
MRPL53
F
A537T-TRAMP 30 weeks
SORD
GOLPH3
MPC2
XPNPEP3
PHYH
SLC27A1
TRIAP1
AASS
NDUFA1
NDUFV3
MAOB
SLIRP
MRPS11
Ucp1
G
KO-TRAMP 10-30 weeks
AKR1B7
SLIRP
RPL34
MLYCD
PYCR1
PYCR2
DHFR
LIG1
SHMT1
CMPK2
TRP53
GSTO1
CPT1B
AIFM3
MTX2
PDP2
H
A537T-TRAMP 10-30 weeks
TST
GPT2
SLC25A35
P4HB
PRODH
EHHADH
AASS
SLC25A29
ALDH18A1
AGR2
ALDH1B1
RFK
DHFR
LIG1
SARDH
SLC25A24
CMPK2
ARG1
MTHFD2
MRPS25
COX7A1
COQ8A
ABCB10
CISD3
MRPL53

Appendix Figure S9 related to Figure 5. Changes in the prostate mitoproteomes of 30-week-old mice. Changes in mitochondrial proteins in prostates from 30-week-old (**A**) KO, and (**B**) A537T compared to WT mice, (**C**) KO mice compared to WT mice from 10-30 weeks and (**D**) A537T mice compared to WT mice from 10-30 weeks. Changes in mitochondrial proteins in prostates from 30-week-old (**E**) KO-TRAMP, and (**F**) A537T-TRAMP compared to WT-TRAMP mice, (**G**) KO-TRAMP mice compared to WT-TRAMP mice from 10-30 weeks and (**H**) A537T-TRAMP mice compared to WT-TRAMP mice from 10-30 weeks. Volcano plots comparing Log_2FC against the –log10 p-value of five mice for each genotype. Significantly ($p < 0.05$ and FC above 1) increased proteins are shown in red and significantly decreased proteins are shown in blue (n=5).

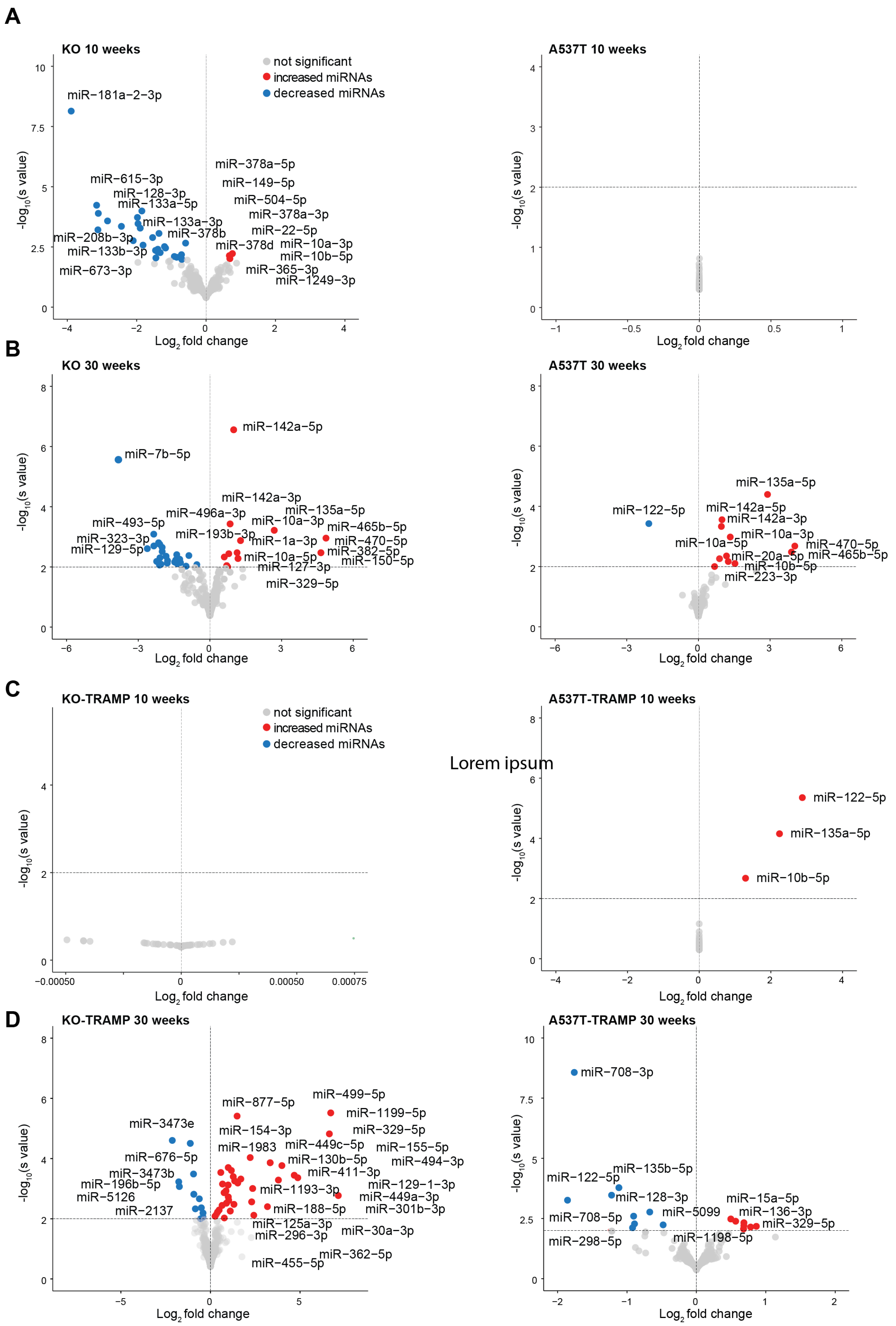

A
KO 10 weeks
not significant
increased miRNAs
decreased miRNAs
miR−181a−2−3p
miR−615−3p
miR−128−3p
miR−133a−5p
miR−133a−3p
miR−378b
miR−208b−3p
miR−133b−3p
miR−673−3p
miR−378a−5p
miR−149−5p
miR−504−5p
miR−378a−3p
miR−22−5p
miR−378d
miR−10a−3p
miR−10b−5p
miR−365−3p
miR−1249−3p
A537T 10 weeks
-log10(s value)
Log2 fold change
B
KO 30 weeks
miR−142a−5p
miR−7b−5p
miR−142a−3p
miR−496a−3p
miR−135a−5p
miR−10a−3p
miR−465b−5p
miR−493−5p
miR−193b−3p
miR−1a−3p
miR−470−5p
miR−323−3p
miR−129−5p
miR−10a−5p
miR−382−5p
miR−127−3p
miR−150−5p
miR−329−5p
A537T 30 weeks
miR−135a−5p
miR−122−5p
miR−142a−5p
miR−142a−3p
miR−10a−3p
miR−10a−5p
miR−470−5p
miR−20a−5p
miR−465b−5p
miR−10b−5p
miR−223−3p
C
KO-TRAMP 10 weeks
not significant
increased miRNAs
decreased miRNAs
A537T-TRAMP 10 weeks
Lorem ipsum
miR−122−5p
miR−135a−5p
miR−10b−5p
D
KO-TRAMP 30 weeks
miR−877−5p
miR−499−5p
miR−1199−5p
miR−3473e
miR−154−3p
miR−329−5p
miR−1983
miR−449c−5p
miR−155−5p
miR−676−5p
miR−130b−5p
miR−494−3p
miR−3473b
miR−411−3p
miR−196b−5p
miR−1193−3p
miR−129−1−3p
miR−5126
miR−449a−3p
miR−188−5p
miR−301b−3p
miR−2137
miR−125a−3p
miR−296−3p
miR−30a−3p
miR−455−5p
miR−362−5p
A537T-TRAMP 30 weeks
miR−708−3p
miR−135b−5p
miR−122−5p
miR−128−3p
miR−15a−5p
miR−708−5p
miR−5099
miR−136−3p
miR−329−5p
miR−298−5p
miR−1198−5p

Appendix Figure S10 related to Figure 6. MiRNA changes in the mutant relative to control mice. Volcano plots showing miRNA changes in three of each (**A**) 10-week old KO and A537T, (**B**) 30-week old KO and A537T mice as well as (**C**) 10-week old KO-TRAMP and A537T-TRAMP, (**D**) 30-week old KO-TRAMP and A537T-TRAMP mice relative to three respective control mice show significantly increased (red) and decreased (blue) miRNAs (n=3 of each line and time point).

A

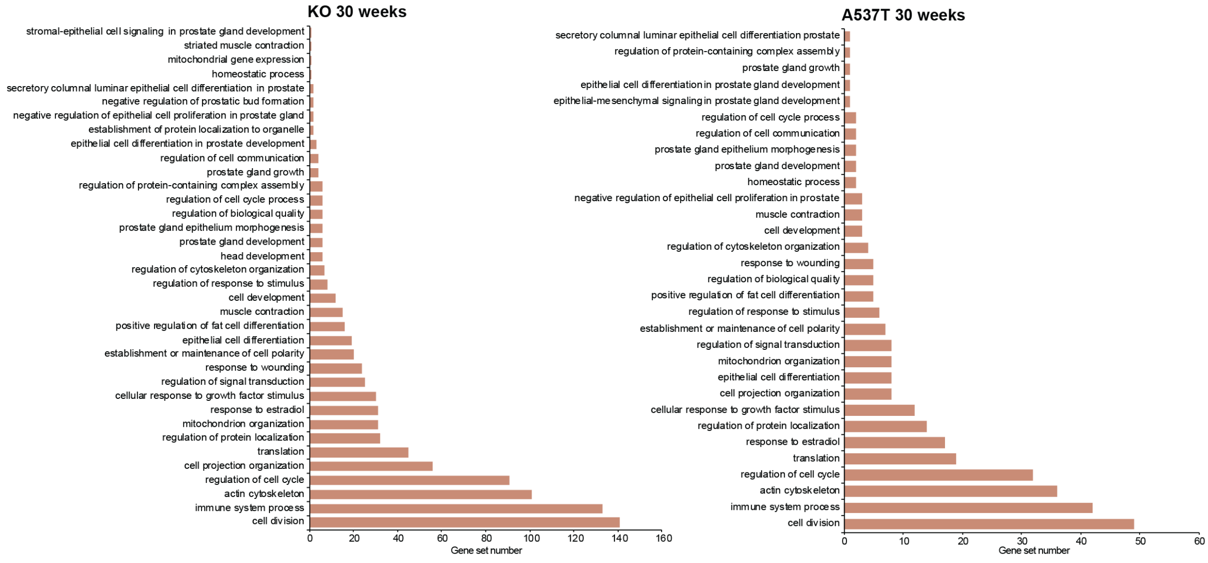

B

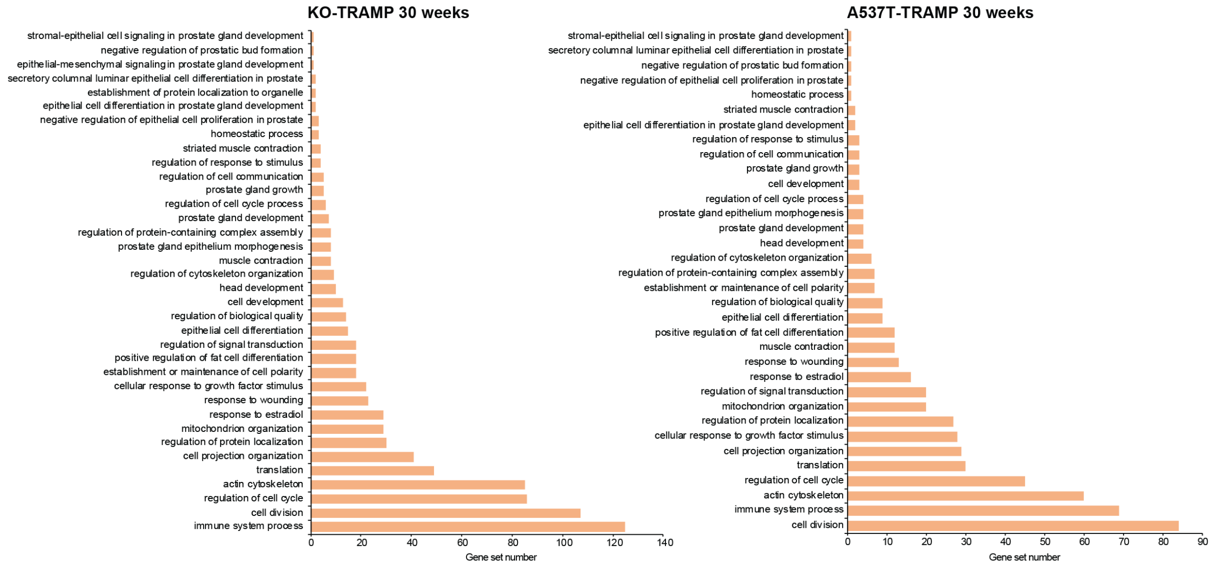

Appendix Figure S11 related to Figure 6. GO processes of the dysregulated miRNA targets. GO analyses were used to summarise the changes identified in the significantly changing miRNA targets of the 30-week-old (**A**) KO and A537T relative to WT mice or (**B**) KO-TRAMP and A537T-TRAMP mice relative to WT-TRAMP mice (n=3 of each line).
