# Supplementary figures and images for: Multi‐omic profiling reveals an RNA processing rheostat that predisposes to prostate cancer
Source: EMBO Mol Med. 2023 Apr 24;15(6):e17463. doi: 10.15252/emmm.202317463 (PMC10245041; doi:10.15252/emmm.202317463)

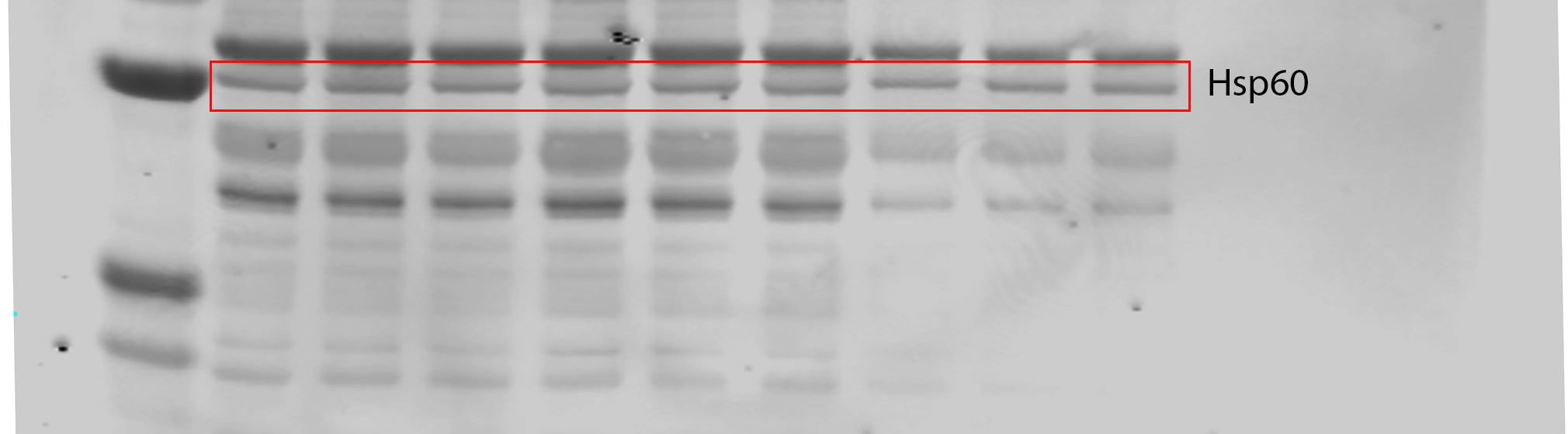

Supplement: Supplementary file 8 — Source Data for Figure 1 [file EMMM-15-e17463-s009.zip › Figure 1/1C/HSP60.tif]

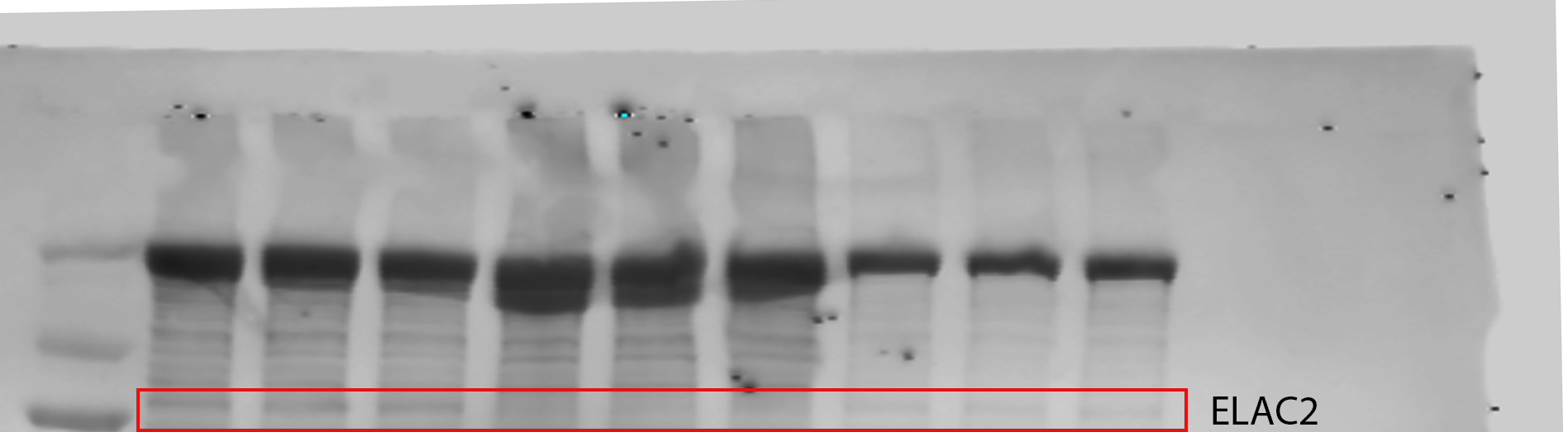

Supplement: Supplementary file 8 — Source Data for Figure 1 [file EMMM-15-e17463-s009.zip › Figure 1/1C/ELAC2.tif]

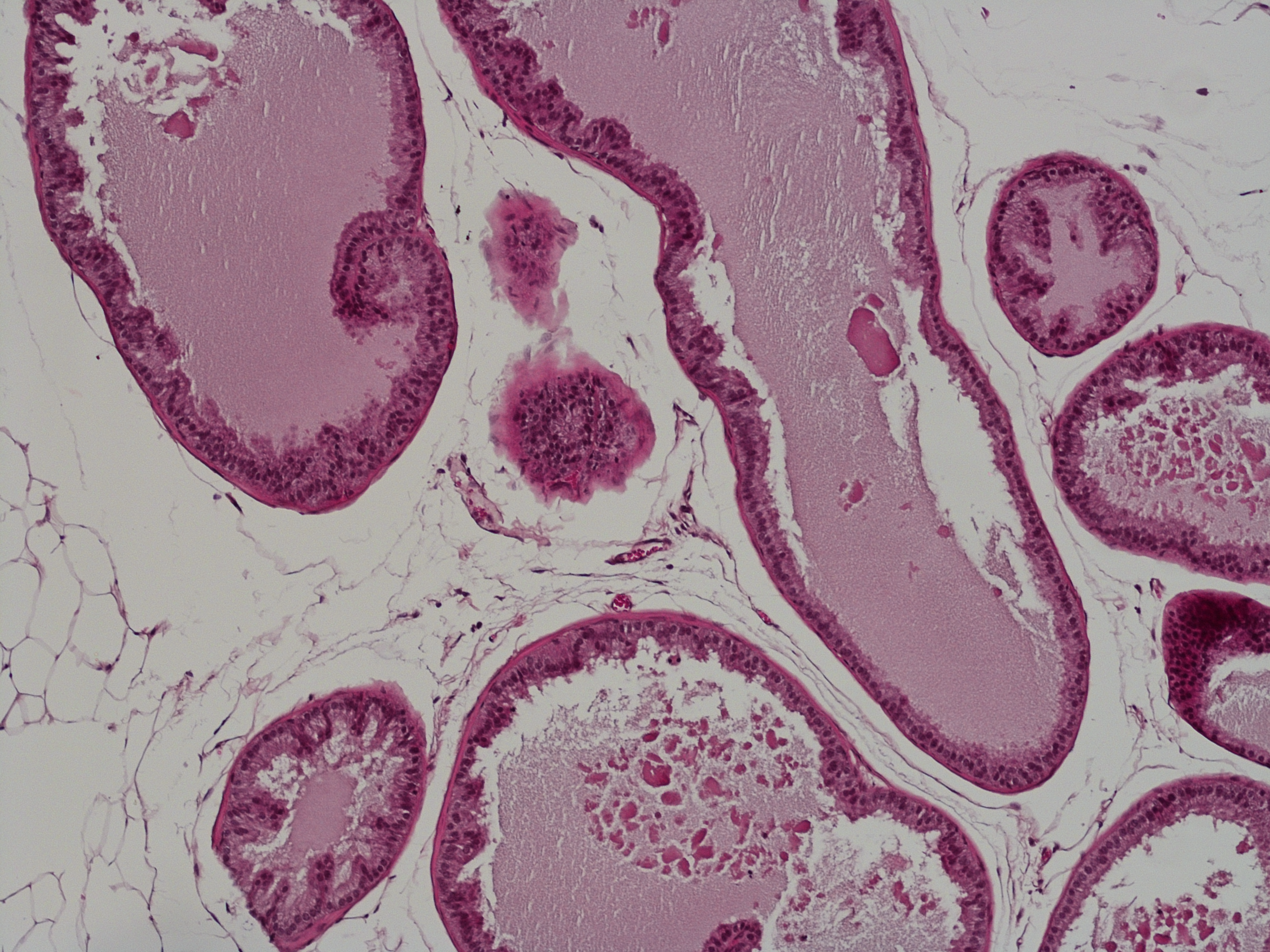

Supplement: Supplementary file 9 — Source Data for Figure 2 [file EMMM-15-e17463-s005.zip › Figure 2/2A/Ventral lobe A537T.tif]

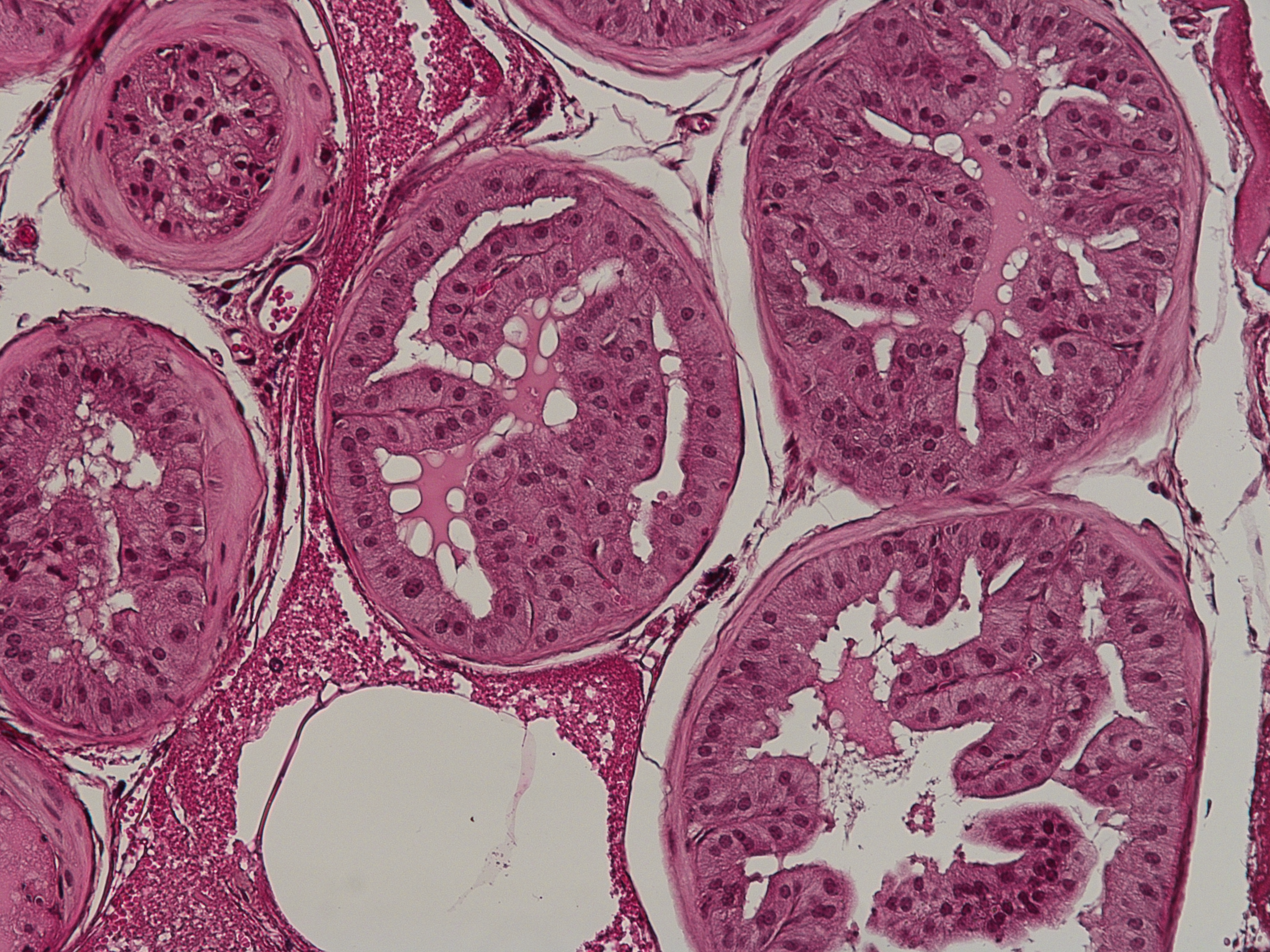

Supplement: Supplementary file 9 — Source Data for Figure 2 [file EMMM-15-e17463-s005.zip › Figure 2/2A/Dorsal lobe KO.tif]

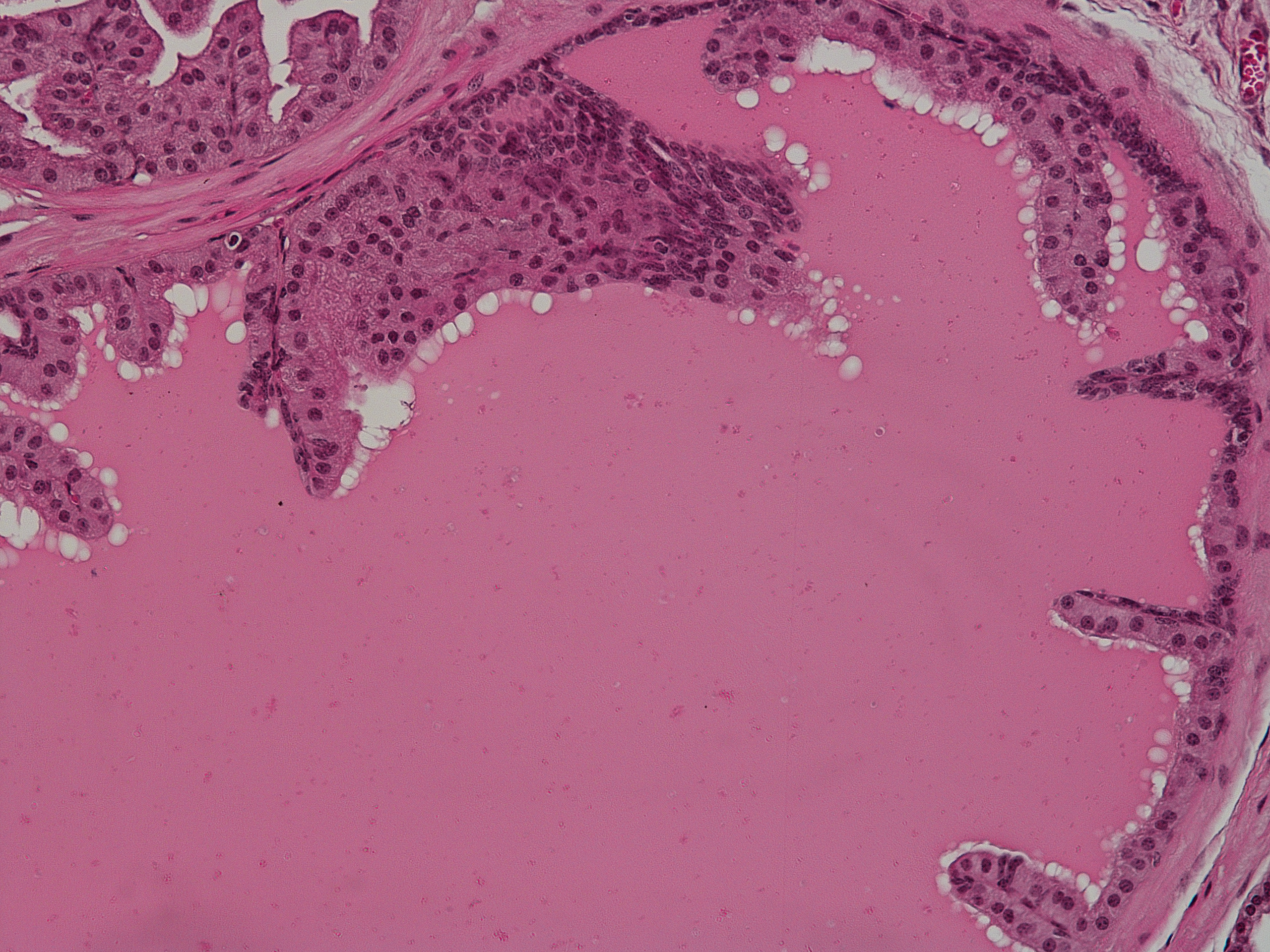

Supplement: Supplementary file 9 — Source Data for Figure 2 [file EMMM-15-e17463-s005.zip › Figure 2/2A/Anterior lobe WT.tif]

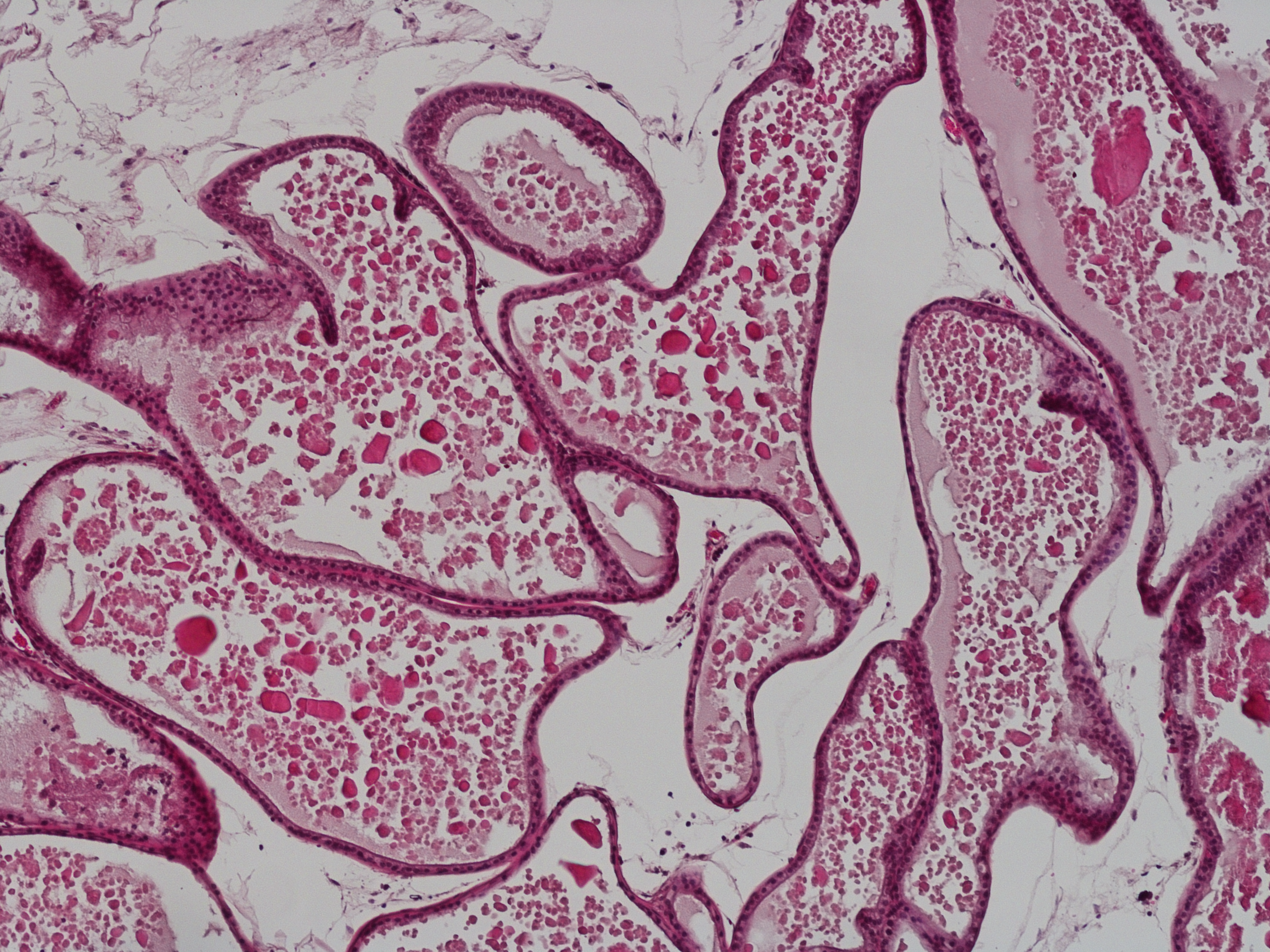

Supplement: Supplementary file 9 — Source Data for Figure 2 [file EMMM-15-e17463-s005.zip › Figure 2/2A/Lateral lobe WT.tif]

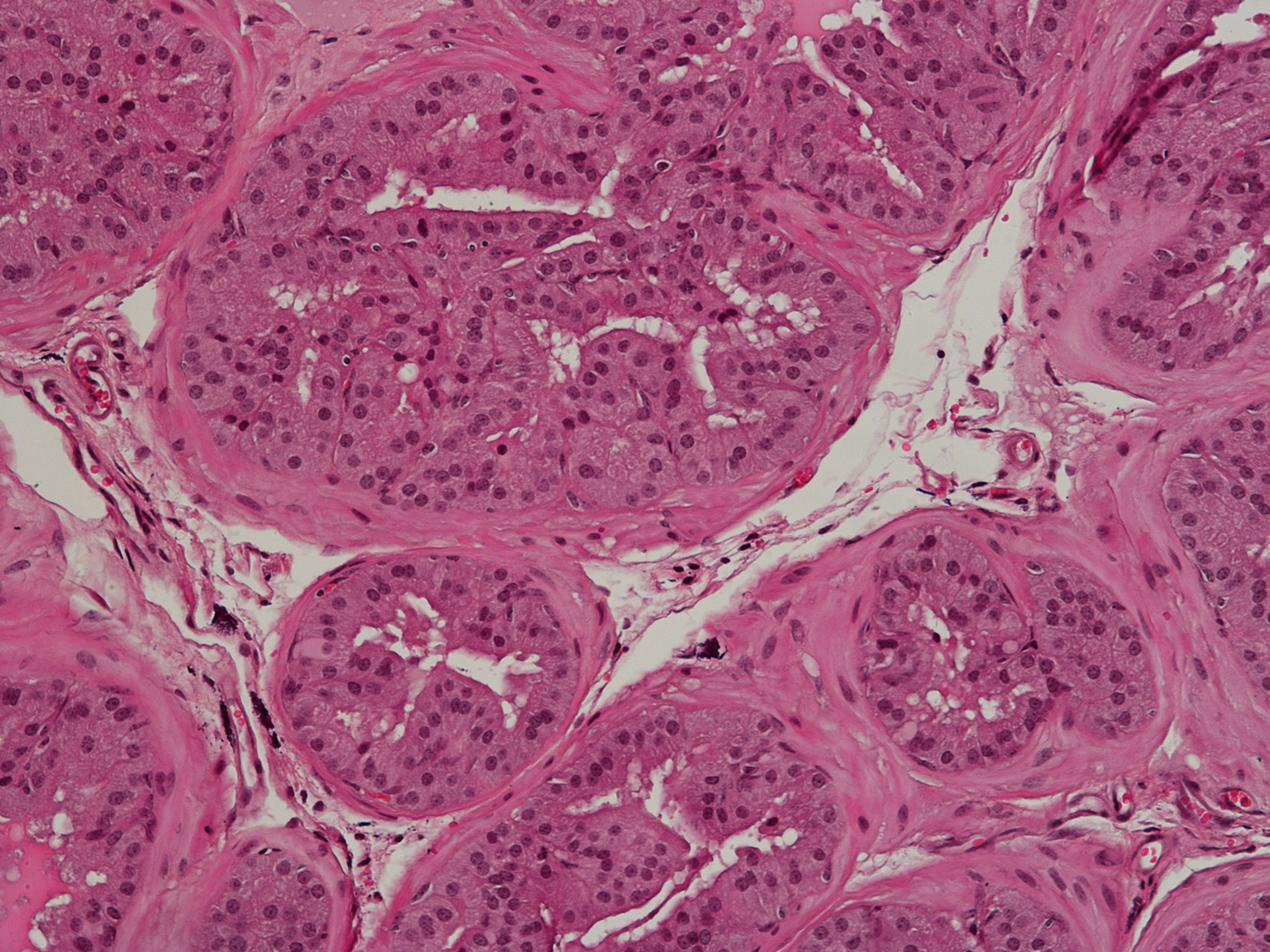

Supplement: Supplementary file 9 — Source Data for Figure 2 [file EMMM-15-e17463-s005.zip › Figure 2/2A/Dorsal lobe A537T.tif]

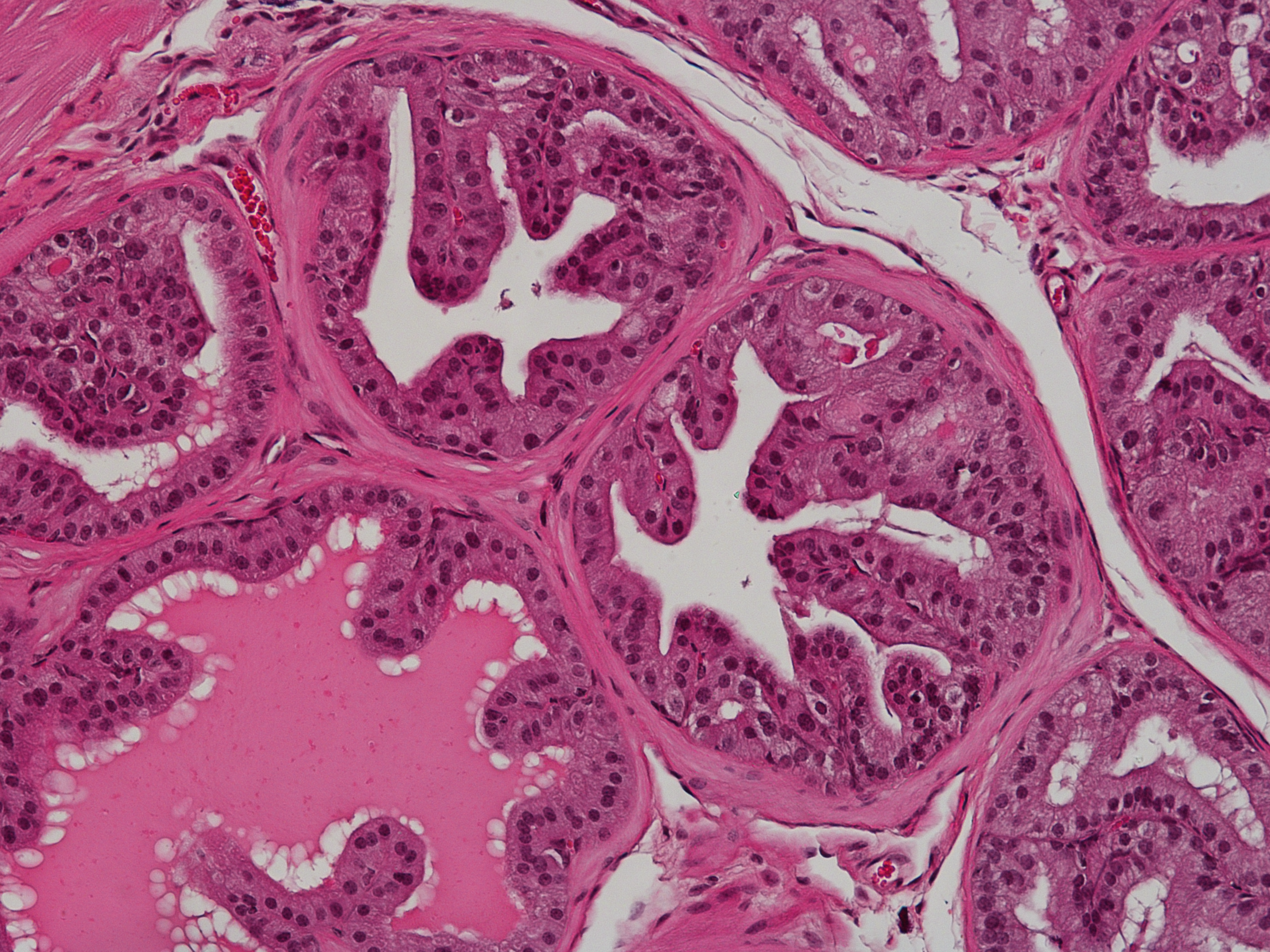

Supplement: Supplementary file 9 — Source Data for Figure 2 [file EMMM-15-e17463-s005.zip › Figure 2/2A/Dorsal lobe WT.tif]

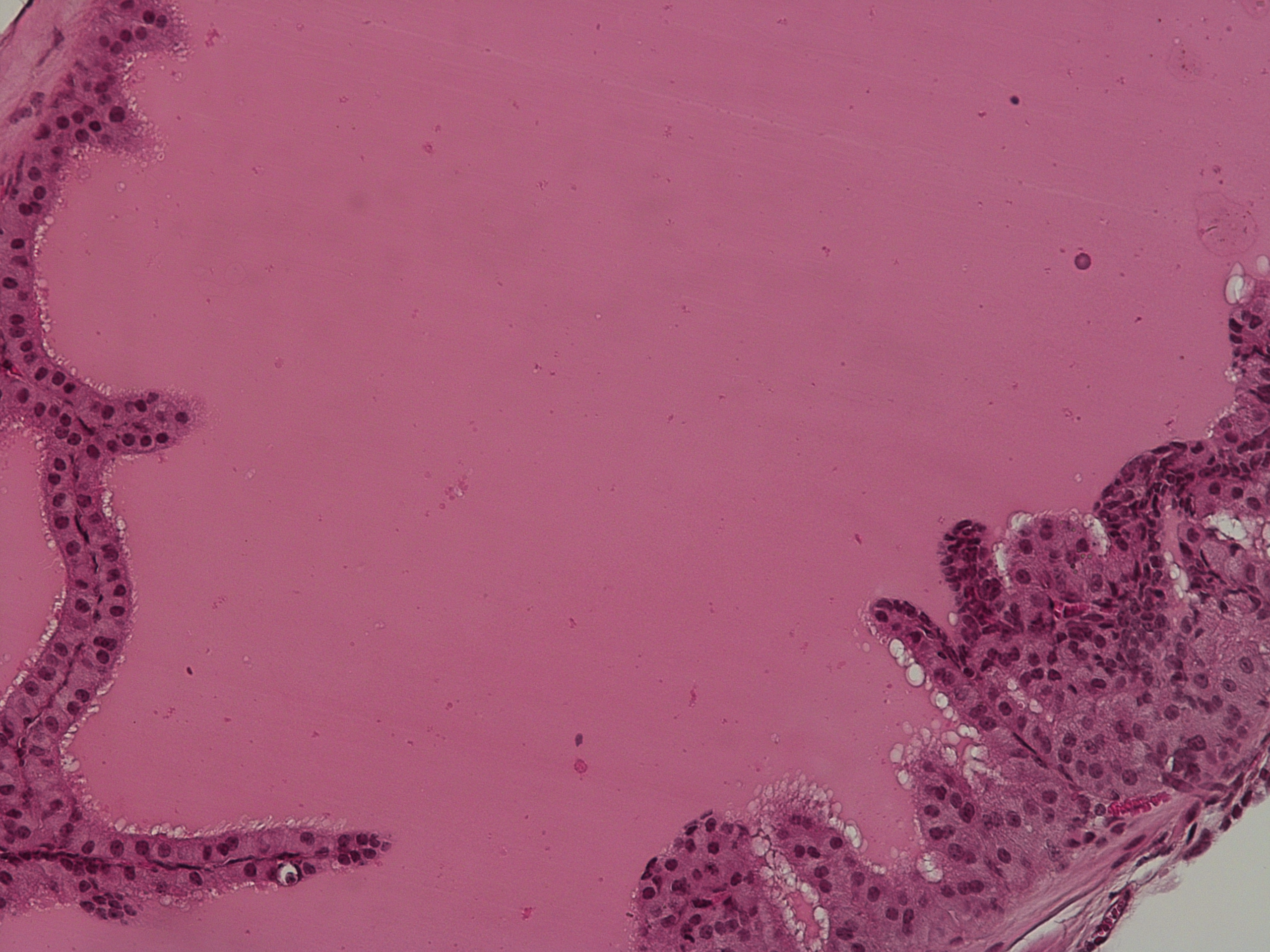

Supplement: Supplementary file 9 — Source Data for Figure 2 [file EMMM-15-e17463-s005.zip › Figure 2/2A/Anterior lobe KO.tif]

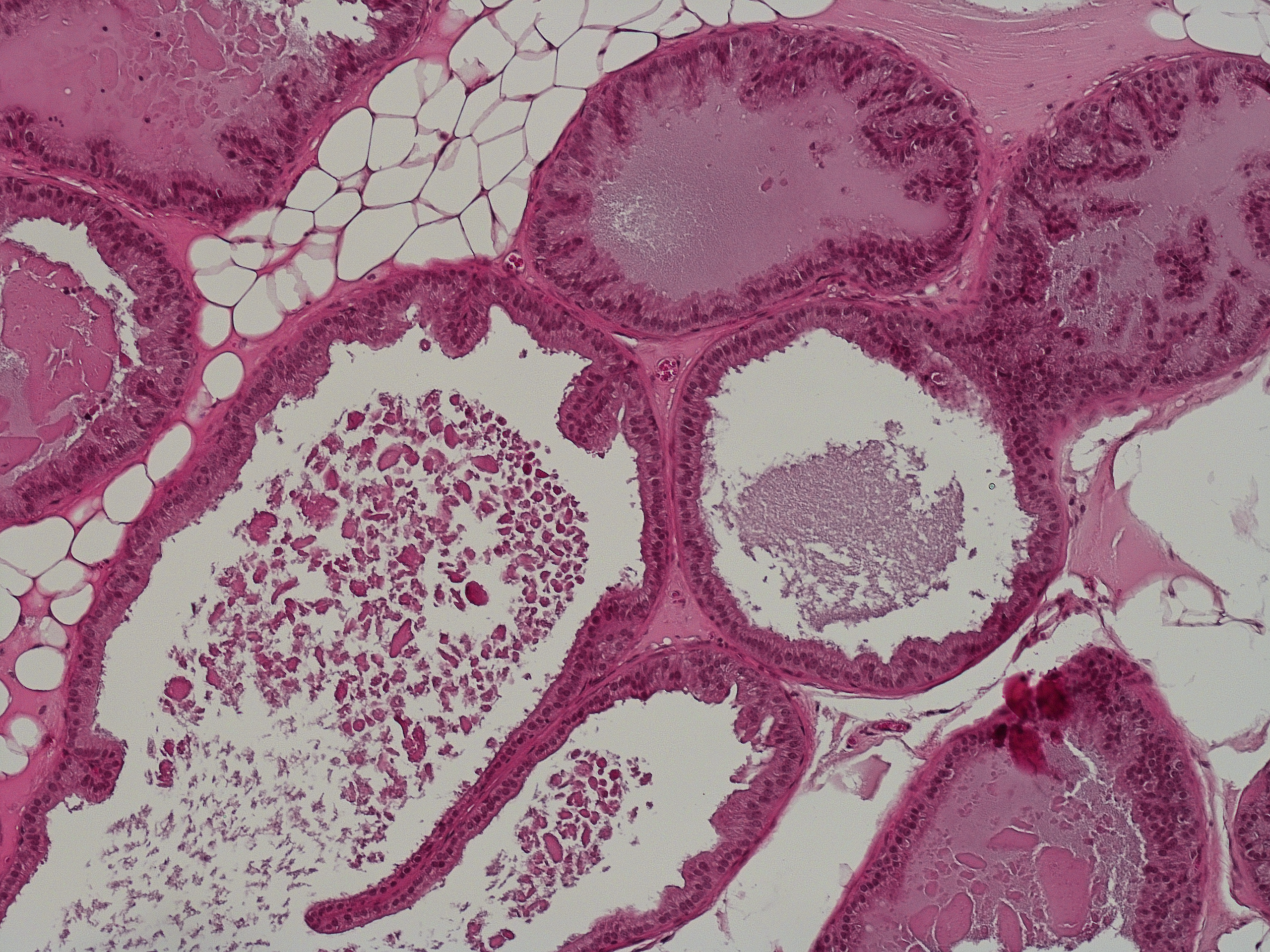

Supplement: Supplementary file 9 — Source Data for Figure 2 [file EMMM-15-e17463-s005.zip › Figure 2/2A/Lateral lobe KO.tif]

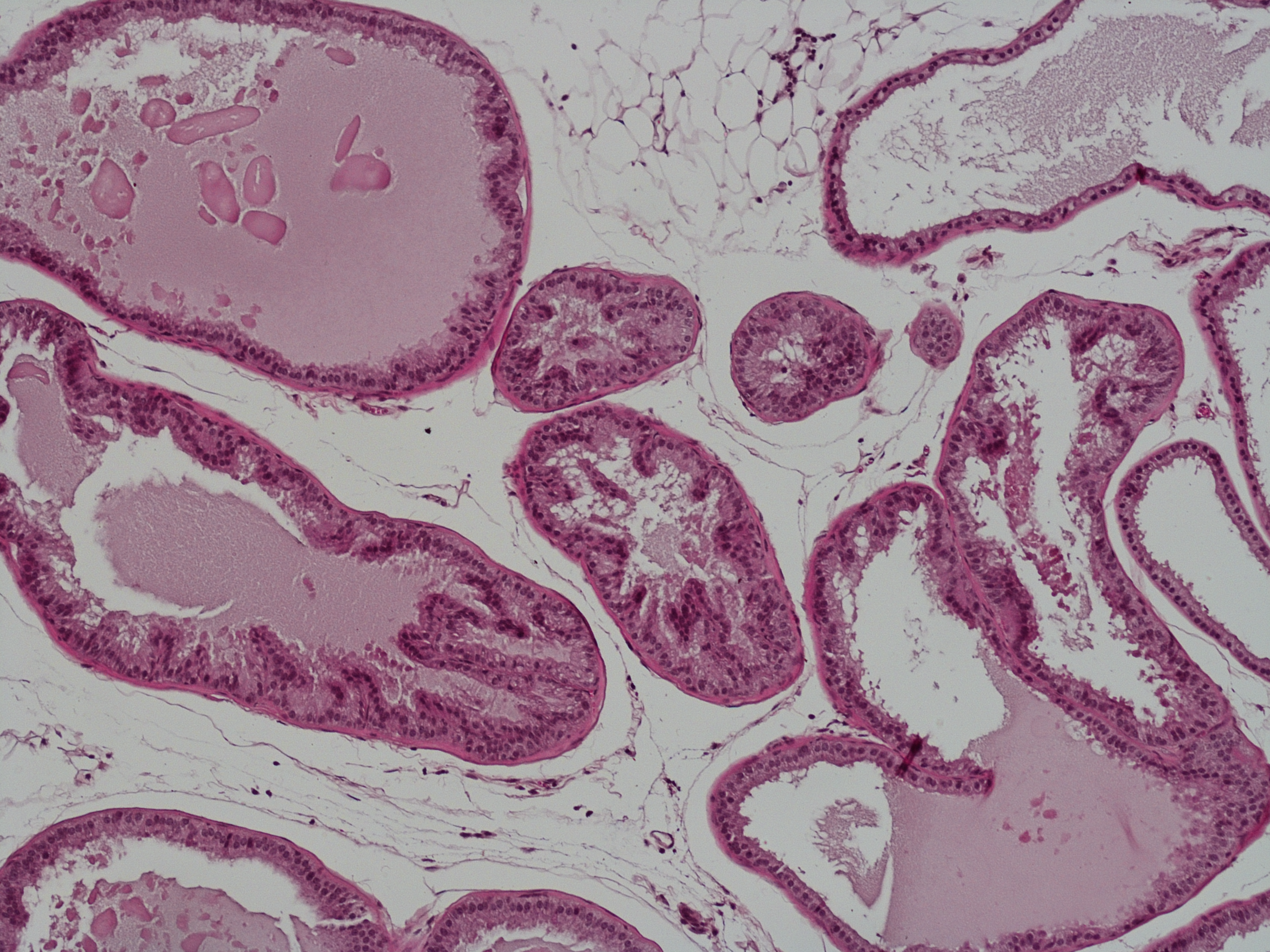

Supplement: Supplementary file 9 — Source Data for Figure 2 [file EMMM-15-e17463-s005.zip › Figure 2/2A/Ventral lobe WT.tif]

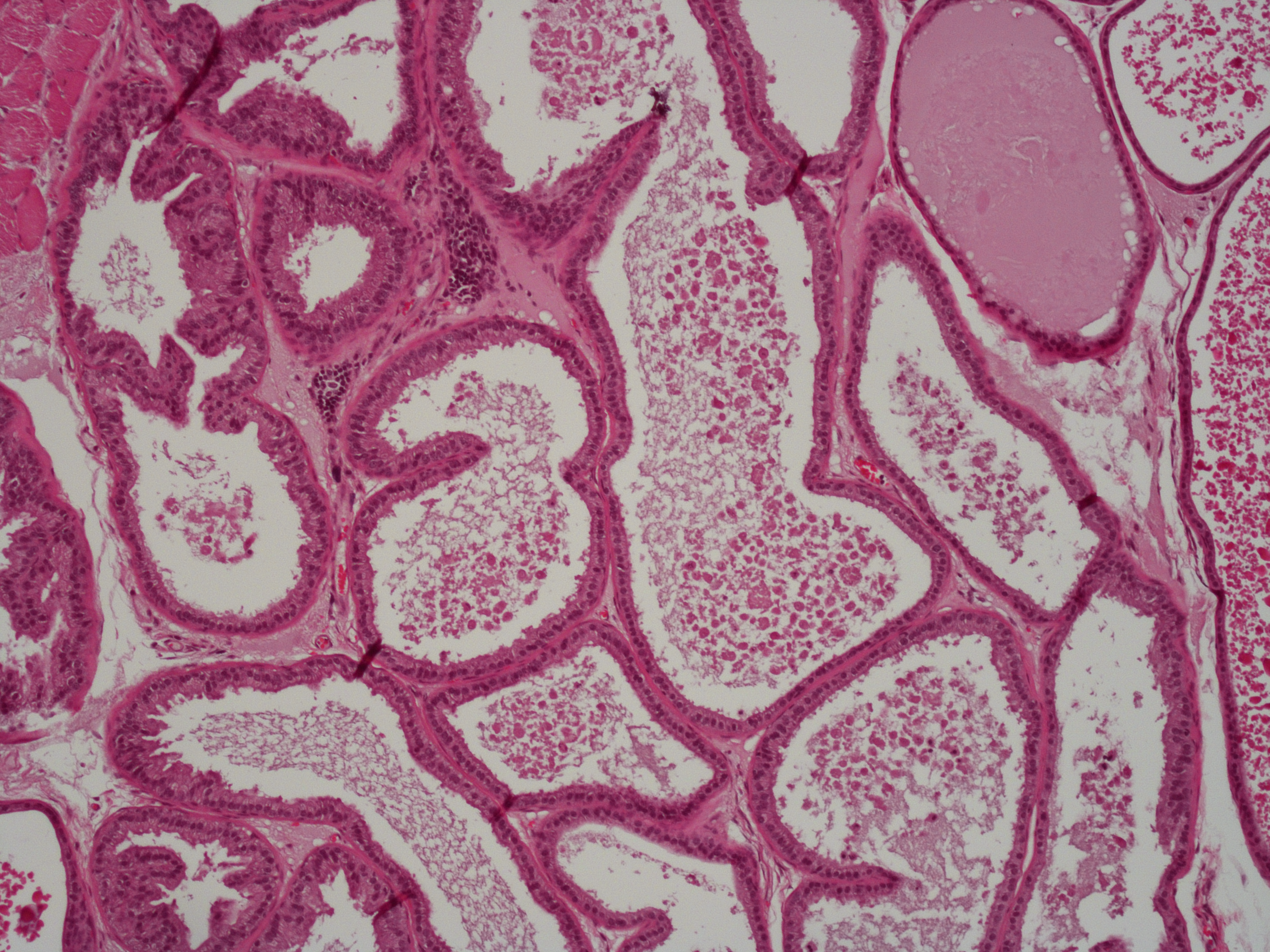

Supplement: Supplementary file 9 — Source Data for Figure 2 [file EMMM-15-e17463-s005.zip › Figure 2/2A/Lateral lobe A537T.tif]

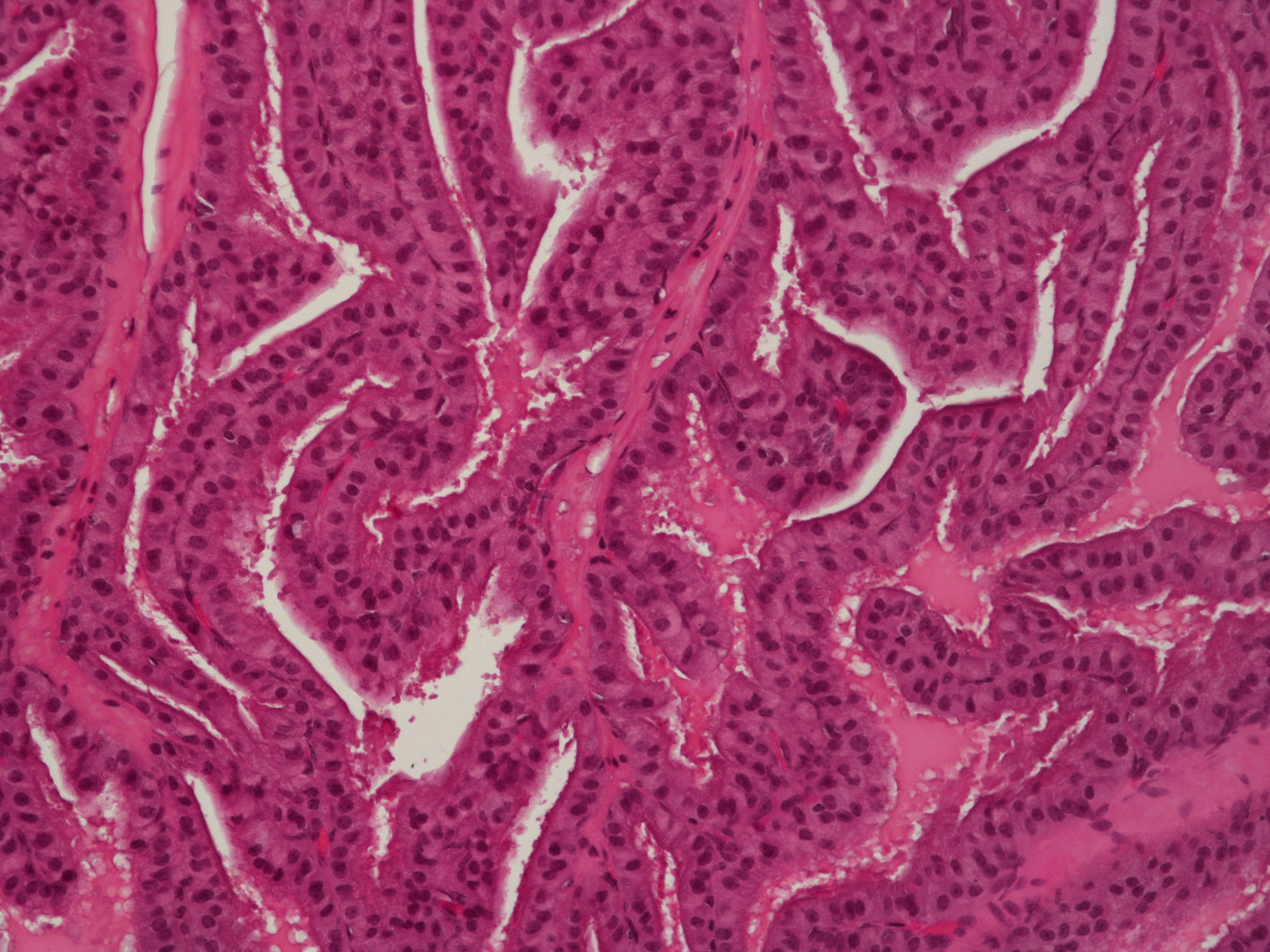

Supplement: Supplementary file 9 — Source Data for Figure 2 [file EMMM-15-e17463-s005.zip › Figure 2/2A/Anterior lobe A537T.tif]

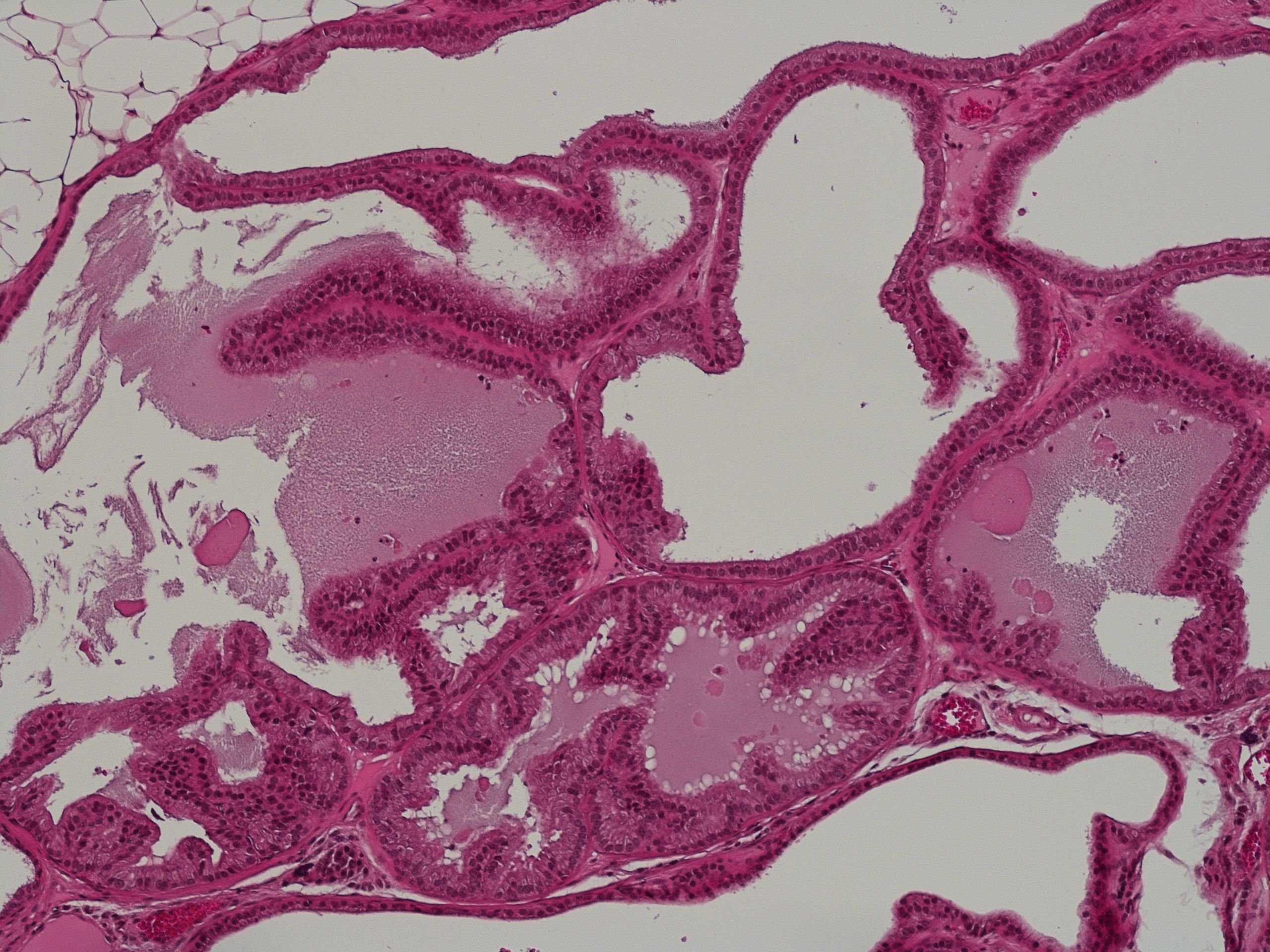

Supplement: Supplementary file 9 — Source Data for Figure 2 [file EMMM-15-e17463-s005.zip › Figure 2/2A/Ventral lobe KO.tif]

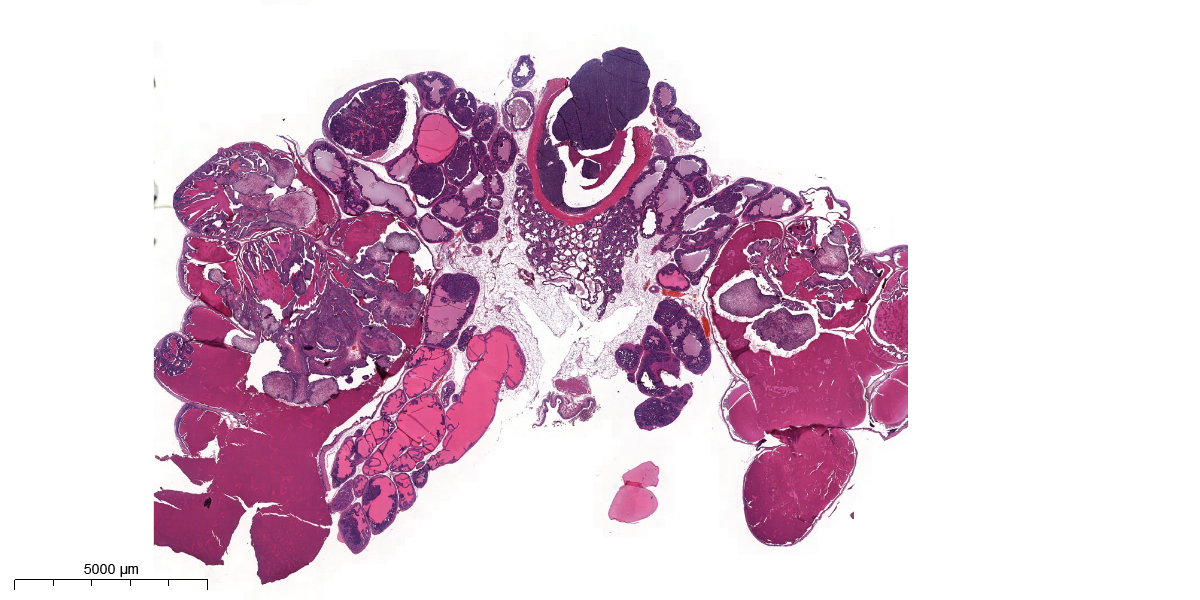

Supplement: Supplementary file 10 — Source Data for Figure 3 [file EMMM-15-e17463-s011.zip › Figure 3/3C/TRAMP.tif]

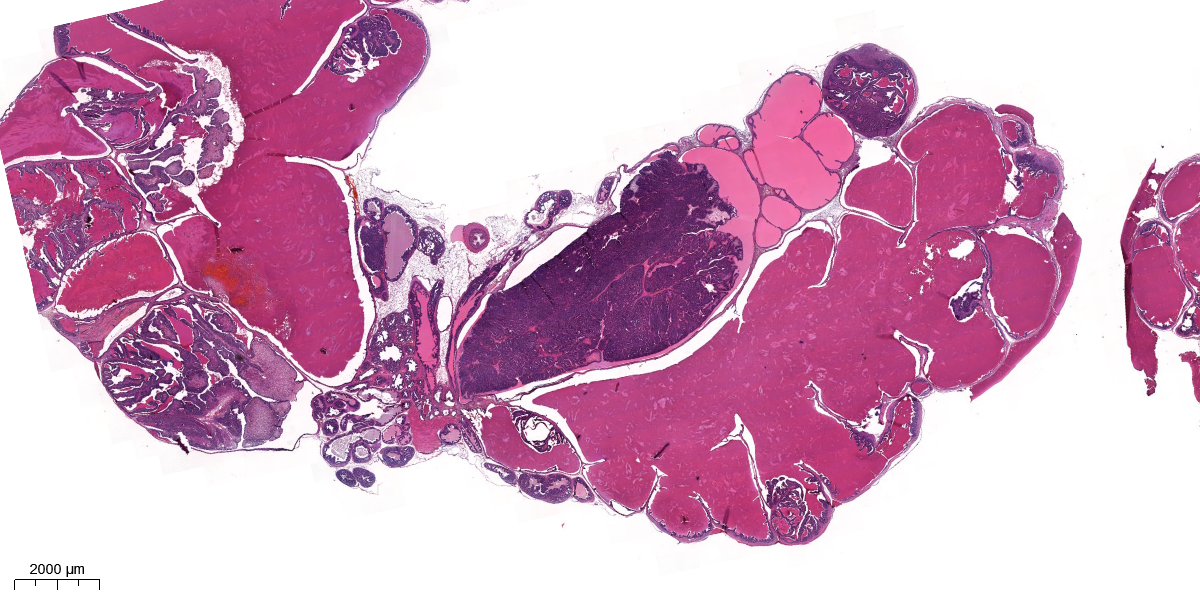

Supplement: Supplementary file 10 — Source Data for Figure 3 [file EMMM-15-e17463-s011.zip › Figure 3/3C/A537T-TRAMP.tif]

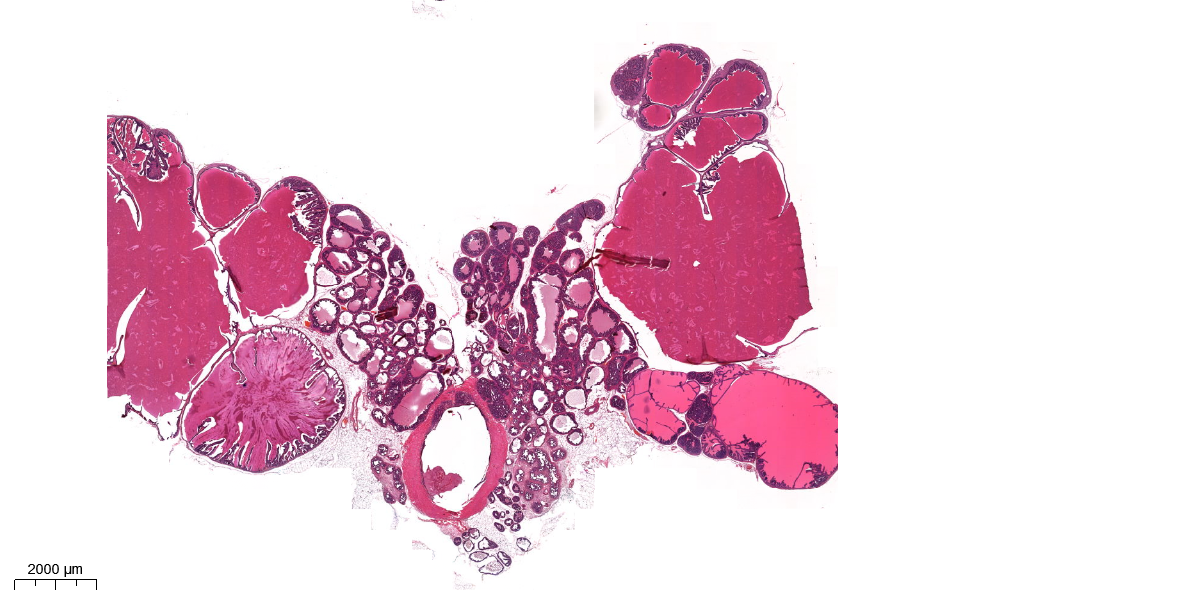

Supplement: Supplementary file 10 — Source Data for Figure 3 [file EMMM-15-e17463-s011.zip › Figure 3/3C/TRAMP-KO.tif]

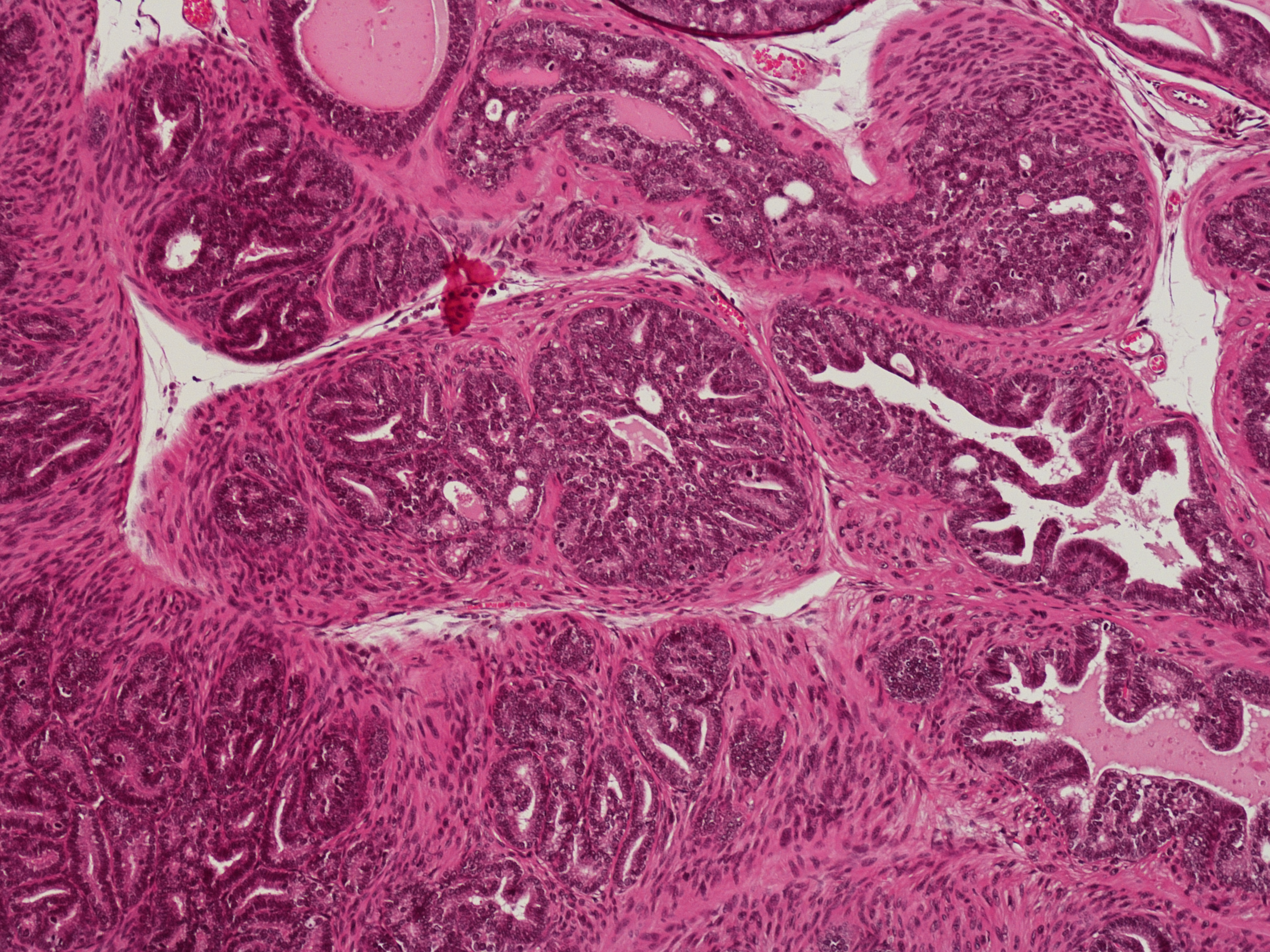

Supplement: Supplementary file 10 — Source Data for Figure 3 [file EMMM-15-e17463-s011.zip › Figure 3/3A/Dorsal lobe TRAMP.tif]

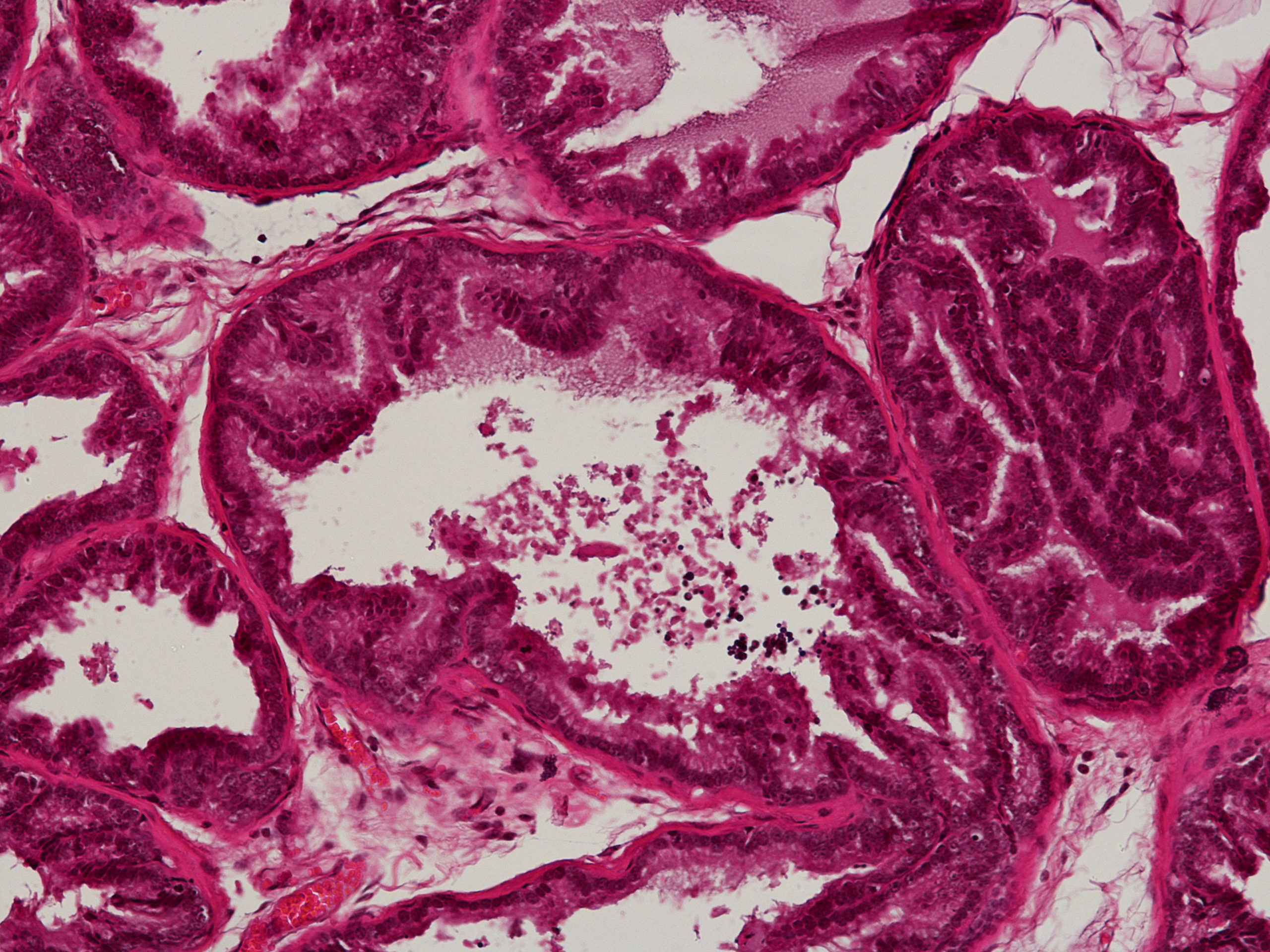

Supplement: Supplementary file 10 — Source Data for Figure 3 [file EMMM-15-e17463-s011.zip › Figure 3/3A/Ventral lobe TRAMP.tif]

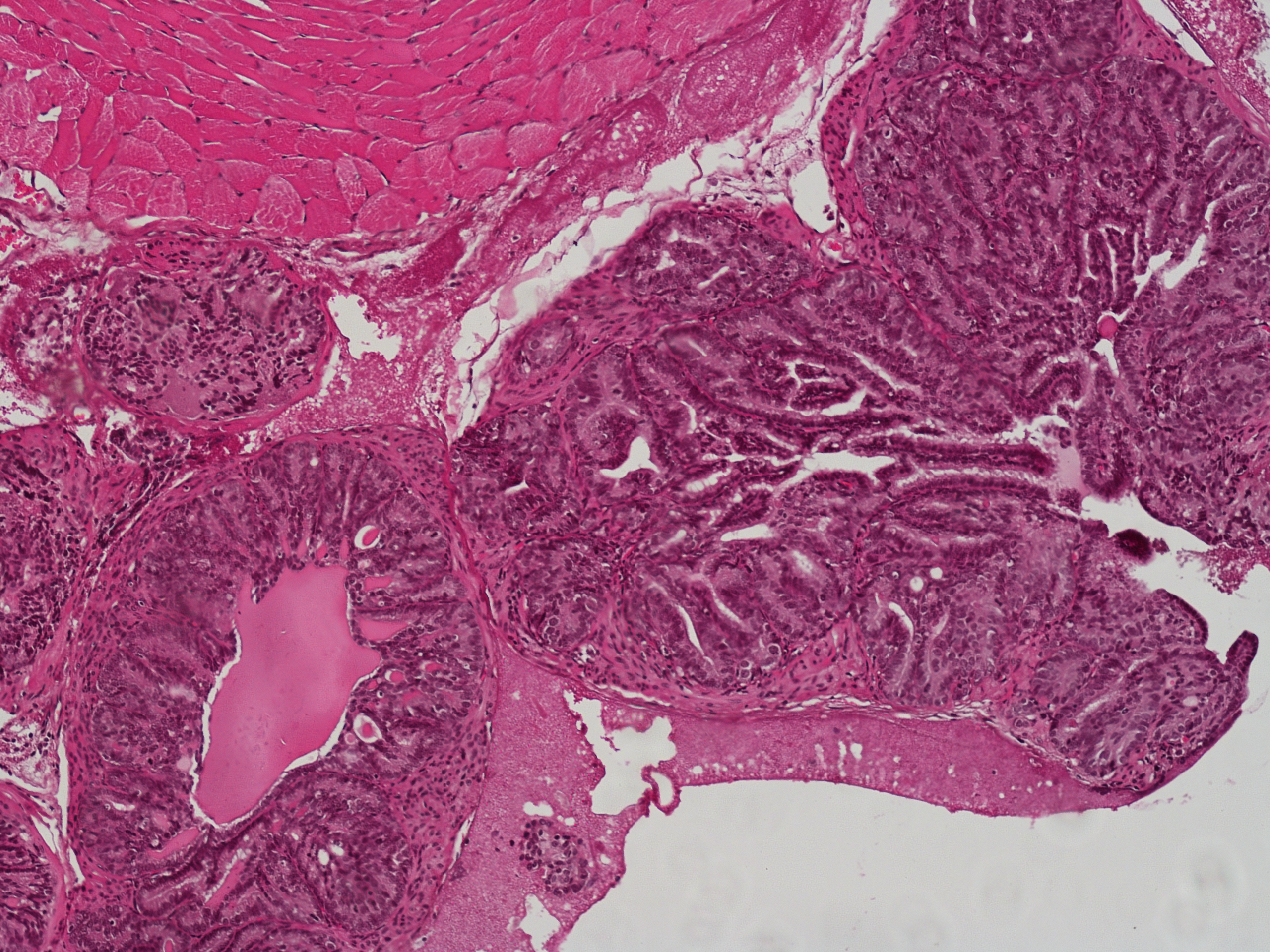

Supplement: Supplementary file 10 — Source Data for Figure 3 [file EMMM-15-e17463-s011.zip › Figure 3/3A/Dorsal lobe A537T-TRAMP.tif]

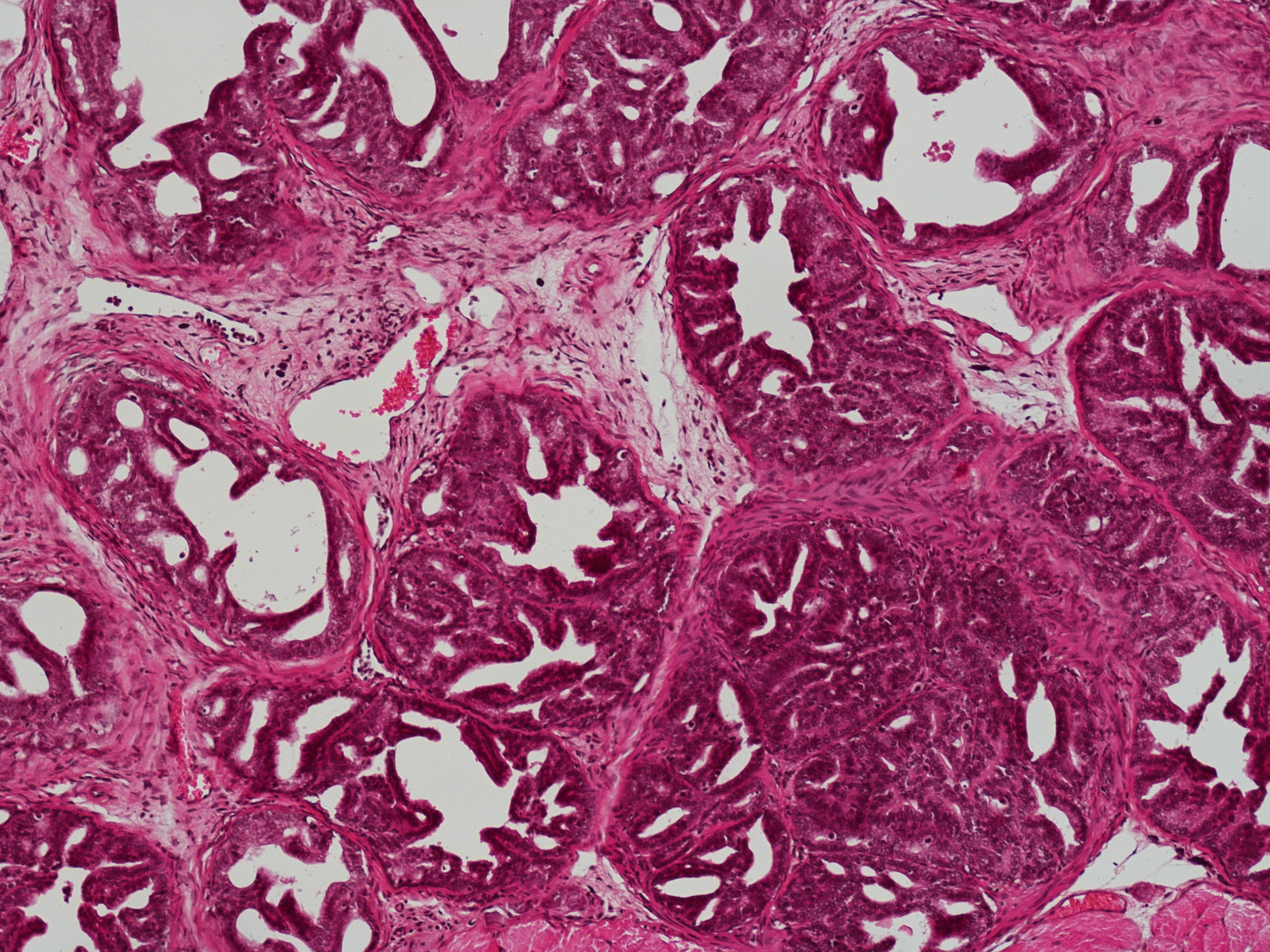

Supplement: Supplementary file 10 — Source Data for Figure 3 [file EMMM-15-e17463-s011.zip › Figure 3/3A/Lateral lobe KO-TRAMP.tif]

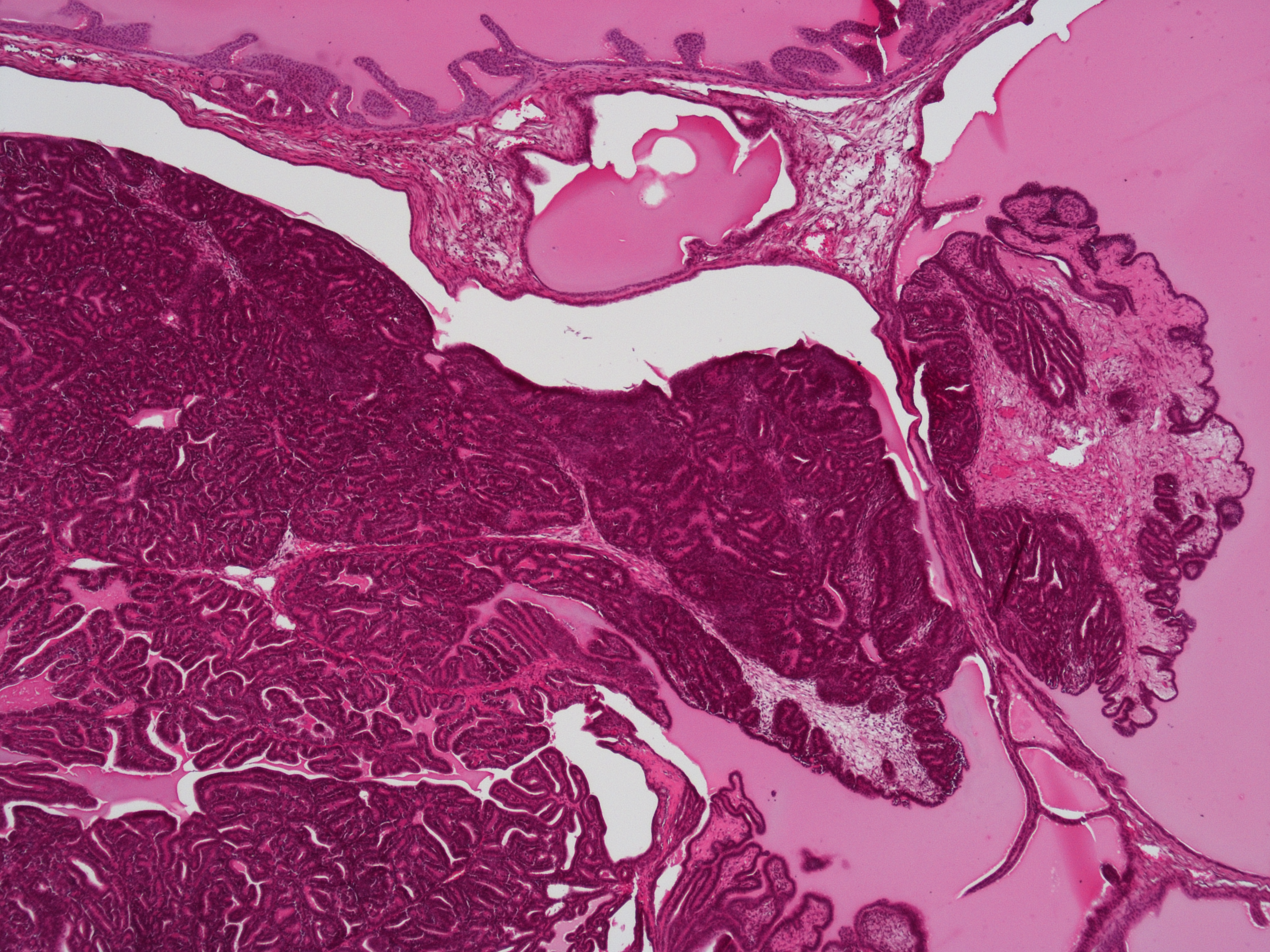

Supplement: Supplementary file 10 — Source Data for Figure 3 [file EMMM-15-e17463-s011.zip › Figure 3/3A/Anterior lobe A537T-TRAMP.tif]

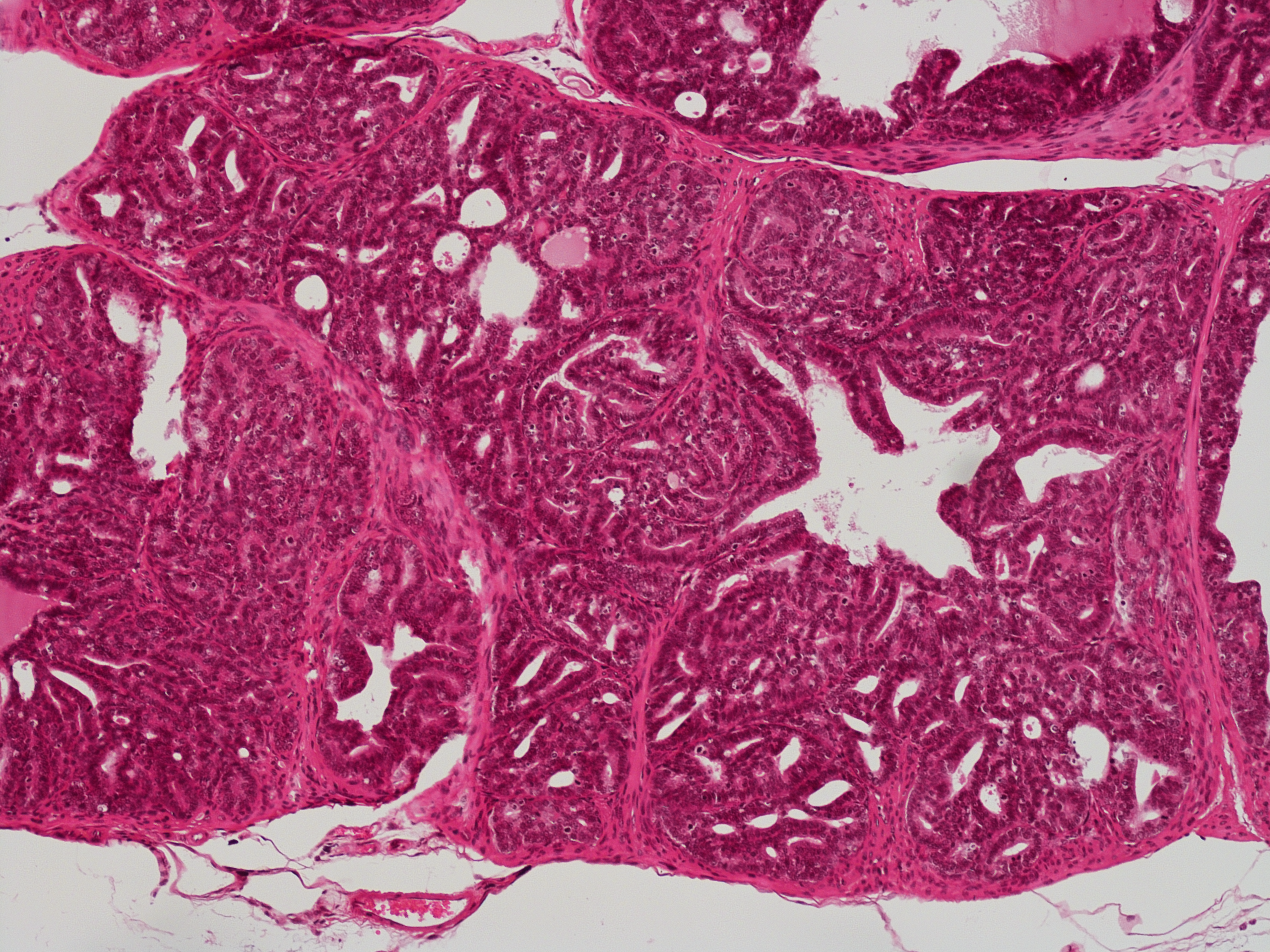

Supplement: Supplementary file 10 — Source Data for Figure 3 [file EMMM-15-e17463-s011.zip › Figure 3/3A/Dorsal lobe KO-TRAMP.tif]

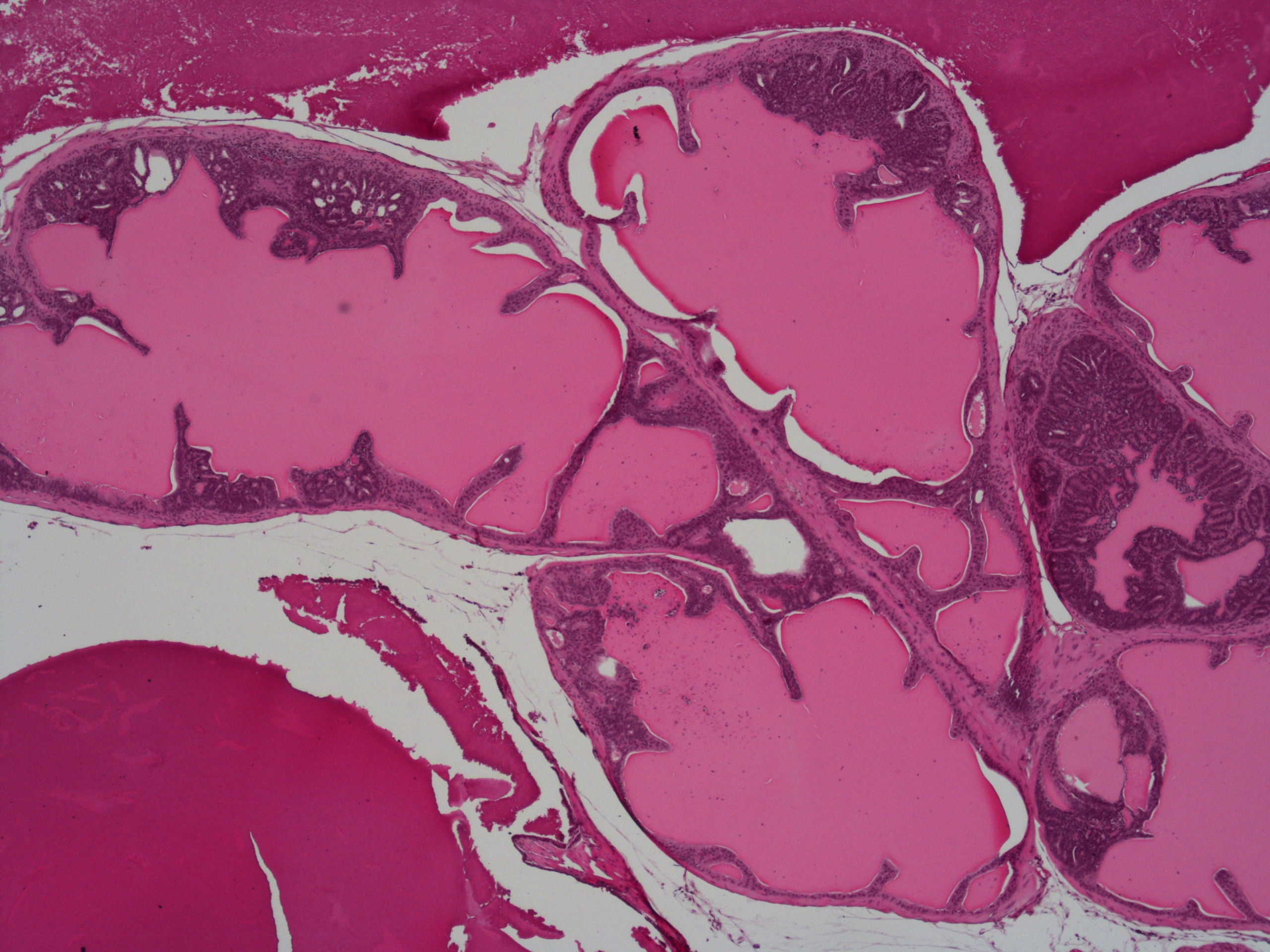

Supplement: Supplementary file 10 — Source Data for Figure 3 [file EMMM-15-e17463-s011.zip › Figure 3/3A/Anterior lobe KO-TRAMP.tif]

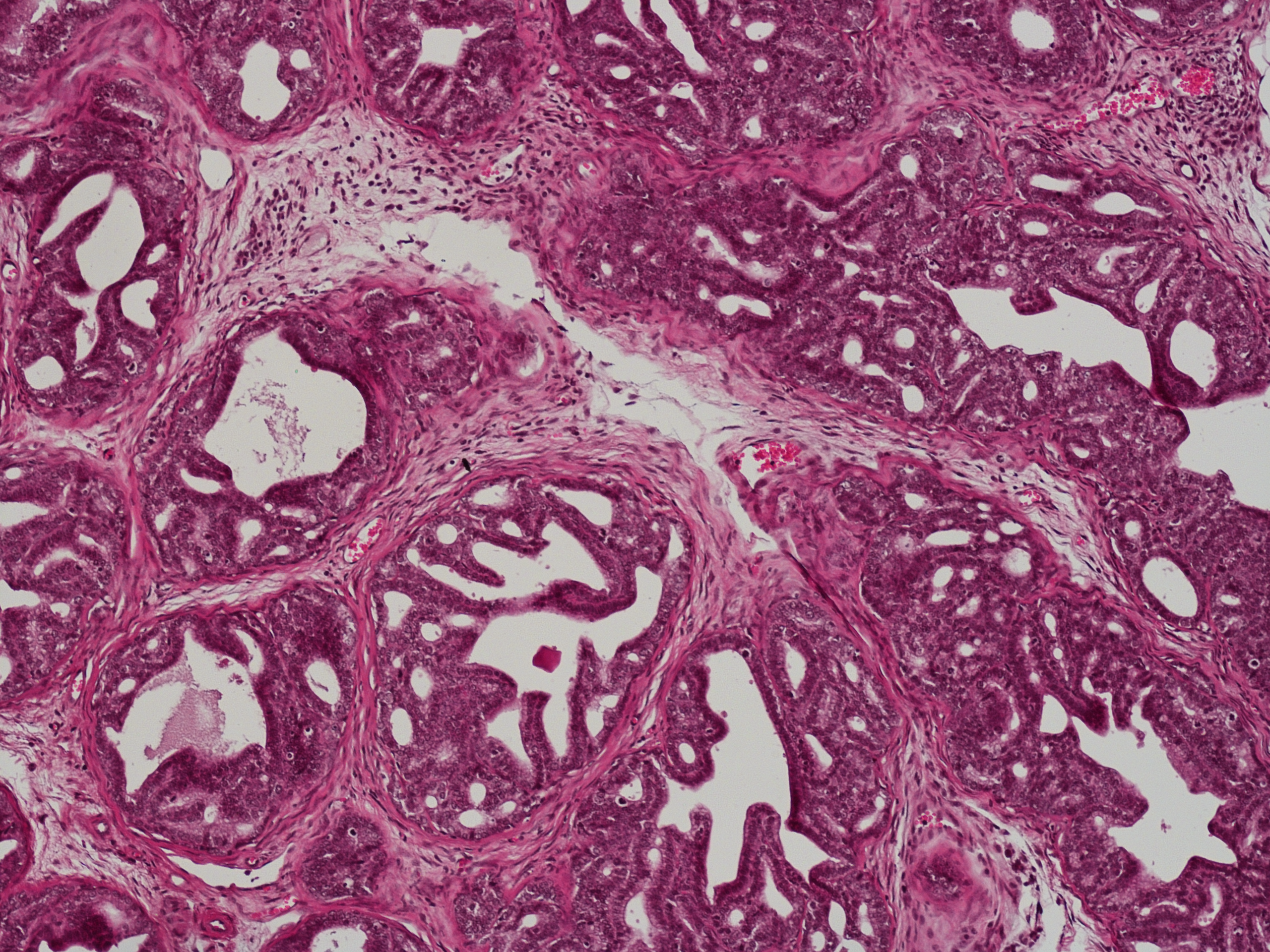

Supplement: Supplementary file 10 — Source Data for Figure 3 [file EMMM-15-e17463-s011.zip › Figure 3/3A/Lateral lobe A537T-TRAMP.tif]

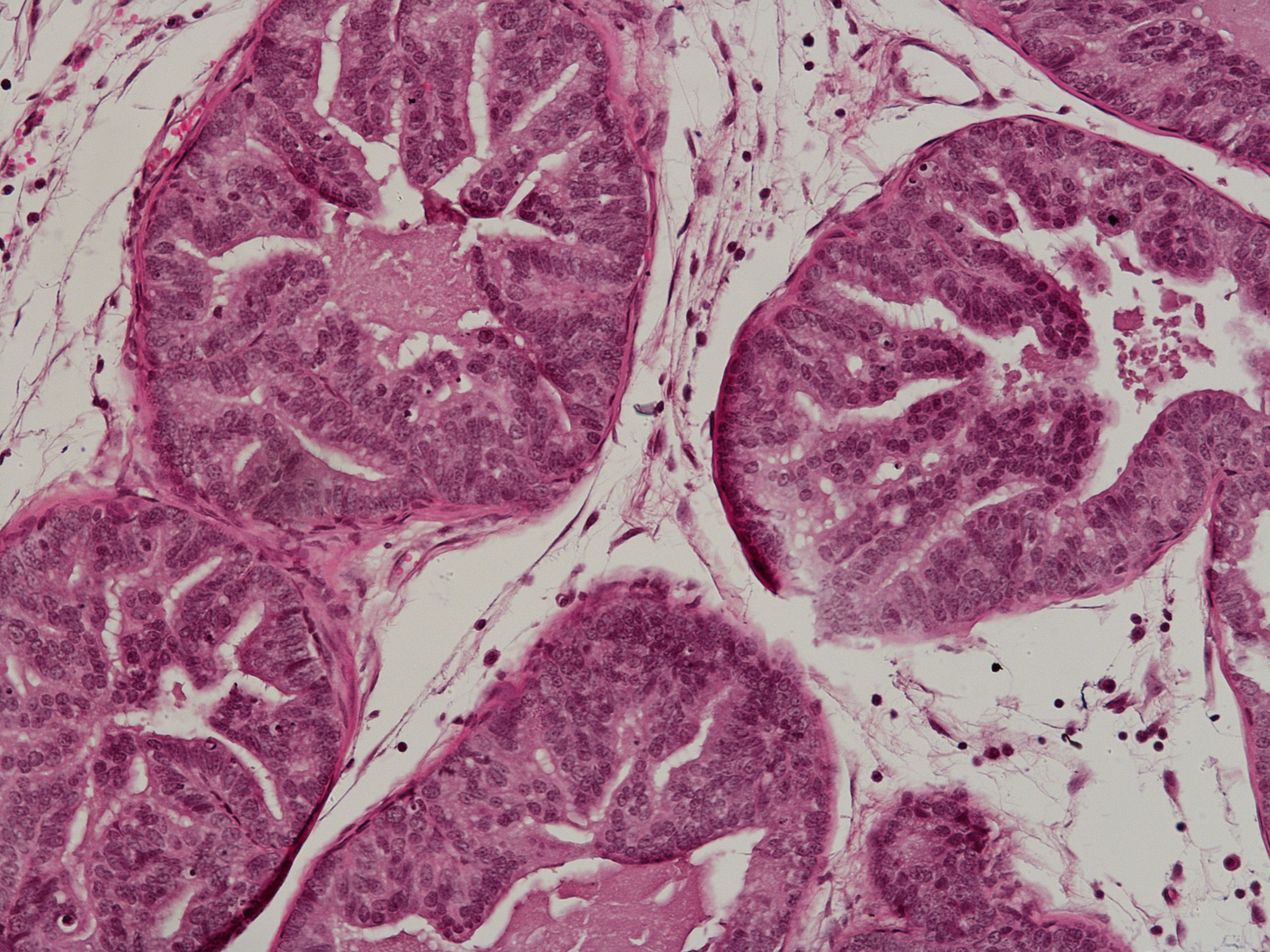

Supplement: Supplementary file 10 — Source Data for Figure 3 [file EMMM-15-e17463-s011.zip › Figure 3/3A/Ventral lobe A537T-TRAMP.tif]

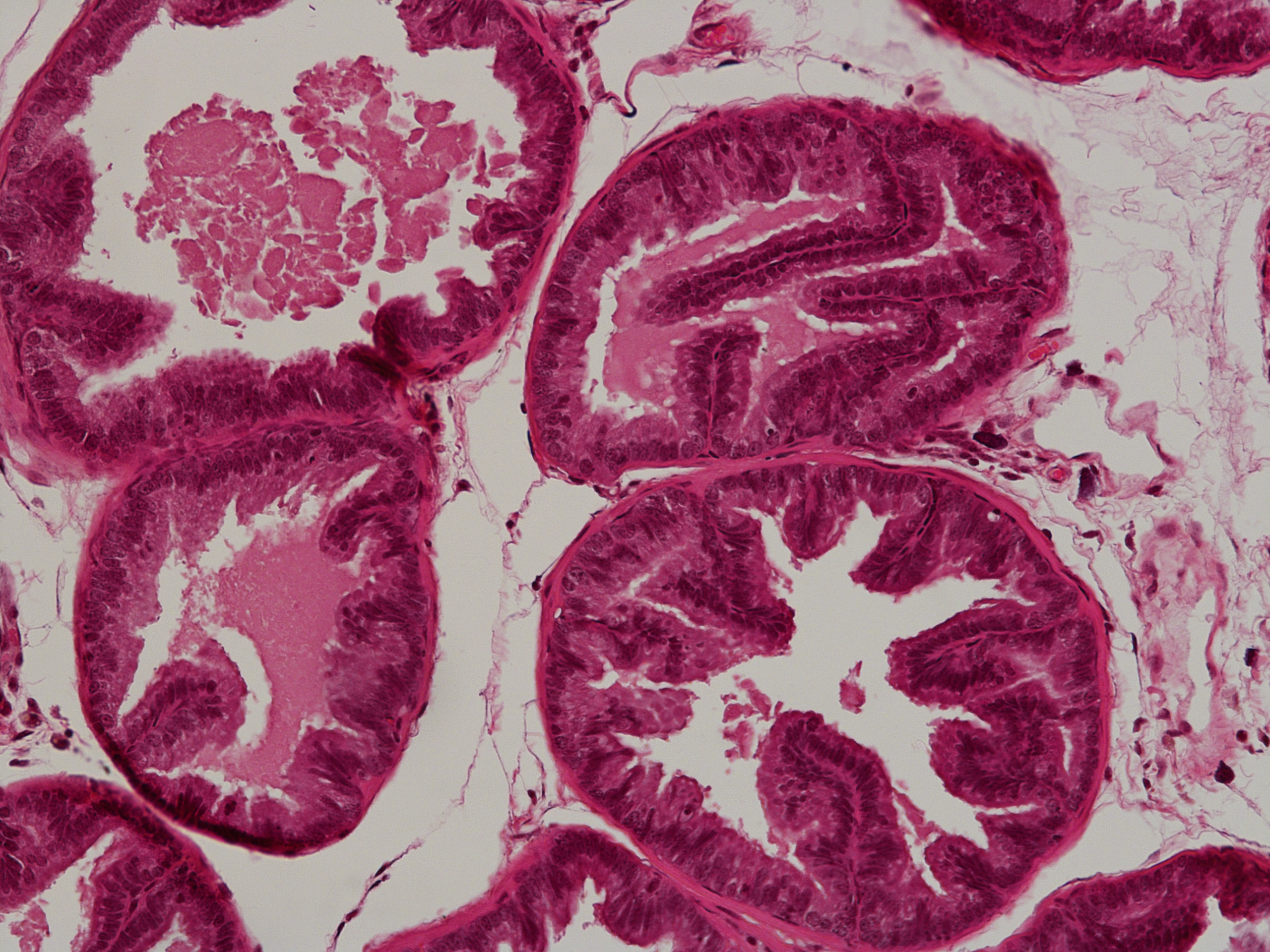

Supplement: Supplementary file 10 — Source Data for Figure 3 [file EMMM-15-e17463-s011.zip › Figure 3/3A/Ventral lobe KO-TRAMP.tif]

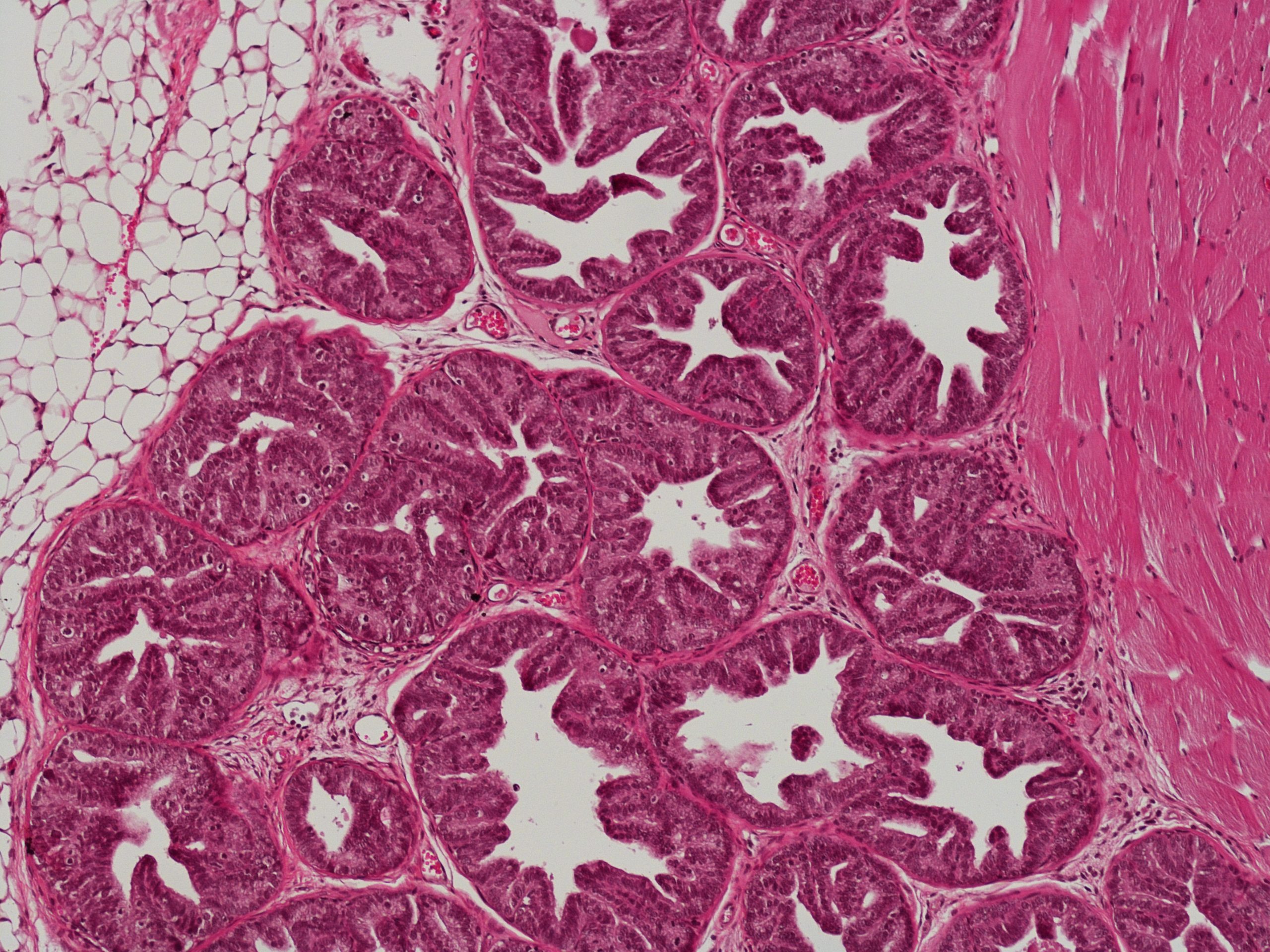

Supplement: Supplementary file 10 — Source Data for Figure 3 [file EMMM-15-e17463-s011.zip › Figure 3/3A/Lateral lobe TRAMP.tif]

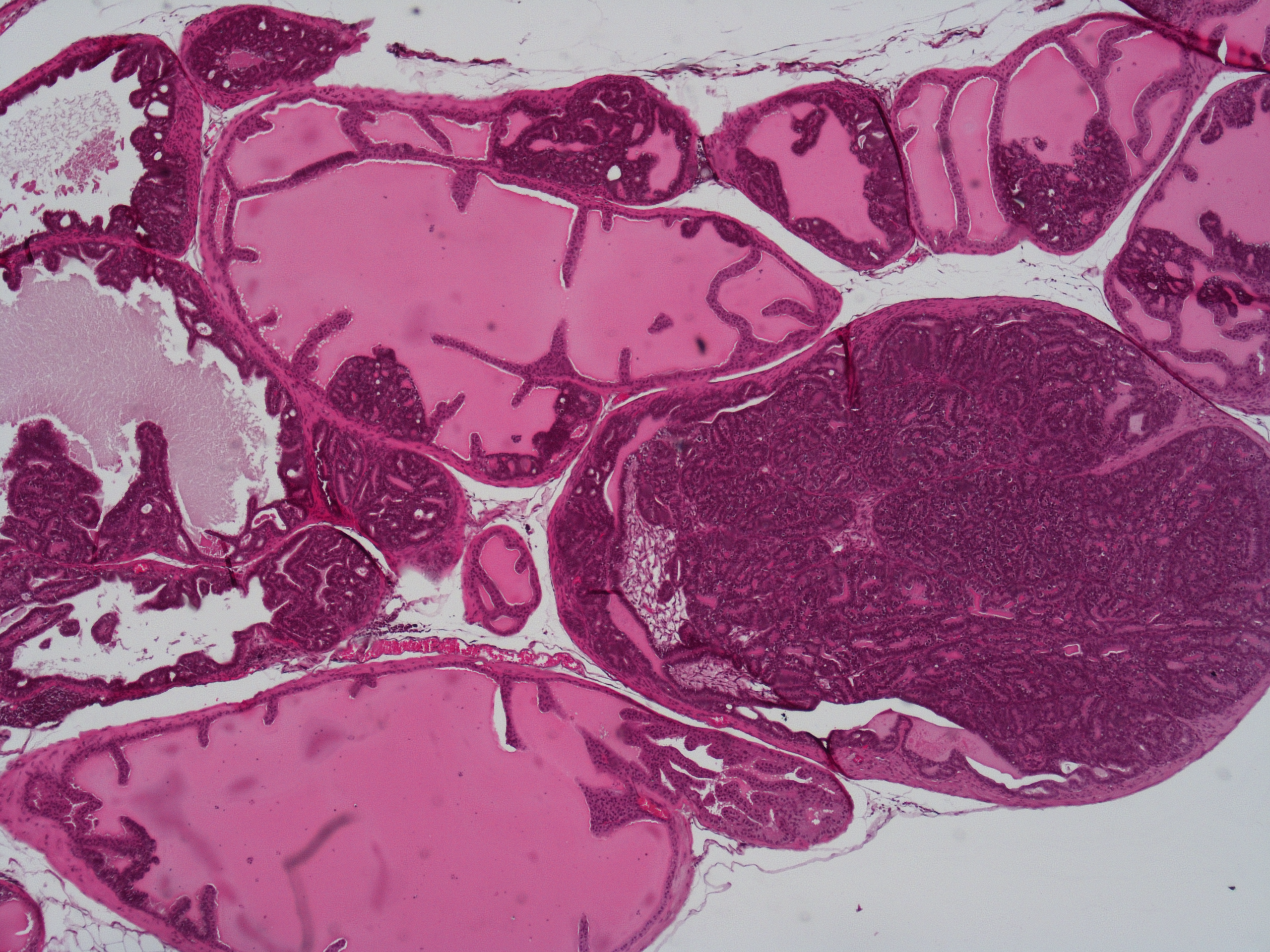

Supplement: Supplementary file 10 — Source Data for Figure 3 [file EMMM-15-e17463-s011.zip › Figure 3/3A/Anterior lobe TRAMP.tif]
