# Supplementary material for: Multi‐omic profiling reveals an RNA processing rheostat that predisposes to prostate cancer
Source: EMBO Mol Med. 2023 Apr 24;15(6):e17463. doi: 10.15252/emmm.202317463 (PMC10245041; doi:10.15252/emmm.202317463)
Supplement: Supplementary file 11 [file EMMM-15-e17463-s007.pdf]

EMBO Molecular Medicine

Multi-omic profiling reveals an RNA processing rheostat that predisposes to prostate cancer

Maike Stentenbach, Judith Ermer, Danielle Rudler, Kara Perks, Samuel Raven, Richard Lee, Tim McCubbin, Esteban Marcellin, Stefan Siira, Oliver Rackham, and Aleksandra Filipovska
DOI: 10.15252/emmm.202317463

Corresponding author(s): Aleksandra Filipovska (aleksandra.filipovska@uwa.edu.au)

Review Timeline:

Submission Date:	20th Jan 23
Editorial Decision:	13th Mar 23
Revision Received:	22nd Mar 23
Editorial Decision:	30th Mar 23
Revision Received:	30th Mar 23
Accepted:	31st Mar 23

Editor: Lise Roth

Transaction Report:

(Note: With the exception of the correction of typographical or spelling errors that could be a source of ambiguity, letters and reports are not edited. Depending on transfer agreements, referee reports obtained elsewhere may or may not be included in this compilation. Referee reports are anonymous unless the Referee chooses to sign their reports.)

13th Mar 2023

Dear Prof. Filipovska,

Thank you for the submission of your manuscript to EMBO Molecular Medicine, and please accept my sincere apologies for the unusual delay in getting back to you. As explained in a previous correspondence, we received one report early February, and have since then been waiting for the other 2 referees to provide feedback. We have now finally received an additional report. Given that both referees provide similar recommendations, we prefer to make a decision now in order to avoid further delay in the process. Should referee #3 provide a report, we will send it to you, with the understanding that we will not ask you extensive additional experiments.

As you will see from the reports below, both referees acknowledge the interest of the study and are supporting publication of your work pending appropriate minor revisions.

Addressing the reviewers' concerns in full will be necessary for further considering the manuscript in our journal, and acceptance of the manuscript will entail a second round of review. EMBO Molecular Medicine encourages a single round of revision only and therefore, acceptance or rejection of the manuscript will depend on the completeness of your responses included in the next, final version of the manuscript. For this reason, and to save you from any frustrations in the end, I would strongly advise against returning an incomplete revision.

If you would like to discuss further the points raised by the referees, I am available to do so via email or video. Let me know if you are interested in this option.

Revised manuscripts should be submitted within three months of a request for revision; they will otherwise be treated as new submissions, except under exceptional circumstances in which a short extension is obtained from the editor.

When submitting your revised manuscript, please carefully review the instructions that follow below. We perform an initial quality control of all revised manuscripts before re-review; failure to include requested items will delay the evaluation of your revision.

We require:

1) A .docx formatted version of the manuscript text (including legends for main figures, EV figures and tables). Please make sure that the changes are highlighted to be clearly visible.

2) Individual production quality figure files as .eps, .tif, .jpg (one file per figure). For guidance, download the 'Figure Guide PDF' (https://www.embopress.org/page/journal/17574684/authorguide#figureformat).

3) At EMBO Press we ask authors to provide source data for the main figures. Our source data coordinator will contact you to discuss which figure panels we would need source data for and will also provide you with helpful tips on how to upload and organize the files.

4) A .docx formatted letter INCLUDING the reviewers' reports and your detailed point-by-point responses to their comments. As part of the EMBO Press transparent editorial process, the point-by-point response is part of the Review Process File (RPF), which will be published alongside your paper.

5) A complete author checklist, which you can download from our author guidelines (https://www.embopress.org/page/journal/17574684/authorguide#submissionofrevisions). Please insert information in the checklist that is also reflected in the manuscript. The completed author checklist will also be part of the RPF.

6) It is mandatory to include a 'Data Availability' section after the Materials and Methods. Before submitting your revision, primary datasets produced in this study need to be deposited in an appropriate public database, and the accession numbers and database listed under 'Data Availability'. Please remember to provide a reviewer password if the datasets are not yet public (see https://www.embopress.org/page/journal/17574684/authorguide#dataavailability).

In case you have no data that requires deposition in a public database, please state so in this section. Note that the Data Availability Section is restricted to new primary data that are part of this study.

7) For data quantification: please specify the name of the statistical test used to generate error bars and P values, the number (n) of independent experiments (specify technical or biological replicates) underlying each data point and the test used to calculate p-values in each figure legend. The figure legends should contain a basic description of n, P and the test applied. Graphs must include a description of the bars and the error bars (s.d., s.e.m.). Please provide exact p values.

8) We replaced Supplementary Information with Expanded View (EV) Figures and Tables that are collapsible/expandable online. A maximum of 5 EV Figures can be typeset. EV Figures should be cited as 'Figure EV1, Figure EV2" etc... in the text and their respective legends should be included in the main text after the legends of regular figures.
- For the figures that you do NOT wish to display as Expanded View figures, they should be bundled together with their legends in a single PDF file called *Appendix*, which should start with a short Table of Content. Appendix figures should be referred to in the main text as: "Appendix Figure S1, Appendix Figure S2" etc.
- Additional Tables/Datasets should be labeled and referred to as Table EV1, Dataset EV1, etc. Legends have to be provided in a separate tab in case of .xls files. Alternatively, the legend can be supplied as a separate text file (README) and zipped together with the Table/Dataset file.
See detailed instructions here:
https://www.embopress.org/page/journal/17574684/authorguide#expandedview

9) The paper explained: EMBO Molecular Medicine articles are accompanied by a summary of the articles to emphasize the major findings in the paper and their medical implications for the non-specialist reader. Please provide a draft summary of your article highlighting
- the medical issue you are addressing,
- the results obtained and
- their clinical impact.
This may be edited to ensure that readers understand the significance and context of the research. Please refer to any of our published articles for an example.

10) For more information: There is space at the end of each article to list relevant web links for further consultation by our readers. Could you identify some relevant ones and provide such information as well? Some examples are patient associations, relevant databases, OMIM/proteins/genes links, author's websites, etc...

11) Author contributions: CRediT has replaced the traditional author contributions section because it offers a systematic machine readable author contributions format that allows for more effective research assessment. Please remove the Authors Contributions from the manuscript and use the free text boxes beneath each contributing author's name in our system to add specific details on the author's contribution. More information is available in our guide to authors.

12) Disclosure statement and competing interests: We updated our journal's competing interests policy in January 2022 and request authors to consider both actual and perceived competing interests. Please review the policy https://www.embopress.org/competing-interests and update your competing interests if necessary.

13) Every published paper now includes a 'Synopsis' to further enhance discoverability. Synopses are displayed on the journal webpage and are freely accessible to all readers. They include a short stand first (maximum of 300 characters, including space) as well as 2-5 one-sentences bullet points that summarizes the paper. Please write the bullet points to summarize the key NEW findings. They should be designed to be complementary to the abstract - i.e. not repeat the same text. We encourage inclusion of key acronyms and quantitative information (maximum of 30 words / bullet point). Please use the passive voice. Please attach these in a separate file or send them by email, we will incorporate them accordingly.

Please also suggest a striking image or visual abstract to illustrate your article as a PNG file 550 px wide x 300-600 px high.

14) As part of the EMBO Publications transparent editorial process initiative (see our Editorial at http://embomolmed.embopress.org/content/2/9/329), EMBO Molecular Medicine will publish online a Review Process File (RPF) to accompany accepted manuscripts.
In the event of acceptance, this file will be published in conjunction with your paper and will include the anonymous referee reports, your point-by-point response and all pertinent correspondence relating to the manuscript. Let us know whether you agree with the publication of the RPF and as here, if you want to remove or not any figures from it prior to publication.
Please note that the Authors checklist will be published at the end of the RPF.

EMBO Molecular Medicine has a "scooping protection" policy, whereby similar findings that are published by others during review or revision are not a criterion for rejection. Should you decide to submit a revised version, I do ask that you get in touch after three months if you have not completed it, to update us on the status.

I look forward to receiving your revised manuscript.

Yours sincerely,

Lise Roth

Lise Roth, PhD
Senior Editor
EMBO Molecular Medicine

Please note: When submitting your revision you will be prompted to enter your funding and payment information. This will allow Wiley to send you a quote for the article processing charge (APC) in case of acceptance. This quote takes into account any reduction or fee waivers that you may be eligible for. Authors do not need to pay any fees before their manuscript is accepted and transferred to the publisher.

EMBO Press participates in many Publish and Read agreements that allow authors to publish Open Access with reduced/no publication charges. Check your eligibility: https://authorservices.wiley.com/author-resources/Journal-Authors/open-access/affiliation-policies-payments/index.html

***** Reviewer's comments *****

Referee #1 (Remarks for Author):

This manuscript, entitled "Multi-omic profiling reveals an RNA processing rheostat that predisposes to prostate cancer" by Stentenbachby et al, describes interesting findings that reveal that human disease-relevant mutations of ELAC2, 3' tRNA endoribonuclease, are predisposing factors of prostate cancer in mouse models. The models included homozygous prostate-specific knockout ELAC2 gene and full-gene homozygous ELAC2 A537T variant (equivalent to the human A541T substitution), along with their respective homozygous control mice. CRISPR-Cas9 genome editing was applied to introduce the A541T variant in the Elac2 gene, and homologous recombination to generate a prostate-specific deletion of Elac2. These were bred onto transgenic adenocarcinoma of mouse prostate (TRAMP) model that carries the T antigens of SV40 under the control of probasin promoter. To show the effect of these mutations on prostate cancer risk, these strains, having ELAC2 gene knockout and A537T variant, were examined for molecular and physiological characteristics. Through performing nuclear, mitochondrial transcriptome, proteome, small RNAs and miRNAs wide-screen studies, it was argued that reduced steady state levels and activity of ELAC2 predispose to prostate cancer by weakening the function of non-coding RNAs, such as tRNAs, long noncoding RNAs and microRNAs, which seem to alter immunometabolism.
This manuscript provides general, but extensive and valuable information about KO of ELAC2 and its A537T variant, found in the human population, that predispose to prostate cancer. However, the underlying precise molecular mechanism for the direct role of ELAC2 in prostate cancer remains unresolved. Though, the authors mentioned in the abstract that the molecular mechanism is determined. In general, the application of multi-omic profiling does not delineate the exact molecular mechanism responsible for a molecular process. Elucidation of the molecular mechanism by which ELAC2 directly causes prostate cancer will need additional functional approaches, including comparative enzymatic assays of the wild type and disease-relevant mutants of ELAC2 in vitro and in vivo, combined with rescue genetic experiments of the mutants by the use of wild type ELAC2 gene both in mice and then in vitro. It will also be helpful to substitute, if feasible, the mouse ELAC2 gene with human wild type and disease-related mutant ELAC2 genes. Nevertheless, the data presented in the manuscript is sufficient to support the conclusion that knockout of the 3' tRNA endoribonuclease and expression of clinically-relevant mutants of this endoribonuclease, promote prostate cancer in model animals.

Comments:

In the Abstract, it is written that "....the ELAC2 variant is a predisposing factor for prostate cancer and provides a detailed molecular mechanism and physiologically relevant models of this cancer". What detailed molecular mechanism is provided? In order to elucidate the molecular mechanism, relevant mutants of ELAC2 enzymes should be studied in their molecular context. Such as having a defined 3' end processing system of precursor tRNA and then examining the role of wild type versus mutant ELAC2 enzymes. Also, it is unclear in the case of prostate cancer if unprocessed precursor tRNAs with retained 3' trailers are oncogenic molecules. If not, then these immature precursor tRNAs exert their effect via different pathways, such as DNA damage and repair.
The 3' tRNA endoribonuclease cleaves RNAs other than precursor tRNAs. Long noncoding RNAs having tRNA-like structures, like MALAT1 and MEN (for example, see PMID: 34001866, PMID: 19041754), are cleaved by the 3' tRNA endoribonuclease. Thus, these transcripts are also candidates for being involved in prostate cancer, as these long noncoding RNA were shown to be implicated in different malignancies.
It will be constructive to tune down the conclusion that defects in 3' trailer cleavage of precursor tRNAs lead to prostate cancer.

Specific notes:
In page 3, The following long sentence may be shortened or rephrased: Numerous genetic and genome wide-association studies (GWAS) in the last decade have implicated specific genes in the development of sporadic and, in particular, hereditary

prostate cancer, including hereditary prostate cancer 1 gene (HPC1)/ Ribonuclease L (RNASEL) (1q24-25) and HPC2 or ElaC Homolog Protein 2 (ELAC2) (17p12), both of which are the most relevant candidate genes for prostate cancer predisposition.
Figure 2C: If a longer exposure of ELAC2 band is available, that will be helpful to highlight the KO vs WT and A537T.
Figure 7G legend: " (G) ELAC2 is a regulates the levels of...". Edit.

Referee #2 (Comments on Novelty/Model System for Author):

The authors looked into how ELAC2 (gene coding for a nuclear and mitochondrial tRNA endonuclease) mutations were functionally contributing to the risk of prostate cancer. They therefore developed four in vivo mouse models and thoroughly examined and interrogated their molecular and physiological characteristics.
The authors provide with compelling functional and mechanistic evidence showing that an ELAC2 A537T variant predisposes to prostate cancer. They provide with comprehensive evidence to show that the nuclear and mitochondrial transcriptomes and proteomes, miRNAs and small RNAs correlate a direct link between reduced ELAC2 levels and activity to predisposition to prostate cancer. This variant alters the maturation and therefore the function of non-coding RNAs such as tRNAs, lncRNAs and miRNAs which in turn alter immunometabolism.
Specifically, the authors demonstrate how ELAC2 mutations cause prostate enlargement and inflammation which leads to prostate cancer on a TRAMP background. Moreover, the demonstrate how the ELAC2 A537T variant leads to inflammation, cell proliferation and differentiation in the prostate a direct impact of impaired tRNA processing that as a result causes transcriptome-wide changes ultimately promoting prostate tumorigenesis. Their analysis indicates that defects in aerobic metabolism drive prostate tumorigenesis via the PERK-mediated unfolded protein response.
The authors further indicate that ELAC2 is a rheostat that regulates miRNA homeostasis and as a result the ELAC2 variant leads to miRNA imbalance and potentiation of tumorigenesis in the prostates of the A537T-TRAMP mice.
Lastly the authors made a rather important and unexpected discovery. They show that ELAC2 A537T variant leads to prostate tumorigenesis by reducing, as of previously undescribed, ELAC2 interactions with SFPQ and NONO, proteins essential in multiple processes RNA-binding proteins, thus affecting RNA and energy metabolism.

This work is of great significance because it establishes new and physiologically relevant models of prostate tumorigenesis and cancer, and could be of considerable impact in drug screens and therapeutic development as well as to the continued investigation of prostate cancer biology. The work is of exceptional quality and no edits are recommended.

Point-by-point response to the reviewers' comments

Reviewer #1:

This manuscript, entitled "Multi-omic profiling reveals an RNA processing rheostat that predisposes to prostate cancer" by Stentenbachby et al, describes interesting findings that reveal that human disease-relevant mutations of ELAC2, 3' tRNA endoribonuclease, are predisposing factors of prostate cancer in mouse models. The models included homozygous prostate-specific knockout ELAC2 gene and full-gene homozygous ELAC2 A537T variant (equivalent to the human A541T substitution), along with their respective homozygous control mice. CRISPR-Cas9 genome editing was applied to introduce the A541T variant in the Elac2 gene, and homologous recombination to generate a prostate-specific deletion of Elac2. These were bred onto transgenic adenocarcinoma of mouse prostate (TRAMP) model that carries the T antigens of SV40 under the control of probasin promoter. To show the effect of these mutations on prostate cancer risk, these strains, having ELAC2 gene knockout and A537T variant, were examined for molecular and physiological characteristics. Through performing nuclear, mitochondrial transcriptome, proteome, small RNAs and miRNAs wide-screen studies, it was argued that reduced steady state levels and activity of ELAC2 predispose to prostate cancer by weakening the function of non-coding RNAs, such as tRNAs, long noncoding RNAs and microRNAs, which seem to alter immunometabolism.
This manuscript provides general, but extensive and valuable information about KO of ELAC2 and its A537T variant, found in the human population, that predispose to prostate cancer. However, the underlying precise molecular mechanism for the direct role of ELAC2 in prostate cancer remains unresolved. Though, the authors mentioned in the abstract that the molecular mechanism is determined. In general, the application of multi-omic profiling does not delineate the exact molecular mechanism responsible for a molecular process. Elucidation of the molecular mechanism by which ELAC2 directly causes prostate cancer will need additional functional approaches, including comparative enzymatic assays of the wild type and disease-relevant mutants of ELAC2 in vitro and in vivo, combined with rescue genetic experiments of the mutants by the use of wild type ELAC2 gene both in mice and then in vitro. It will also be helpful to substitute, if feasible, the mouse ELAC2 gene with human wild type and disease-related mutant ELAC2 genes. Nevertheless, the data presented in the manuscript is sufficient to support the conclusion that knockout of the 3' tRNA endoribonuclease and expression of clinically-relevant mutants of this endoribonuclease, promote prostate cancer in model animals.

We thank this reviewer for their advice and support of this work and address their insightful comments below. We have already created four new models of prostate inflammation and tumorigenesis, characterised their phenotypes, and generated transcriptomic data showing accumulation of 3′ tRNA precursors *in vivo*, therefore generating additional models where the mouse gene is substituted with the human gene is beyond the scope of the current study and would take several additional years of work. The point mutation introduced into the mouse ELAC2 gene alters an amino acid that is conserved in human ELAC2 and predisposes to prostate cancer, hence why we carried out this, more physiologically relevant, knock in, so that we do not introduce additional variables and potential

incompatibilities that would occur if the human equivalent of the whole gene was introduced in the background of the mouse genome. This alternative approach is a nice suggestion from this reviewer, and hybrid data like this would complement our study as an interesting project for the future.

The *in vitro* cleavage activity of recombinantly produced ELAC2 bearing the A541T variant has previously been analysed (PMID: 12711671) and no significant change was observed compared to wild-type ELAC2, when equivalent amounts of protein were tested. In our work we show that the A541T variant reduces the stability of ELAC2 by ~30%, which results in decreased *in vivo* cleavage of ELAC2 target RNAs in the prostates of our different mouse models. Reduction in RNA processing as a consequence of the A541T variant explains its presence in the human population without the dramatic consequences of disease mutations that cause loss or severe reduction of ELAC2 activity resulting in early-onset cardiomyopathies (PMID: 23849775). Nevertheless, the long-term consequences of the changes induced by the A541T variant in combination with a second oncogenic insult manifest in altered cell signalling, inflammation and predisposition to prostate cancer.

Comments:
In the Abstract, it is written that "....the ELAC2 variant is a predisposing factor for prostate cancer and provides a detailed molecular mechanism and physiologically relevant models of this cancer". What detailed molecular mechanism is provided? In order to elucidate the molecular mechanism, relevant mutants of ELAC2 enzymes should be studied in their molecular context. Such as having a defined 3' end processing system of precursor tRNA and then examining the role of wild type versus mutant ELAC2 enzymes. Also, it is unclear in the case of prostate cancer if unprocessed precursor tRNAs with retained 3' trailers are oncogenic molecules. If not, then these immature precursor tRNAs exert their effect via different pathways, such as DNA damage and repair.
The 3' tRNA endoribonuclease cleaves RNAs other than precursor tRNAs. Long noncoding RNAs having tRNA-like structures, like MALAT1 and MEN (for example, see PMID: 34001866, PMID: 19041754), are cleaved by the 3' tRNA endoribonuclease. Thus, these transcripts are also candidates for being involved in prostate cancer, as these long noncoding RNA were shown to be implicated in different malignancies.
It will be constructive to tune down the conclusion that defects in 3' trailer cleavage of precursor tRNAs lead to prostate cancer.

We provide the molecular changes that lead to prostate tumorigenesis in the four different models relative to their two controls in the manuscript, and therefore we have clarified this in the revised manuscript as suggested by this reviewer. ELAC2 is not required for processing of the *MALAT1* lncRNA, this is achieved by RNase P at it's 3′ end (PMID: 34253686 and PMID: 19041754), consistent with our RNA sequencing findings. We did not mean to imply that the accumulation of 3' precursors is causing the tumorigenicity, rather that the lack of mature transcripts as a consequence of impaired RNA processing exerts effects on the pathways that are changing. We have edited the relevant text to clarify this distinction. In addition, we have edited this conclusion as suggested by the reviewer in the abstract and the concluding sentence in the introduction.

Specific notes:

In page 3, The following long sentence may be shortened or rephrased: Numerous genetic and genome wide-association studies (GWAS) in the last decade have implicated specific genes in the development of sporadic and, in particular, hereditary prostate cancer, including hereditary prostate cancer 1 gene (HPC1)/ Ribonuclease L (RNASEL) (1q24-25) and HPC2 or ElaC Homolog Protein 2 (ELAC2) (17p12), both of which are the most relevant candidate genes for prostate cancer predisposition.

We have shortened this sentence as suggested by breaking it into two sentences.

Figure 2C: If a longer exposure of ELAC2 band is available, that will be helpful to highlight the KO vs WT and A537T.

The blot included in this figure is the longest exposure, although there is some background, this is the only antibody on the market that can detect ELAC2 in mouse tissues.

Figure 7G legend: " (G) ELAC2 is a regulates the levels of...". Edit.

Thank you for spotting this typographical error, we have corrected this.

Reviewer #2 (Remarks to the Author):

The authors looked into how ELAC2 (gene coding for a nuclear and mitochondrial tRNA endonuclease) mutations were functionally contributing to the risk of prostate cancer. They therefore developed four in vivo mouse models and thoroughly examined and interrogated their molecular and physiological characteristics.

The authors provide with compelling functional and mechanistic evidence showing that an ELAC2 A537T variant predisposes to prostate cancer. They provide with comprehensive evidence to show that the nuclear and mitochondrial transcriptomes and proteomes, miRNAs and small RNAs correlate a direct link between reduced ELAC2 levels and activity to predisposition to prostate cancer. This variant alters the maturation and therefore the function of non-coding RNAs such as tRNAs, lncRNAs and miRNAs which in turn alter immunometabolism.

Specifically, the authors demonstrate how ELAC2 mutations cause prostate enlargement and inflammation which leads to prostate cancer on a TRAMP background. Moreover, the demonstrate how the ELAC2 A537T variant leads to inflammation, cell proliferation and differentiation in the prostate a direct impact of impaired tRNA processing that as a result causes transcriptome-wide changes ultimately promoting prostate tumorigenesis. Their analysis indicates that defects in aerobic metabolism drive prostate tumorigenesis via the PERK-mediated unfolded protein response.

The authors further indicate that ELAC2 is a rheostat that regulates miRNA homeostasis and as a result the ELAC2 variant leads to miRNA imbalance and potentiation of tumorigenesis in the prostates of the A537T-TRAMP mice.

Lastly the authors made a rather important and unexpected discovery. They show that ELAC2 A537T variant leads to prostate tumorigenesis by reducing, as of previously undescribed, ELAC2 interactions with SFPQ and NONO, proteins essential in multiple processes RNA-binding proteins, thus affecting RNA and energy metabolism.

This work is of great significance because it establishes new and physiologically relevant models of prostate tumorigenesis and cancer, and could be of considerable impact in drug screens and therapeutic development as well as to the continued investigation of prostate cancer biology. The work is of exceptional quality and no edits are recommended.

We thank this reviewer for assessing this study and for their support of our work.

30th Mar 2023

Dear Prof. Filipovska,

Thank you for the submission of your revised manuscript to EMBO Molecular Medicine. I am pleased to inform you that we will be able to accept your manuscript once the following editorial points will be addressed:

1/ Main manuscript text:
- Please accept the changes and only keep in track changes mode any new modification.
- In the figure legends (and/or in the materials and methods/statistical analyses), please define the bars and error bars, e.g. mean {plus minus} SD, as indicated by the data editors.
- Materials and methods:
o Mitochondrial isolation: you indicated "as described previously [...] with some modifications", please clarify.
o Statistical analysis: please include a statement about randomization and inclusion/exclusion criteria.

2/ Figures and Appendix:
We note that your appendix comprises 11 figures. If you wish, you could make some of these figures "Expanded View (EV) Figures", that are collapsible/expandable online. A maximum of 5 EV Figures can be typeset. EV Figures should be cited as 'Figure EV1, Figure EV2" etc... in the text and their respective legends should be included in the main text after the legends of regular figures.
The remaining figures would then constitute the Appendix, which should start with a short Table of Content. Appendix figures are referred to in the main text as: "Appendix Figure S1, Appendix Figure S2" etc.

3/ Source Data:
Thank you for providing Source Data. Please also fill in the Source Data checklist.

4/ Checklist:
- Newly created materials: it seems in your case no restriction apply, please adjust accordingly.
- Experimental study design and statistics: Please include a sentence for Randomization and Exclusion Criteria. (i.e. "No randomization was done" and "No animals were excluded from the analyses").
- Reporting: Please include a sentence regarding guidelines (i.e. "no specific guidelines was followed").

5/ As part of the EMBO Publications transparent editorial process initiative (see our Editorial at http://embomolmed.embopress.org/content/2/9/329), EMBO Molecular Medicine will publish online a Review Process File (RPF) to accompany accepted manuscripts.
This file will be published in conjunction with your paper and will include the anonymous referee reports, your point-by-point response and all pertinent correspondence relating to the manuscript. Let us know whether you agree with the publication of the RPF.
Please note that the Authors checklist will be published at the end of the RPF, and that all datasets must be publicly available before online publication of the manuscript.

I look forward to receiving your revised manuscript.

Yours sincerely,

Lise Roth

Lise Roth, PhD
Senior Editor
EMBO Molecular Medicine

The system will prompt you to fill in your funding and payment information. This will allow Wiley to send you a quote for the article processing charge (APC) in case of acceptance. This quote takes into account any reduction or fee waivers that you may be eligible for. Authors do not need to pay any fees before their manuscript is accepted and transferred to our publisher.

The authors have addressed all minor editorial requests.

2nd Revision - Editorial Decision 31st Mar 2023

31st Mar 2023

Dear Prof. Filipovska,

Thank you for submitting the revised files. I am pleased to inform you that your manuscript is accepted for publication and is now being sent to our publisher to be included in the next available issue of EMBO Molecular Medicine.

We would like to remind you that as part of the EMBO Publications transparent editorial process initiative, EMBO Molecular Medicine will publish a Review Process File online to accompany accepted manuscripts. If you do NOT want the file to be published, please immediately inform the editorial office via e-mail.

Please read below for additional IMPORTANT information regarding your article, its publication and the production process.

Congratulations on your interesting work!

With kind regards,

Lise Roth

Lise Roth, Ph.D
Senior Editor
EMBO Molecular Medicine

Follow us on Twitter @EmboMolMed
Sign up for eTOCs at embopress.org/alertsfeeds

*** *** *** IMPORTANT INFORMATION *** *** ***

SPEED OF PUBLICATION
The journal aims for rapid publication of papers, using using the advance online publication "Early View" to expedite the process: A properly copy-edited and formatted version will be published as "Early View" after the proofs have been corrected. Please help the Editors and publisher avoid delays by providing e-mail address(es), telephone and fax numbers at which author(s) can be contacted.

Should you be planning a Press Release on your article, please get in contact with embomolmed@wiley.com as early as possible, in order to coordinate publication and release dates.

LICENSE AND PAYMENT:

All articles published in EMBO Molecular Medicine are fully open access: immediately and freely available to read, download and share.

EMBO Molecular Medicine charges an article processing charge (APC) to cover the publication costs. You, as the corresponding author for this manuscript, should have already received a quote with the article processing fee separately. Please let us know in case this quote has not been received.

Once your article is at Wiley for editorial production you will receive an email from Wiley's Author Services system, which will ask you to log in and will present you with the publication license form for completion. Within the same system the publication fee can be paid by credit card, an invoice, pro forma invoice or purchase order can be requested.

Payment of the publication charge and the signed Open Access Agreement form must be received before the article can be published online.

PROOFS

You will receive the proofs by e-mail approximately 2 weeks after all relevant files have been sent o our Production Office.

Please return them within 48 hours and if there should be any problems, please contact the production office at embopressproduction@wiley.com.

Please inform us if there is likely to be any difficulty in reaching you at the above address at that time. Failure to meet our deadlines may result in a delay of publication.

All further communications concerning your paper proofs should quote reference number EMM-2023-17463-V3 and be directed to the production office at embopressproduction@wiley.com.

EMBO Press Author Checklist

Corresponding Author Name: Aleksandra Filipovska
Journal Submitted to: EMBO Molecular Medicine
Manuscript Number: EMM-2023-17463

USEFUL LINKS FOR COMPLETING THIS FORM
The EMBO Journal - Author Guidelines
EMBO Reports - Author Guidelines
Molecular Systems Biology - Author Guidelines
EMBO Molecular Medicine - Author Guidelines

Reporting Checklist for Life Science Articles (updated January
This checklist is adapted from Materials Design Analysis Reporting (MDAR) Checklist for Authors. MDAR establishes a minimum set of requirements in transparent reporting in the life sciences (see Statement of Task: 10.31222/osf.io/9sm4x). Please follow the journal's guidelines in preparing your
Please note that a copy of this checklist will be published alongside your article.

Abridged guidelines for figures
1. Data
The data shown in figures should satisfy the following conditions:
- the data were obtained and processed according to the field's best practice and are presented to reflect the results of the experiments in an accurate and unbiased manner.
- ideally, figure panels should include only measurements that are directly comparable to each other and obtained with the same assay.
- plots include clearly labeled error bars for independent experiments and sample sizes. Unless justified, error bars should not be shown for technical
- if n<5, the individual data points from each experiment should be plotted. Any statistical test employed should be justified.
- Source Data should be included to report the data underlying figures according to the guidelines set out in the authorship guidelines on Data

2. Captions
Each figure caption should contain the following information, for each panel where they are relevant:
- a specification of the experimental system investigated (eg cell line, species name).
- the assay(s) and method(s) used to carry out the reported observations and measurements.
- an explicit mention of the biological and chemical entity(ies) that are being measured.
- an explicit mention of the biological and chemical entity(ies) that are altered/varied/perturbed in a controlled manner.
- the exact sample size (n) for each experimental group/condition, given as a number, not a range;
- a description of the sample collection allowing the reader to understand whether the samples represent technical or biological replicates (including how many animals, litters, cultures, etc.).
- a statement of how many times the experiment shown was independently replicated in the laboratory.
- definitions of statistical methods and measures:
  ommon tests su h as t test lease s e i hether aired vs. un aired sim le tests il o on and ann hitne tests an e unambiguously identified by name only, but more complex techniques should be described in the methods section;
  - are tests one-sided or two-sided?
  - are there adjustments for multiple comparisons?
  - exact statistical test results, e.g., P values = x but not P values < x;
  - definition of 'center values' as median or average;
  - definition of error bars as s.d. or s.e.m.

Please complete ALL of the questions below.
Select "Not Applicable" only when the requested information is not relevant for your study.

Materials

Newly Created Materials	Information included in the manuscript?	In which section is the information available? (Reagents and Tools Table, Materials and Methods, Figures, Data Availability Section)
New materials and reagents need to be available; do any restrictions apply?	Yes	No restrictions apply. Genomic and proteomic data are available and will be publically released upon publication and their deposit IDs are listed in the Data Availability Section

Antibodies	Information included in the manuscript?	In which section is the information available? (Reagents and Tools Table, Materials and Methods, Figures, Data Availability Section)
For **antibodies** provide the following information:<br>- Commercial antibodies: RRID (if possible) or supplier name, catalogue number and or/clone number<br>- Non-commercial: RRID or citation	Yes	Materials and Methods section under Immunoblotting.

DNA and RNA sequences	Information included in the manuscript?	In which section is the information available? (Reagents and Tools Table, Materials and Methods, Figures, Data Availability Section)
**Short novel DNA or RNA including primers, probes**: provide the sequences.	Not Applicable	

Cell materials	Information included in the manuscript?	In which section is the information available? (Reagents and Tools Table, Materials and Methods, Figures, Data Availability Section)
**Cell lines:** Provide species information, strain. Provide accession number in repository **OR** supplier name, catalog number, clone number, and/**OR** RRID.	Yes	LNCap cells from ATCC (CRL-1740) listed on page 26 in Materials and Methods section
**Primary cultures:** Provide species, strain, sex of origin, genetic modification status.	Not Applicable	
Report if the cell lines were recently **authenticated** (e.g., by STR profiling) and tested for mycoplasma contamination.	Yes	Authenticity was confirmed by STR profiling. Cells were tested and confirmed negative for mycoplasma contamination,listed on page 26 in Materials and Methods section.

Experimental animals	Information included in the manuscript?	In which section is the information available? (Reagents and Tools Table, Materials and Methods, Figures, Data Availability Section)
**Laboratory animals or Model organisms:** Provide species, strain, sex, age, genetic modification status. Provide accession number in repository **OR** supplier name, catalog number, clone number, **OR** RRID.	Yes	Materials and methods.
**Animal observed in or captured from the field:** Provide species, sex, and age where possible.	Not Applicable	
Please detail **housing and husbandry conditions**.	Yes	Materials and methods.

Plants and microbes	Information included in the manuscript?	In which section is the information available? (Reagents and Tools Table, Materials and Methods, Figures, Data Availability Section)
**Plants:** provide species and strain, ecotype and cultivar where relevant, unique accession number if available, and source (including location for collected wild specimens).	Not Applicable	
**Microbes:** provide species and strain, unique accession number if available, and source.	Not Applicable	

Human research participants	Information included in the manuscript?	In which section is the information available? (Reagents and Tools Table, Materials and Methods, Figures, Data Availability Section)
If collected and within the bounds of privacy constraints report on age, sex and gender or ethnicity for all study participants.	Not Applicable	

Core facilities	Information included in the manuscript?	In which section is the information available? (Reagents and Tools Table, Materials and Methods, Figures, Data Availability Section)
If your work benefited from core facilities, was their service mentioned in the acknowledgments section?	Yes	Acknowledgements section.

Design

Study protocol	Information included in the manuscript?	In which section is the information available? (Reagents and Tools Table, Materials and Methods, Figures, Data Availability Section)

If study protocol has been **pre-registered, provide DOI in the manuscript**. For clinical trials, provide the trial registration number **OR** cite DOI.	Not Applicable	
Report the **clinical trial registration number** (at ClinicalTrials.gov or equivalent), where applicable.	Not Applicable	

Laboratory protocol	Information included in the manuscript?	In which section is the information available? (Reagents and Tools Table, Materials and Methods, Figures, Data Availability Section)
Provide DOI OR other citation details if **external detailed step-by-step protocols** are available.	Not Applicable	

Experimental study design and statistics	Information included in the manuscript?	In which section is the information available? (Reagents and Tools Table, Materials and Methods, Figures, Data Availability Section)
Include a statement about **sample size** estimate even if no statistical methods were used.	Yes	Sample sizes are incldued in all Figure legends.
ere an ste s ta en to minimi e the e e ts o su e tive ias hen allocating animals/samples to treatment (e.g. **randomization procedure**)? If yes, have they been described?	Not Applicable	No randomization was done.
Include a statement about **blinding** even if no blinding was done.	Yes	Materials and Methods section.
Describe **inclusion/exclusion criteria** if samples or animals were excluded rom the anal sis. ere the riteria re esta lished<br><br>If sample or data points were omitted from analysis, report if this was due to attrition or intentional exclusion and provide justification.	Not Applicable	No animals were excluded from the analyses.
For every figure, are **statistical tests** justified as appropriate? Do the data meet the assumptions of the tests (e.g., normal distribution)? Describe any methods used to assess it. Is there an estimate of variation within each group of data? Is the variance similar between the groups that are being statistically compared?	Yes	Materials and Methods section.

Sample definition and in-laboratory replication	Information included in the manuscript?	In which section is the information available? (Reagents and Tools Table, Materials and Methods, Figures, Data Availability Section)
In the figure legends: state number of times the experiment was **replicated** in laboratory.	Yes	Figure Legends.
In the figure legends: define whether data describe **technical or biological replicates**.	Yes	All data are biological repicates and this is stated in the Figure Legends.

Ethics

Ethics	Information included in the manuscript?	In which section is the information available? (Reagents and Tools Table, Materials and Methods, Figures, Data Availability Section)
Studies involving **human participants**: State details of **authority granting ethics approval** (IRB or equivalent committee(s), provide reference number for approval.	Not Applicable	
Studies involving **human participants**: Include a statement confirming that **informed consent** was obtained from all subjects and that the experiments on ormed to the rin i les set out in the e laration o elsin i and the e artment o ealth and uman ervi es elmont e ort.	Not Applicable	
Studies involving **human participants:** For publication of **patient photos**, include a statement confirming that consent to publish was obtained.	Not Applicable	
Studies involving experimental **animals**: State details of **authority granting ethics approval** (IRB or equivalent committee(s), provide reference number for approval. Include a statement of compliance with ethical regulations.	Yes	Materials and Methods section.
Studies involving **specimen and field samples:** State if relevant **permits** obtained, provide details of authority approving study; if none were required, explain why.	Not Applicable	

Dual Use Research of Concern (DURC)	Information included in the manuscript?	In which section is the information available? (Reagents and Tools Table, Materials and Methods, Figures, Data Availability Section)
Could your study fall under dual use research restrictions? Please check biosecurity documents and list of **select agents and toxins** (CDC): https://www.selectagents.gov/sat/list.htm	Not Applicable	
If you used a select agent, is the security level of the lab appropriate and reported in the manuscript?	Not Applicable	
If a study is subject to dual use research of concern regulations, is the name of the **authority granting approval and reference number** for the regulatory approval provided in the manuscript?	Not Applicable	

Reporting

The MDAR framework recommends adoption of discipline-specific guidelines, established and endorsed through community initiatives. Journals have their own policy about requiring specific guidelines and recommendations to complement MDAR.

Adherence to community standards	Information included in the manuscript?	In which section is the information available? (Reagents and Tools Table, Materials and Methods, Figures, Data Availability Section)
State if relevant guidelines or checklists (e.g., **ICMJE, MIBBI, ARRIVE, PRISMA**) have been followed or provided.	Not Applicable	No specific guidelines were followed
For **tumor marker prognostic studies,** we recommend that you follow the **REMARK** reporting guidelines (see link list at top right). See author guidelines, under 'Reporting Guidelines'. Please confirm you have followed these guidelines.	Not Applicable	
For **phase II and III randomized controlled trials**, please refer to the **CONSORT** flow diagram (see link list at top right) and submit the CONSORT checklist (see link list at top right) with your submission. See author guidelines, under 'Reporting Guidelines'. Please confirm you have submitted this list.	Not Applicable	

Data Availability

Data availability	Information included in the manuscript?	In which section is the information available? (Reagents and Tools Table, Materials and Methods, Figures, Data Availability Section)
ave **primary datasets** been deposited according to the journal's guidelines (see 'Data Deposition' section) and the respective accession numbers provided in the Data Availability Section?	Yes	Data Availablity Section.
ere **human clinical and genomic datasets** deposited in a public access-controlled repository in accordance to ethical obligations to the patients and to the applicable consent agreement?	Not Applicable	
Are **computational models** that are central and integral to a study available ithout restri tions in a ma hine reada le orm ere the relevant accession numbers or links provided?	Not Applicable	
If publicly available data were reused, provide the respective **data citations in the reference list**.	Not Applicable	
